# Supplementary material for: Simultaneous Enantiodivergent Synthesis of Diverse Lactones and Lactams via Sequential One-Pot Enzymatic Kinetic Resolution–Ring-Closing Metathesis Reactions
Source: Molecules. 2022 Nov 9;27(22):7696. doi: 10.3390/molecules27227696 (PMC9698981; doi:10.3390/molecules27227696)
Supplement: Supplementary file 1 [file molecules-27-07696-s001.zip › molecules-1986017-supplementary.pdf]

Supporting Information

Table of contents:

1. **General remarks**
2. **The optimization study of EKR-RCM reaction—the impact of alkoxy group donor**
3. **The determination of absolute configuration of compounds 1a, 3a, 4a, 5a, 7a, 8a**
4. **General procedures for synthesis of compounds 1, 3, 4, 5, 7, 8, 9, 10**
5. **Analytical data of compounds 1, 3, 4, 5, 7, 8, 9, 10**
6. **^{1}H NMR and ^{13}C NMR spectra**
7. **HPLC traces**
8. **References**

1. General experimental details and materials

All the chemicals were obtained from commercial sources. The solvents were of analytical grade. NMR spectra were recorded in $CDCl_3$ with TMS as an internal standard using Bruker 400 MHz and Varian 200 MHz spectrometers. The chemical shifts are reported in ppm (δ scale) and the coupling constants (J) are given in hertz (Hz). All the reactions were monitored by TLC on Merck silica gel Plates 60 F_{254}. Column chromatography was performed on Merck silica gel 60/230–400 mesh.

2. The optimization study of EKR-RCM reaction—the impact of alkoxy group donor

We have also tested if analogues of allyl alcohol can be used as alkoxy group donors for the enzymatic transesterification of vinyl 3-phenyl-4-pentenoate (**1**). The reactions were carried out in the presence of Novozym in toluene at 70 °C during 24 hours. As alkoxy group donors we have tested allyl alcohol (**2a**), allyl acetate (**2b**), 3-methyl-3-buten-1-ol (**2c**), and cinnamyl alcohol (**2d**). The results summarized in Table S1 are measured with reference to lactones **4a** and **5a** obtained after subsequent ring-closing reaction.

Table S1. The influence of an alkoxy group donor on enzymatic transesterification of ester **1a**.[a]

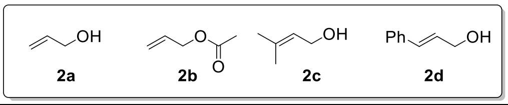

Entry	Donor	Yield of **4a** [%][b]	e.e.$_S$ [%][c]	Yield of **5a** [%][b]	e.e.$_P$ [%][c]	E[d]
1	**2a**	57	55	35	99	>200
2	**2a** (48 hrs)	47	99	43	99	>200
3	**2b**	41	38	47	27	2
4	**2c**	61	42	28	97	99
5	**2d**	30	0	58	0	nd

[a] Reactions conditions: **1a** (1 equiv.), **2** (2 equiv.), Novozym (10 mg), anhydrous toluene (c = 0.01), 70 °C, 24 hrs, inert atmosphere; then removal of enzyme and solvent, the mixture of **1a** and **3a**, Grubbs 2nd catalyst (5 mol%), anhydrous toluene (c = 0.01), 16 hrs, inert atmosphere. [b] Yield of isolated product. [c] Determined by HPLC on a Chiralpak AD-H column. [d] E was calculated according to the equation: $E=\ln[(e.e._P(1-e.e._S)/(e.e._P+e.e._S))]/\ln[(e.e._P(1+e.e._S)/(e.e._P+e.e._S))]$.

The transesterification of ester **1a** with allyl alcohol (**2a**) and subsequent RCM reaction resulted in the formation of six-membered lactone 4a with 57% yield and 55% of enantiomeric excess and an enatiopure lactone (*R*)-**5** with 35% yield (Table S1, entry 1). When the reaction time was extended to 48 hours, both lactones were obtained as single enantiomers: (*S*)-**4a** and (*R*)-**5a** (Table S1, entry 2). When allyl acetate (**2b**) was used as a donor the enantioselectivity of the enzymatic step dropped dramatically (Table S1, entry 3). The application of more sterically hindered 3-methyl-3-buten-1-ol (**2c**) resulted in a decrease in the reaction yield and enantioselectivity in comparison to allyl alcohol (Table S1, entry 4). When cinnamyl alcohol (**2d**) was used, both of products were obtained in racemic forms (Table S1, entry 5). Thus, allyl alcohol and Novozym in toluene as solvent at 70 °C within 48 hrs was set as the optimized conditions for studied enzymatic kinetic resolution.

3. The determination of absolute configuration of compounds 1a, 3a, 4a, 5a, 7a, 8a

The absolute configurations of amide (*R*)-**7a** was determined by comparing its optical rotation with the literature data[1]. The lactam **8a** was obtained *via* RCM reaction of (*R*)-**7a**; thus, the configuration was retained ((*R*)-**8**). On the basis of this data and the HPLC trances, the absolute configurations of compounds **1a**, **3a**, **4a**, and **5a** were determined.

4. General procedures for synthesis of compounds 1, 3, 4, 5, 7, 8, 9, 10

4.1. Synthesis of vinyl 3-substituted -4-pentenoate 1a–d

Potassium hydroxide (0.2 equiv.), palladium(II) acetate (0.1 equiv.), and 3-substituted-4-pentenoic acid **1a–d** (1 equiv.) were dissolved in vinyl acetate (100 equiv.), and the resulting reaction mixture was stirred overnight at 40 °C. The reaction mixture was cooled to room temperature, filtered over a Celite pad, and eluted with DCM. The solvents were removed using under reduced pressure. The crude product was purified by column chromatography on silica gel (hexanes/ethyl acetate).

Synthesis of 3-substituted-4-pentenoic acid: 3-substituted-4-pentenoic acid was synthesized and purified according to literature procedure.[2] The ^{1}H and ^{13}C NMR data were in accordance with those reported in the literature.

4.2. General procedure for the synthesis of compounds 3

To a solution of appropriate alcohol (**2a–d**) (1 equiv.) and 3-substituted-4-pentenoic acid **1a–d** (1.3 equiv.) in DCM (20 mL), DMAP (0.1 equiv.), and DCC (2.5 equiv.) were added. Then the mixture was stirred for 4 h at room temperature. After the reaction completed, the reaction mixture was diluted with DCM (50 mL), filtered through a celite pad, and washed with brine. The organic phase was dried over Na_2SO_4 and filtered. The filtrate was concentrated under vacuum. The crude residue was purified by column chromatography on silica gel (hexanes/ethyl acetate).

4.3. Synthesis of N-allyl-N-benzyl-3-substituted-4-pentenamides 7a–d

The mixed solution of DCC (1.5 equiv.) and DMAP (0.1 equiv.) in DCM was added to the solution of 3-substituted-4-pentenoic acid (**1a–d**) (1 equiv.) in DCM (3.6ml) while cooling in an ice bath. N-Allyl-N-benzylamine (1 equiv.) solution in DCM was then added, and the resulting mixture was stirred at room temperature for 1 h. Saturated ammonium chloride solution was added and the organic phase was separated. The aqueous phase was extracted with DCM (3x), and the organic phase was combined and dried over with anhydrous $MgSO_4$. The excess of solvent was removed under reduced pressure. The crude product was purified by column chromatography on silica gel (hexanes/ethyl acetate).

4.4. General procedure of the enzymatic kinetic resolution reaction (EKR) of 1a with 2

To the solution of vinyl 3-phenyl-4-pentenoate **1a** (0.1 mmol) in toluene or other solvent (2 mL), the appropriate alkoxy group donor (**2a–d**) (2 equiv.) and enzyme (10 mg) were added in 5 mL screwed vial. The reaction mixture was stirred for 24–48 hours at 40–70 °C. After cooling, the enzyme was filtered off and the solvent was evaporated. Then, to analyze the enantiomeric excesses, the products were transformed into corresponding lactones. The residue was dissolved in dry toluene (10 mL) under argon atmosphere and Grubbs second-generation catalyst was added (5 mol%). The reaction mixture was heated at 80 °C for 16 hours. The reaction mixture was cooled to room temperature and filtered over a Celite pad. The excess of solvent was removed under reduced pressure. The crude product was purified by column chromatography on silica gel (hexanes/ethyl acetate).

4.5. General procedure of the enzymatic kinetic resolution reaction (EKR) of 1a with 6

To the solution of vinyl 3-phenyl-4-pentenoate **1a** (0.1 mmol) in toluene (2 mL), the appropriate allylamine (**6**) (2 equiv.) and enzyme (10 mg) were added in 5 mL screwed vial. The reaction mixture was stirred for 24 hours at 70 °C. After cooling, crude product was purified by column chromatography (ethyl acetate/hexanes).

4.6. General procedure of the ring-closing metathesis reaction (RCM)

To a solution of a olefin (0.4 mmol) in dry solvent (40 mL) under argon atmosphere was added Grubbs second-generation catalyst (5 mol%). The reaction mixture was heated at 80 °C for 16 hours. The reaction mixture was cooled to room temperature and filtered over a Celite pad. The excess of solvent was removed under reduced pressure. The crude product was purified by column chromatography on silica gel (hexanes/ethyl acetate).

4.7. General procedure of the one-pot EKR-RCM

The mixture of vinyl 3-substituted-4-pentenoate **1a–d** (0.2 mmol), donor (**2** or **6**) (2 equiv.) and Novozym (10 mg) was stirred in dry toluene (10 mL) at 70 °C. After 24 hrs (for **6**) or 48 hrs (for **2**) Grubbs second-generation catalyst (5 mol%) was added. The reaction mixture was heated at 80 °C for 16 hours under argon atmosphere. The reaction mixture was cooled to room temperature and filtered over a Celite pad. The excess of solvent was removed under reduced pressure. The crude product was purified by column chromatography on silica gel (hexanes/ethyl acetate).

4.8. Synthesis of 4-phenyltetrahydro-2H-pyran-2-one 9

Pd/C (5 mol %) was added to a solution of lactone **4** (0,11 mmol, 1 equiv) in EtOAc (15 mL) at room temperature. This mixture was purged with H_2 and was stirred for 16 h. Next, the solvent was removed under reduced pressure. The crude product was purified by column chromatography on silica gel (hexanes/ethyl acetate).

4.9. Synthesis of methyl 5-oxo-3-phenylpentanoate 10

DMAP (0.15 mmol) was added to the solution of lactone **4** (0.15 mmol) in CH_3OH (1 mL). After stirring for 12 h at 35 °C, the crude mixture was purified by column chromatography (hexanes/ethyl acetate).

5. Analytical data of compounds 1, 3, 4, 5, 7, 8, 9, 10

Vinyl 3-phenyl-4-pentenoate (1a)

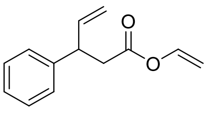

The product was isolated as a colorless oil from a silica column eluted by EtOAc/hexanes (1:10) in 62% yield (920 mg); ^{1}H NMR (400 MHz, $CDCl_3$): *δ* 7.28–7.23 (m, 2H), 7.18–7.12 (m, 4H), 5.99–5.86 (m, 1H), 5.06–4.97 (m, 2H), 4.79 (dd, J = 14.0, 1.6 Hz, 1H), 4.48 (dd, J = 6.3, 1.6 Hz, 1H), 3.84 (q, J = 7.4 Hz, 1H), 2.82–2.69 (m, 2H).; ^{13}C{H} NMR (100 MHz, $CDCl_3$): δ 168.9, 142.1, 141.1, 139.9, 128.7, 127.5, 126.9, 115.1, 97.8, 45.3, 39.9; HRMS (EI+, *m/z*): calcd. for $C_{13}H_{14}O_2$ $[M]^+$: 202.0994, found 202.0996. The ^{1}H and ^{13}C NMR data were in accordance with those reported in the literature.[3]

Vinyl 3-(4-bromophenyl)-4-pentenoate (1b)

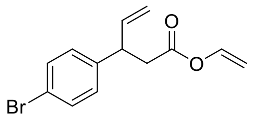

The product was isolated as a colorless oil from a silica column eluted by EtOAc/hexanes (1:10) in 57% yield (160 mg); ^{1}H NMR (200 MHz, $CDCl_3$): δ 7.40–7.33 (m, 2H), 7.15–7.07 (m, 1H), 7.07–6.98 (m, 2H), 5.96–5.79 (m, 1H), 5.14–5.49 (m, 2H), 4.78 (dd, J = 14.0, 1.7 Hz, 1H), 4.48 (dd, J = 6.3, 17, Hz, 1H), 3.79 (q, J = 7.4 Hz, 1H), 2.75–2.68 (m, 2H).; ^{13}C{H} NMR (50 MHz, $CDCl_3$): δ 168.6, 141.0, 140.3, 139.3, 129.3, 115.5, 98.3, 97.9, 44.6, 39.6.

Vinyl 3-(4-chlorophenyl)-4-pentenoate (1c)

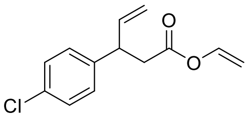

The product was isolated as a colorless oil from a silica column eluted by EtOAc/hexanes (1:10) in 52% yield (123 mg); ^{1}H NMR (200 MHz, $CDCl_3$): δ 7.21–7.04 (m, 5H), 5.96–5.80 (m, 1H), 5.11–4.92 (m, 2H), 4.79 (dd, J = 14.0, 1.8 Hz, 1H), 4.49 (dd, J = 6.3, 1.7 Hz, 1H), 3.81 (q, J = 7.4 Hz, 1H), 2.84–2.61 (m, 2H).; ^{13}C{H} NMR (50 MHz, $CDCl_3$): δ 168.6, 141.0, 139.4, 129.1, 128.9, 128.8, 115.5, 99.7, 97.9, 44.5, 39.7.

Vinyl 3-propyl-4-pentenoate (1)

The product was isolated as a colorless oil from a silica column eluted by EtOAc/hexanes (1:10) in 13% yield (120

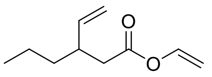

mg); ^{1}H NMR (400 MHz, $CDCl_3$): δ 7.19 (dd, J = 14.0, 6.3 Hz, 1H), 5.67–5.58 (m, 1H), 5.06–5.00 (m, 2H), 4.88 (dd, J = 14.0, 1.6 Hz, 1H), 4.54 (dd, J = 6.3, 1.4 Hz, 1H), 2.59–2.55 (m, 1H), 2.38 (ddd, J = 23.2, 15.0, 8.6 Hz, 2H), 1.35 (ddt, J = 20.4, 6.0, 5.4 Hz, 4H), 0.85 (t, J = 6.7 Hz, 3H); ^{13}C{H} NMR (100 MHz, $CDCl_3$): δ 169.6, 141.2, 140.7, 115.3, 97.5, 39.9, 39.8, 36.6, 20.0, 13.9. The ^{1}H and ^{13}C NMR data were in accordance with those reported in the literature.[3]

Allyl 3-phenyl-4-pentenoate (3a)

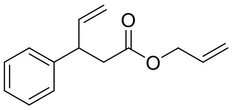

The product was isolated as a colorless oil from a silica column eluted by EtOAc/hexanes (1:10) in 87% yield (1.88 g); ^{1}H NMR (400 MHz, $CDCl_3$): δ 7.24–7.13 (m, 5H), 5.96–5.88 (m, 1H), 5.79–5.72 (m, 1H), 5.18–5.13 (m, 2H), 5.03–4.98 (m, 2H), 4.45 (dt, J = 5.7, 1.4 Hz, 2H), 3.82–3.80 (m, 1H), 2.76–2.64 (m, 2H); ^{13}C NMR (100 MHz, $CDCl_3$): δ 171.6, 142.4, 140.2, 132.1, 128.6, 127.5, 126.7, 118.1, 114.9, 65.1, 45.6, 40.2. The ^{1}H and ^{13}C NMR data were in accordance with those reported in the literature.[2]

Allyl 3-(4-bromophenyl)-4-pentenoate (3b)

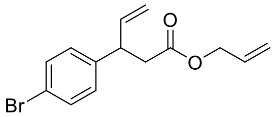

The product was isolated as a colorless oil from a silica column eluted by EtOAc/hexanes (1:10) in 81% yield (95 mg); ^{1}H NMR (400 MHz, $CDCl_3$): δ 7.35 (d, J = 8.4 Hz, 2H), 7.02 (d, J = 8.4 Hz, 2H), 5.91–5.83 (m, 1H), 5.81–5.70 (m, 1H), 5.17–5.06 (m, 2H), 5.03–4.94 (m, 2H), 4.44 (dt, J = 5.8, 1.4 Hz, 2H), 3.77 (q, J = 7.5, 1H), 2.70 (dd, J = 15.3, 7.6 Hz, 1H), 2.62 (dd, J = 15.3, 7.6 Hz, 1H); ^{13}C NMR (100 MHz, $CDCl_3$): δ 171.3, 141.3, 139.3, 131.6, 131.3, 129.3, 127.9, 127.5, 118.3, 115.3, 65.2, 44.9, 39.9.

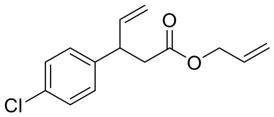

Allyl 3-(4-chlorophenyl)-4-pentenoate (3c)

The product was isolated as a colorless oil from a silica column eluted by EtOAc/hexanes (1:10) in 73% yield (70 mg); ^{1}H NMR (400 MHz, $CDCl_3$): δ 7.20–7.18 (m, 2H), 7.08–7.06 (m, 2H), 5.92–5.83 (m, 1H), 5.882–5.70 (m, 2H), 5.20–5.08 (m, 2H), 5.07–4.93 (m, 2H), 4.45 (dt, J = 5.7, 1.4 Hz, 2H), 3.86–3.73 (m, 1H), 2.75–2.65 (m, 1H), 2.65–2.53 (m, 1H); ^{13}C NMR (100 MHz, $CDCl_3$): δ 171.2, 140.8, 139.7, 131.9, 128.9, 128.6, 128.4, 127.2, 118.3, 115.2, 65.2, 44.8, 40.0.

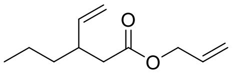

Allyl 3-phenyl-4-pentenoate (3d)

The product was isolated as a colorless oil from a silica column eluted by EtOAc/hexanes (1:10) in 58% yield (42 mg); ^{1}H NMR (400 MHz, $CDCl_3$): δ 5.88–5.76 (m, 1H), 5.61–5.52 (m, 1H), 5.26–5.12 (m, 2H), 4.99–4.86 (m, 2H), 4.49 (dt, J = 5.7, 1.5 Hz, 2H), 2.48–2.45 (m, 1H), 2.38–2.22 (m, 2H), 1.32–1.20 (m, 4H), 0.86–0.79 (m, 3H); ^{13}C NMR (100 MHz, $CDCl_3$): δ 172.2, 141.1, 132.3, 118.1, 114.9, 64.9, 40.2, 40.0, 36.6, 20.0, 13.9.

Prenyl 3-phenyl-4-pentenoate

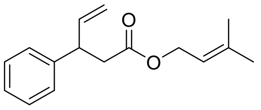

The product was isolated as a colorless oil from a silica column eluted by EtOAc/hexanes (1:10) in 64% yield (15.6 mg) as a colorless oil; ^{1}H NMR (400 MHz, $CDCl_3$): δ 7.32–7.18 (m, 5H), 6.2–5.94 (m, 1H), 5.27–5.26 (m, 1H), 5.13–5.03 (m, 2H), 4.51 (d, J = 7.3 Hz, 2H), 3.87 (d, J = 7.5 Hz, 1H), 2.81–2.65 (m, 2H), 1.73 (s, 3H), 1.66

(s, 3H); ^{13}C NMR (100 MHz, $CDCl_3$): δ 171.8, 142.7, 140.3, 131.1, 128.5, 127.6, 126.6, 118.5, 114.7, 61.3, 45.6, 40.3, 25.5, 24.7. HRMS (EI+, *m/z*): calcd. for $C_{16}H_{20}O_2$ $[M]^+$: 244.1463, found 244.1468.

Cinnamyl 3-phenyl-4-pentenoate

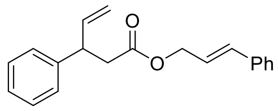

The product was isolated as a colorless oil from a silica column eluted by EtOAc/hexanes (1:10) in 82% yield (2.39 g) as a colorless oil; ^{1}H NMR (400 MHz, $CDCl_3$): *δ* 7.33–7.09 (m, 10H), 6.50 (d, *J* = 15.9, 1H), 6.09 (dt, *J* = 15.9, 6.4 Hz, 1H), 5.92 (ddd, *J* = 17.2, 10.1, 7.0 Hz, 1H), 5.07–4.94 (m, 2H), 4.60 (dd, *J* = 6.5, 1.4 Hz, 2H), 3.82 (q, *J* = 7.5 Hz, 1H), 2.80–2.61 (m, 2H); ^{13}C NMR (100 MHz, $CDCl_3$): δ 171.6, 142.3, 140.2, 136.2, 134.1, 134.0, 128.6, 128.5, 128.0, 127.6, 126.7, 126.6, 126.5, 123.2, 114.9, 65.0, 45.6, 40.3. HRMS (EI+, *m/z*): calcd. for $C_{20}H_{20}O_2$ $[M]^+$: 292.1463, found 292.1466.

4-Phenyl-3,4-dihydro-2*H*-pyran-2-one (4a)

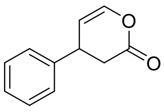

The product was isolated as a colorless oil from a silica column eluted by EtOAc/hexanes (1:9) in 96% yield (66 mg); ^{1}H NMR (400 MHz, $CDCl_3$): *δ* 7.31–7.20 (m, 3H), 7.17–7.12 (m, 2H), 6.61 (dd, *J* = 6.0, 1.8 Hz, 1H), 5.37 (dd, *J* = 6.0, 4.0 Hz, 1H), 3.79–3.69 (m, 1H), 2.91 (dd, *J* = 15.9, 6.5 Hz, 1H), 2.67 (dd, *J* = 16.0, 8.3 Hz, 1H).; ^{13}C{H} NMR (100 MHz, $CDCl_3$): δ 167.3, 141.6, 141.2, 129.1, 127.5, 126.8, 109.4, 37.3, 36.7; HRMS (ESI+, *m/z*): calcd. for $C_{11}H_{11}O_2$ $[M+H]^+$: 175.0759, found 175.0768. The ^{1}H and ^{13}C NMR data were in accordance with those reported in the literature.[3]

4-(4-Bromophenyl)-3,4-dihydro-2*H*-pyran-2-one (4b)

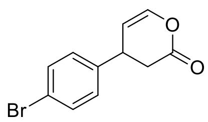

The product was isolated as a colorless oil from a silica column eluted by EtOAc/hexanes (1:9) in 95% yield (96 mg); ^{1}H NMR (200 MHz, $CDCl_3$): *δ* 7.52–7.34 (m, 2H), 7.09–6.94 (m, 2H), 6.61 (dd, *J* = 6.0, 1.7 Hz, 1H), 5.33 (dd, *J* = 6.0, 4.1 Hz, 1H), 3.78–3.64 (m, 1H), 2.89 (dd, *J* = 15.9, 6.9 Hz, 1H), 2.61 (dd, *J* = 15.9, 8.0 Hz, 1H).; ^{13}C{H} NMR (50 MHz, $CDCl_3$): δ 169.2, 142.5, 142.0, 132.2, 125.6, 125.2, 121.4, 108.7, 37.1, 36.2; HRMS (EI+, *m/z*): calcd. for $C_{11}H_9BrO_2$ $[M]^+$: 251.9786, found 251.9741.

4-(4-Chlorophenyl)-3,4-dihydro-2*H*-pyran-2-one (4c)

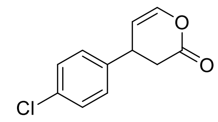

The product was isolated as a colorless oil from a silica column eluted by EtOAc/hexanes (1:9) in 87% yield (72 mg); ^{1}H NMR (200 MHz, $CDCl_3$): *δ* 7.33–7.19 (m, 2H), 7.16–6.97 (m, 2H), 6.62 (dd, *J* = 6.0, 1.8 Hz, 1H), 5.34 (dd, *J* = 6.1, 4.1 Hz, 1H), 3.77–3.68 (m, 1H), 2.90 (dd, *J* = 16.0, 7.0 Hz, 1H), 2.62 (dd, *J* = 16.0, 8.0 Hz, 1H).; ^{13}C{H} NMR (50 MHz, $CDCl_3$): δ 171.2, 141.9, 141.5, 129.2, 128.2, 108.8, 37.2, 36.1; HRMS (EI+, *m/z*): calcd. for $C_{11}H_9ClO_2$ $[M]^+$: 208.0291, found 208.0287.

4-Propyl-3,4-dihydro-2*H*-pyran-2-one (4d)

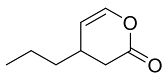

The product was isolated as a colorless oil from a silica column eluted by EtOAc/hexanes (1:9) in 83% yield (46 mg); ^{1}H NMR (400 MHz, $CDCl_3$): *δ* 6.40 (dd, *J* = 6.0, 1.6 Hz, 1H), 5.18 (dd, *J* = 5.9, 4.0 Hz, 1H), 2.69–2.59 (m, 1H), 2.51–2.42 (m, 1H), 2.34 (dd, *J* = 15.5, 8.0 Hz, 1H), 1.38–1.27 (m, 4H), 0.88–0.81 (m, 3H); ^{13}C{H} NMR (100 MHz, $CDCl_3$) δ 168.5, 140.6, 110.4, 36.8, 34.9, 30.2, 19.5, 13.9; HRMS (ESI+, *m/z*): calcd. for $C_8H_{12}O_2$ $[M+H]^+$: 140.0837, found 140.0849. The ^{1}H and ^{13}C NMR data were in accordance with those reported in the literature.[3]

4-Phenyl-3,4-dihydrooxepin-2*(7H)*-one (5a)

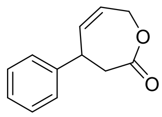

The product was isolated from a silica column eluted by EtOAc/hexanes (2:8) in 75% yield (56 mg) as white crystals; ^{1}H NMR (400 MHz, $CDCl_3$): *δ* 7.40–7.25 (m, 5H), 6.08–5.99 (m, 1H), 5.95–5.85 (m, 1H), 4.99–4.93 (m, 1H), 4.71–4.65 (m, 1H), 3.95–3.86 (m, 1H), 3.18 (dd, *J* = 13.0, 10.2 Hz, 1H), 3.04 (ddd, *J* = 13.1, 4.2, 1.0 Hz, 1H); ^{13}C NMR (100 MHz, $CDCl_3$): δ 172.8, 141.8, 135.5, 128.9, 127.5, 127.4, 124.5, 63.9, 42.3, 39.7. The ^{1}H and ^{13}C NMR data were in accordance with those reported in the literature.[2]

4-(4-Bromophenyl)-3,4-dihydrooxepin-2*(7H)*-one (5b)

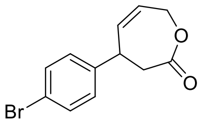

The product was isolated from a silica column eluted by EtOAc/hexanes (2:8) in 62% yield (32 mg) as white crystals; ^{1}H NMR (400 MHz, $CDCl_3$): *δ* 7.40 (d, *J* = 8.4 Hz, 2H), 7.03 (d, *J* = 8.4 Hz, 2H), 5.98–5.92 (m, 1H), 5.82–5.76 (m, 1H), 4.84–4.71 (m, 1H), 4.68–4.56 (m, 1H), 3.83–3.75 (m, 1H), 3.07–2.88 (m, 2H); ^{13}C NMR (100 MHz, $CDCl_3$): δ 168.1, 146.1, 134.7, 132.1, 129.2, 125.1, 63.8, 41.7, 39.5. HRMS (EI+, *m/z*): calcd. for $C_{12}H_{11}BrO_2$ $[M]^+$: 267.1185, found 267.1187.

4-(4-Chlorophenyl)-3,4-dihydrooxepin-2*(7H)*-one (5c)

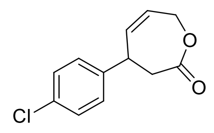

The product was isolated from a silica column eluted by EtOAc/hexanes (2:8) in 58% yield (25 mg) as white crystals; ^{1}H NMR (400 MHz, $CDCl_3$): *δ* 7.25–7.20 (m, 2H), 7.11–7.08 (m, 2H), 5.98–5.94 (m, 1H), 5.79–5.75 (m, 1H), 4.84–4.79 (m, 1H), 4.64–4.56 (m, 1H), 3.75–3.71 (m, 1H), 3.01 –2.97 (m, 2H); ^{13}C NMR (100 MHz, $CDCl_3$): δ 171.1, 142.3, 134.6, 131.6, 129.8, 128.1, 63.5, 43.5, 39.9. HRMS (EI+, *m/z*): calcd. for $C_{12}H_{11}ClO_2$ $[M]^+$: 222.6679, found 222.6680.

4-Propyl-3,4-dihydrooxepin-2*(7H)*-one (5d)

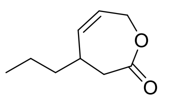

The product was isolated from a silica column eluted by EtOAc/hexanes (2:8) in 43% yield (13 mg) as semi-solid; ^{1}H NMR (400 MHz, $CDCl_3$): 5.75–5.65 (m, 2H), 4.64–4.49 (m, 2H), 2.84–2.74 (m, 1H), 2.71–2.69 (m, 1H), 2.52–2.48 (m, 1H), 1.40–1.34 (m, 4H), 0.88–0.84 (m, 3H); ^{13}C NMR (100 MHz, $CDCl_3$): δ 172.3, 141.1, 132.4, 65.0, 40.1, 36.7, 20.1, 14.0. HRMS (EI+, *m/z*): calcd. for $C_9H_{14}O_2$ $[M]^+$: 154.2066, found 154.2068.

N-allyl-N-benzyl-3-phenyl-4-pentenamide (7a)

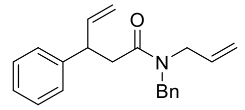

The product was isolated from a silica column eluted by EtOAc/hexanes (1:9) in 85% yield (778 mg) as white crystals; ^{1}H NMR (400 MHz, $CDCl_3$): *δ* 7.39–7.13 (m, 8H), 7.07 (dd, *J* = 7.4, 2.2 Hz, 2H), 6.19–5.92 (m, 1H), 5.68–5.65 (m, 1H), 5.22–4.91 (m, 4H), 4.72–4.34 (m, 2H), 4.10–4.06 (m, 1H), 3.98–3.93 (m, 1H), 3.76–3.71 (m, 1H), 2.79–2.75 (m, 2H); ^{13}C NMR (100 MHz, $CDCl_3$): δ 171.5, 143.1, 140.9, 140.7, 137.4, 136.7, 132.9, 132.6, 128.8, 128.6, 128.5, 128.4, 128.0, 127.9, 127.8, 127.5, 127.2, 126.5, 126.3, 117.3, 116.7, 114.6, 50.0, 49.1, 48.3, 48.1, 45.7, 45.6, 38.8, 38.5. The ^{1}H and ^{13}C NMR data were in accordance with those reported in the literature.[1]

N-allyl-N-benzyl-3-(4-bromophenyl)-4-pentenamide (7b)

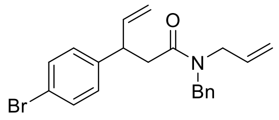

The product was isolated from a silica column eluted by EtOAc/hexanes (1:9) in 78% yield (59 mg) as semi-solid; ^{1}H NMR (400 MHz, $CDCl_3$): *δ* 7.37–7.27 (m, 2H), 7.23–7.13 (m, 3H), 7.10–6.89 (m, 4H), 5.98 –5.80 (m, 1H), 5.69–5.48 (m, 1H), 5.21–4.86 (m, 4H), 4.61–4.30 (m, 1H), 4.06 –3.91 (m, 1H), 3.91–3.73 (m, 1H), 3.72 –3.62 (m, 1H), 2.66 (dd, *J* = 7.2, 3.8 Hz, 2H); ^{13}C NMR (100 MHz, $CDCl_3$): δ 171.1, 142.0, 140.3, 137.3, 136.5, 132.8, 132.5, 131.5, 129.7, 129.6, 128.8, 128.4, 127.9, 127,5, 127.2, 126.2, 120.3, 116.8, 115.0, 50.0, 49.1, 48.3, 45.0, 38.2.

N-allyl-N-benzyl-3-(4-chlorophenyl)-4-pentenamide (7c)

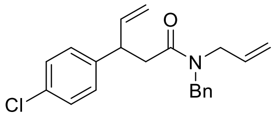

The product was isolated from a silica column eluted by EtOAc/hexanes (1:9) in 73% yield (50 mg) as semi-solid; ^{1}H NMR (400 MHz, $CDCl_3$): *δ* 7.29–7.25 (m, 2H), 7.22 – 7.19 (m, 2H), 7.17–7.01 (m, 4H), 7.01–6.95 (m, 1H), 5.98–5.86 (m, 1H), 5.72–5.56 (m, 1H), 5.17–4.90 (m, 4H), 4.53–4.33 (m, 2H), 4.06–3.96 (m, 1H), 3.79–3.72 (m, 1H), 3.71–3.63 (m, 1H), 2.71–2.65 (m, 2H); ^{13}C NMR (100 MHz, $CDCl_3$): δ 171.2, 141.5, 140.5, 140.4, 137.3, 132.8, 132.5, 129.3, 128.8, 128.6, 128.5, 128.3, 128.2, 127.9, 126.2, 116.7, 114.9, 50.0, 49.1, 48.4, 44.9, 38.2.

N-allyl-N-benzyl-3-propyl-4-pentenamide (7d)

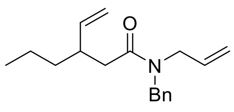

The product was isolated from a silica column eluted by EtOAc/hexanes (1:9) in 57% yield (31 mg) as semi-solid; ^{1}H NMR (400 MHz, $CDCl_3$): *δ* 7.29–7.07 (m, 5H), 5.71–5.49 (m, 2H), 5.15–5.02 (m, 2H), 4.99–4.89 (m, 2H), 4.57–4.37 (m, 2H), 3.99–3.85 (m, 1H), 3.79–3.68 (m, 1H), 2.32 –2.67 (m, 2H), 1.35–1.15 (m, 4H), 0.85–0.77 (m, 3H); ^{13}C NMR (50 MHz, $CDCl_3$): δ 172.1, 141.7, 137.6, 133.1, 132.7, 128.8, 128.4, 128.1, 127.2, 126.3, 116.7, 114.7, 50.1, 49.5, 48.1, 40.3, 38.4, 36.8, 20.2, 13.9.

1-Benzyl-4-phenyl-1,3,4,7-tetrahydro-azepin-2-one (8a)

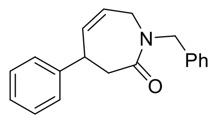

The product was isolated from silica column eluted by EtOAc/hexanes (25:75) with 91% yield (101 mg) as white crystals; ^{1}H NMR (400 MHz, $CDCl_3$): *δ* 7.30–7.11 (m, 10H), 5.77–5.62 (m, 2H), 4.59 (s, 2H), 4.16 (ddt, *J* = 17.7, 4.1, 2.4 Hz, 1H), 3.77 (dt, *J* = 11.6, 3.0 Hz, 1H), 3.51 (ddd, *J* = 17.6, 6.6, 1.6 Hz, 1H), 3.08 (dd, *J* = 13.0, 11.4 Hz, 1H), 2.79–2.70 (m, 1H); ^{13}C NMR (100 MHz, $CDCl_3$): δ 172.8, 143.7, 137.4, 134.8, 128.7, 128.5, 128.0, 127.5, 127.4, 126.9, 124.9, 51.2, 45.2, 42.2, 42.1; HRMS (ESI+, *m/z*): calcd. for $C_{19}H_{20}NO$ $[M+H]^+$: 278.1539, found 278.1536. The ^{1}H and ^{13}C NMR data were in accordance with those reported in the literature.[1]

1-Benzyl-4-(4-bromophenyl)-1,3,4,7-tetrahydro-azepin-2-one (8b)

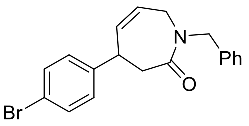

The product was isolated from silica column eluted by EtOAc/hexanes (25:75) with 80% yield (42 mg) as white crystals; ^{1}H NMR (400 MHz, $CDCl_3$): *δ* 7.40–7.31 (m, 2H), 7.26–7.17 (m, 5H), 7.09–7.03 (m, 2H), 5.98–5. 96 (m, 1H), 5.48–5.42 (m, 1H), 4.60 (s, 2H), 3.46–3.41 (m, 1H), 2.78 (d, *J* = 6.7 Hz, 2H), 2.42–2. 34 (m, 1H), 2.16–2.08 (m, 1H); ^{13}C NMR (100 MHz, $CDCl_3$): δ 172.2, 144.1, 131.7, 130.8, 128.6, 128.3, 127.9, 127.4, 50.3, 45.9, 42.4, 33.6; HRMS (EI+, *m/z*): calcd. for $C_{19}H_{18}BrNO$ $[M]^+$: 356.2566, found 356.2569.

1-Benzyl-4-(4-chloroophenyl)-1,3,4,7-tetrahydro-azepin-2-one (8c)

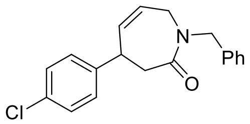

The product was isolated from silica column eluted by EtOAc/hexanes (25:75) with 75% yield (31 mg) as white crystals; ^{1}H NMR (400 MHz, $CDCl_3$): *δ* 7.32–7.25 (m, 7H), 7.19–7.17 (m, 2H), 6.07–6.04 (m, 1 H), 5.55–5.49 (m, 1H), 4.67 (s, 2H), 3.56–3.49 (m, 1H), 2.86 (d, *J* = 6.8 Hz, 2H), 2.49–2.42 (m, 1H), 2.26–2.15 (m, 1H).; ^{13}C NMR (50 MHz, $CDCl_3$): δ 172.3, 143.6, 137.3, 130.7, 128.7, 128.6, 128.0, 127.9, 127.4, 50.3, 45.9, 42.5, 33.6. HRMS (EI+, *m/z*): calcd. for $C_{19}H_{18}ClNO$ $[M]^+$: 311.8060, found 311.8058.

1-Benzyl-4-propyl-1,3,4,7-tetrahydro-azepin-2-one (8d)

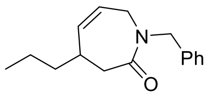

The product was isolated from silica column eluted by EtOAc/hexanes (25:75) with 62% yield (15 mg) as white crystals; ^{1}H NMR (400 MHz, $CDCl_3$): *δ* 7.30–7.14 (m, 5H), 5.61–5.57 (m, 1H), 5.56–5.44 (m, 1H), 4.55 (s, 2H), 3.88–3.74 (m, 1H), 3.61–3.54 (m, 1H), 2.72–2.63 (m, 2H), 2.51–2.38 (m, 1H), 1.41–1.32 (m, 4 H), 0.87–0.81 (m, 3H); ^{13}C NMR (100 MHz, $CDCl_3$): δ 173.7, 137.5, 136.3, 128.4, 128.0, 127.8, 127.2, 123.5, 51.0, 45.4, 38.8, 38.1, 35.2, 19.7, 14.0; HRMS (EI+, *m/z*): calcd. for $C_{16}H_{21}NO$ $[M]^+$: 243.3447, found 243.3450.

4-Phenyltetrahydro-2H-pyran-2-one (9)

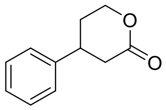

The product was isolated from silica column eluted by EtOAc/hexanes (25:75) with 100% yield (19 mg) as colorless oil; ^{1}H NMR (400 MHz, $CDCl_3$): *δ* 7.30-7.25 (m, 2H), 7.24–7.18 (m, 1H), 7.17–7,07 (m, 2H), 4.56–4.41 (m, 1H), 4.32 (ddd, *J* = 11.4, 10.4 and 3.8 Hz, 1H), 3.17 (tdd, *J* = 10.6, 6.0 and 4.6 Hz, 1H), 2.84 (ddd, *J* = 17.6, 6.0, and 1.6 Hz, 1H), 2.56 (dd, *J* = 17.6 and 10.6 Hz, 1H), 2.16–2.03 (m, 1H), 2.01–1.92 (m, 1H); ^{13}C NMR (100 MHz, $CDCl_3$) δ 170.6, 142.8, 128.9, 127.2, 126.4, 68.6, 37.4, 37.3, 30.3. The ^{1}H and ^{13}C NMR data were in accordance with those reported in the literature.[4]

Methyl 5-oxo-3-phenylpentanoate (10)

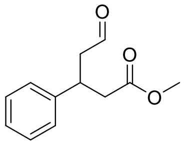

The product was isolated from silica column eluted by EtOAc/hexanes (15:85) with 95% yield (29 mg) as colorless oil; ^{1}H NMR (400 MHz, $CDCl_3$): *δ* 9.60 (t, *J* = 1.8 Hz, 1H), 7.26–7.14 (m, 5H), 3.76–3.61 (m, 1H), 3.54 (s, 3H), 2.85–2.67 (m, 2H), 2.61 (dd, *J* = 7.4, 2.7 Hz, 2H); ^{13}C NMR (100 MHz, $CDCl_3$): δ 201.0, 172.4, 141.6, 129.0, 127.4, 126.8, 51.8, 49.6, 40.9, 36.6. The ^{1}H and ^{13}C NMR data were in accordance with those reported in the literature.[5]

6. ^{1}H NMR and ^{13}C NMR spectra of the products

Vinyl 3-phenyl-4-pentenoate (1a)

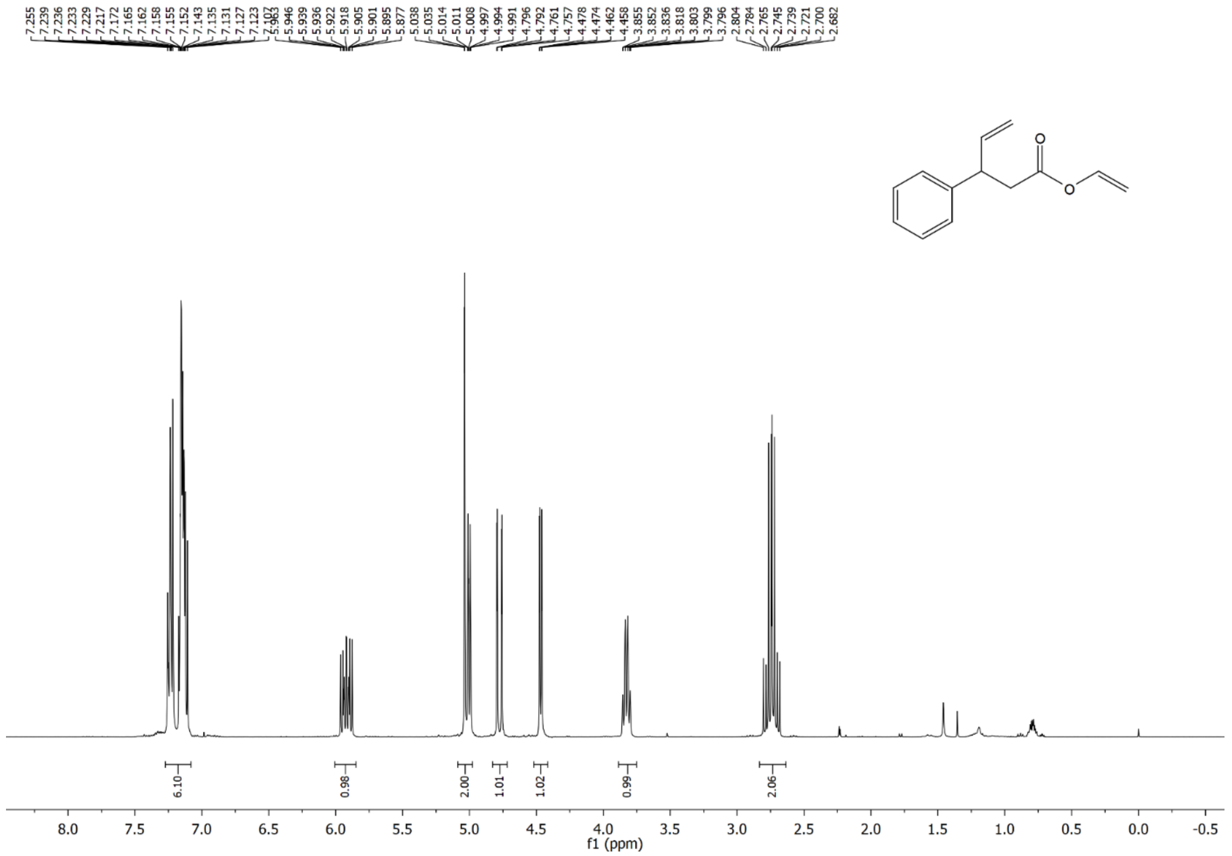

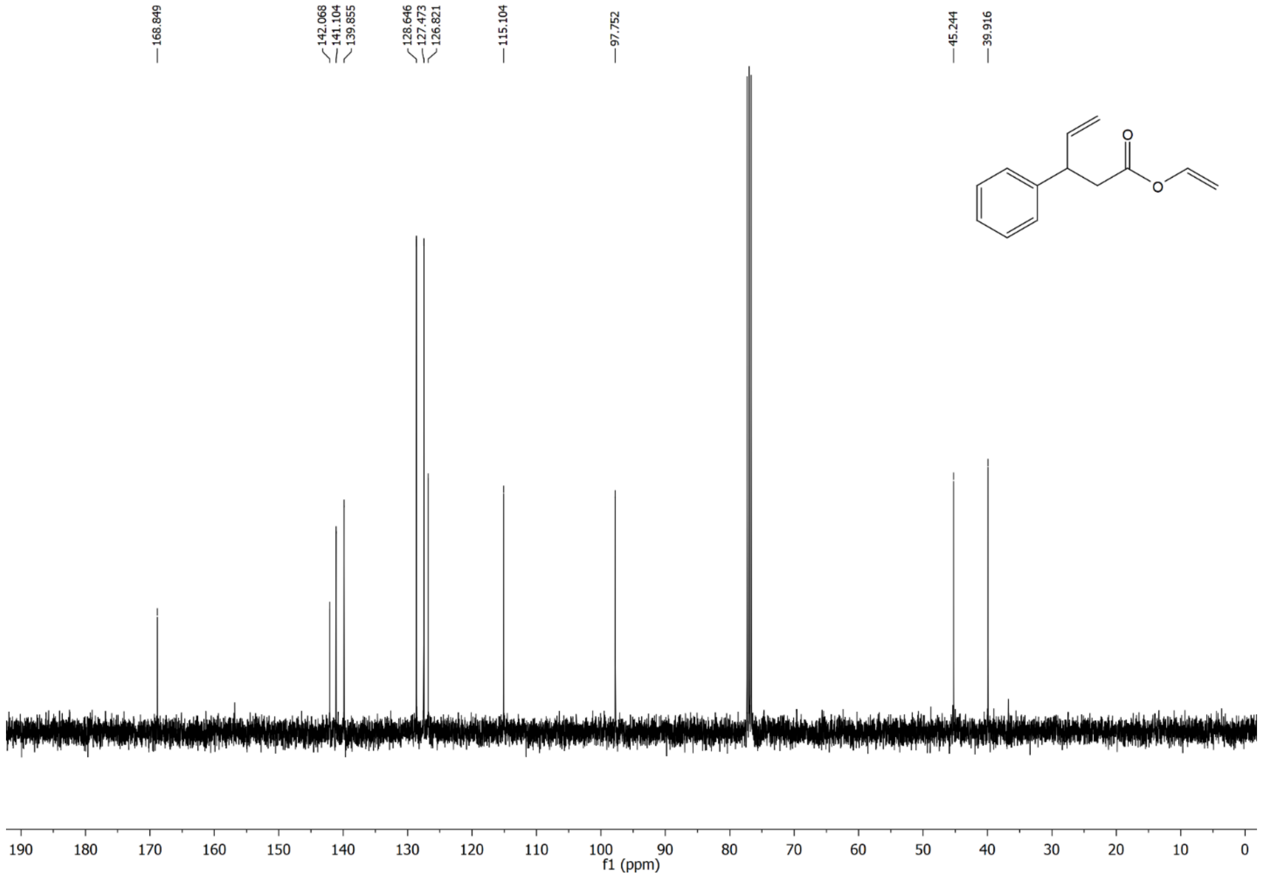

Vinyl 3-(4-bromophenyl)-4-pentenoate (1b)

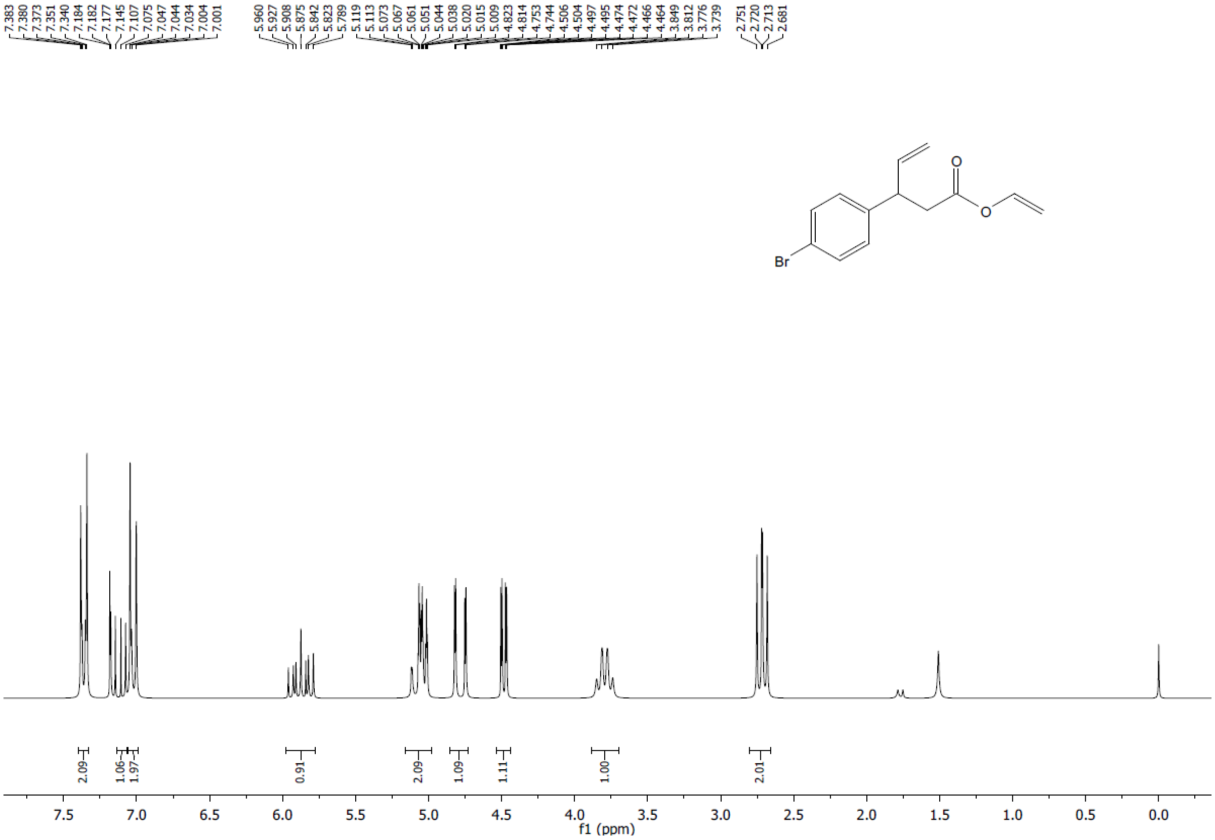

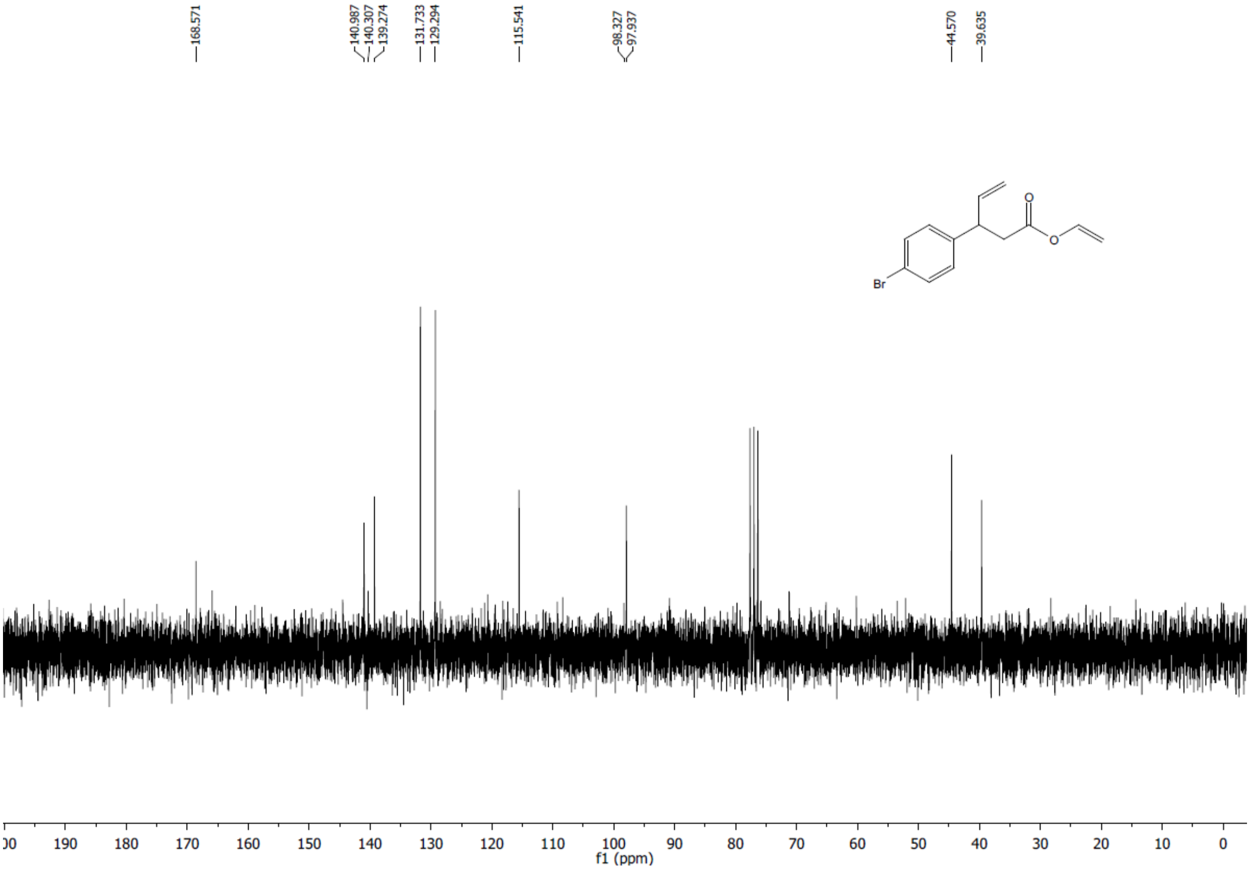

Vinyl 3-(4-chlorophenyl)-4-pentenoate (1c)

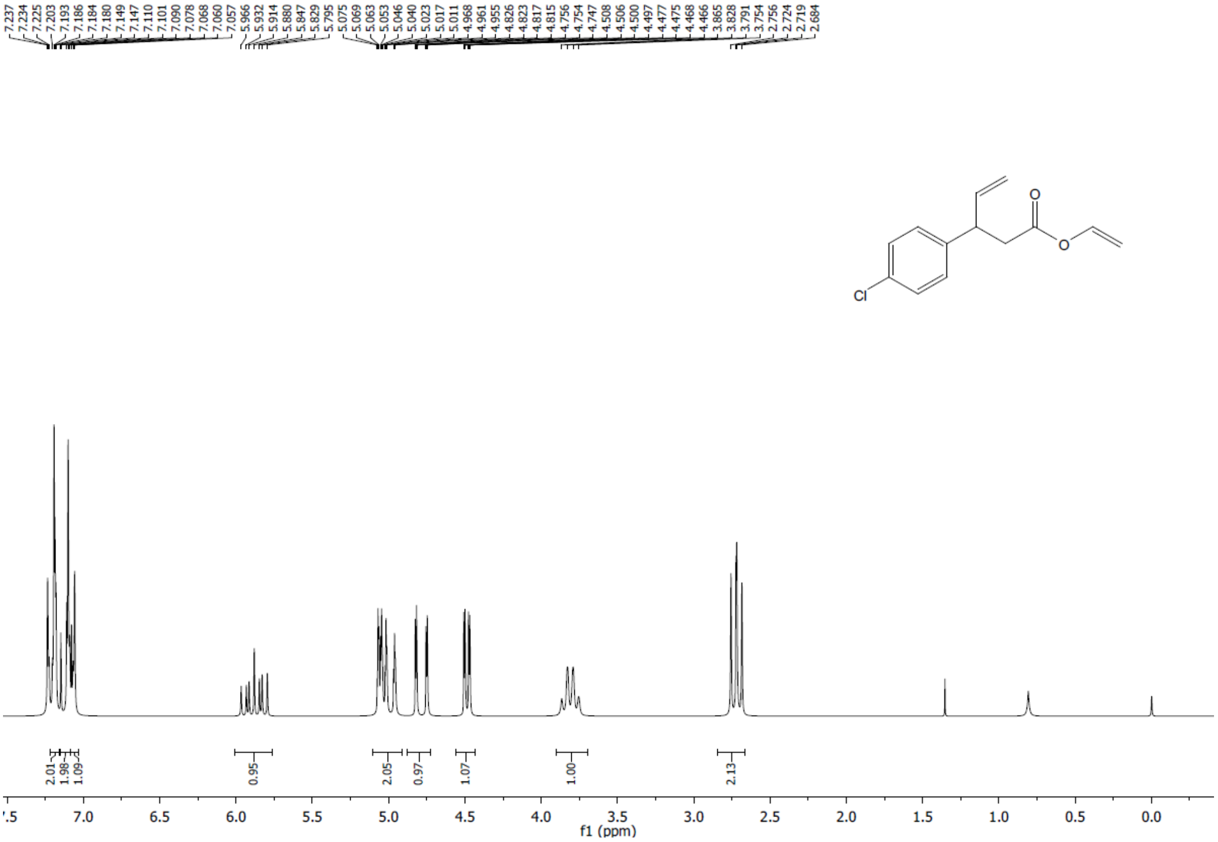

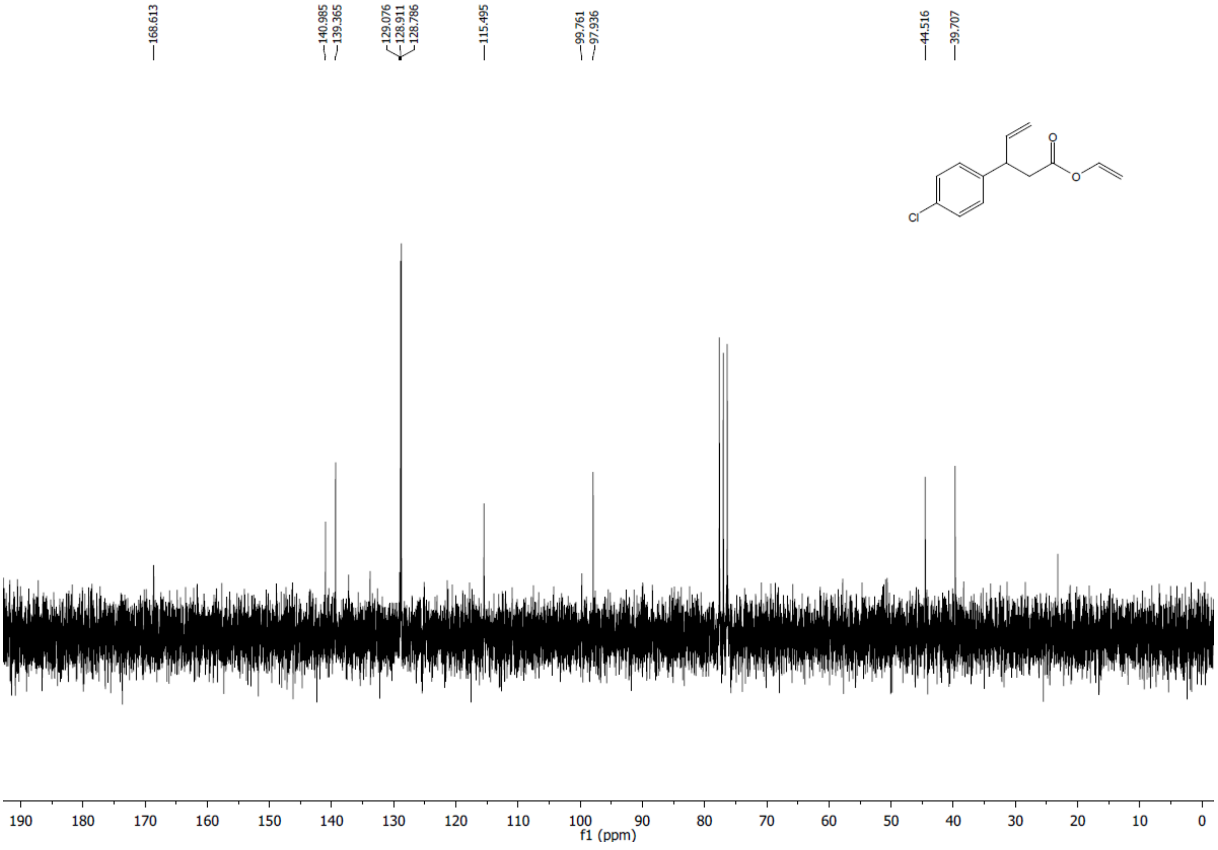

Vinyl 3-propyl-4-pentenoate (1d)

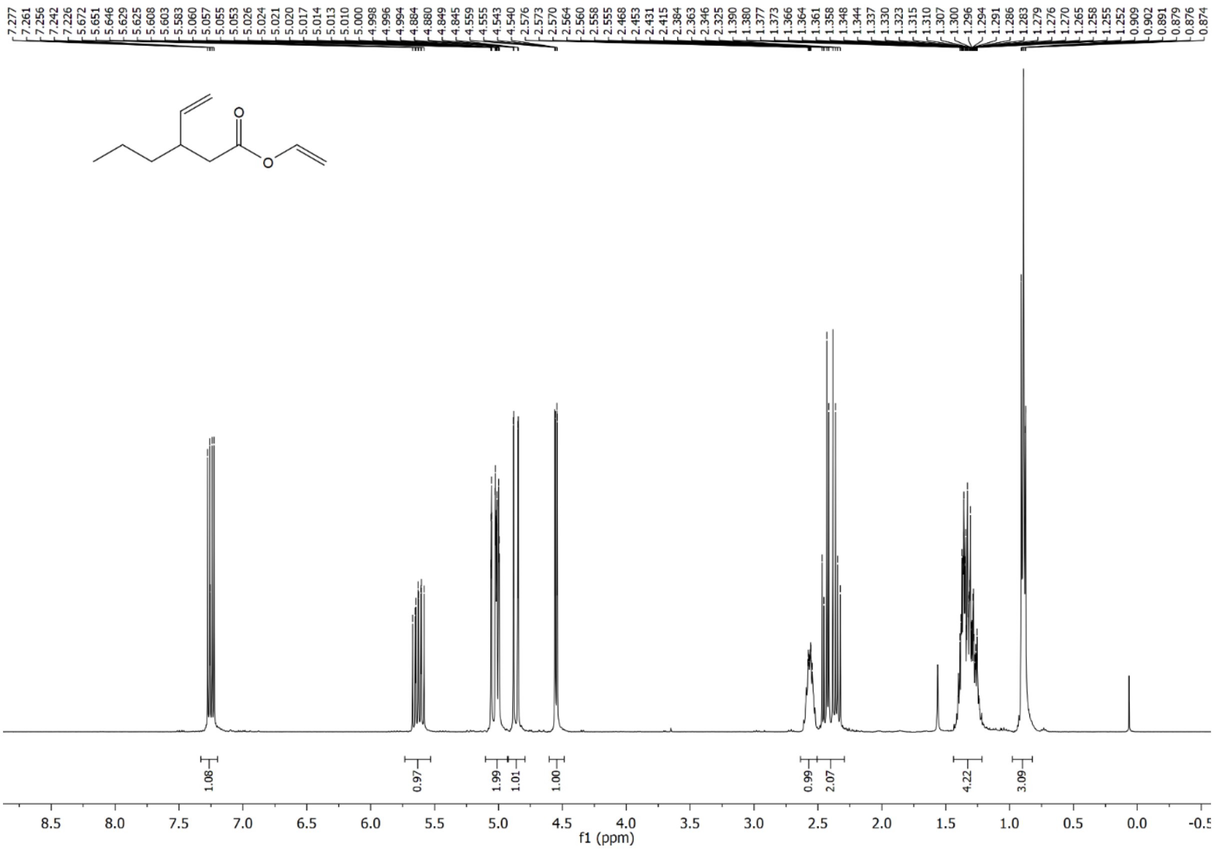

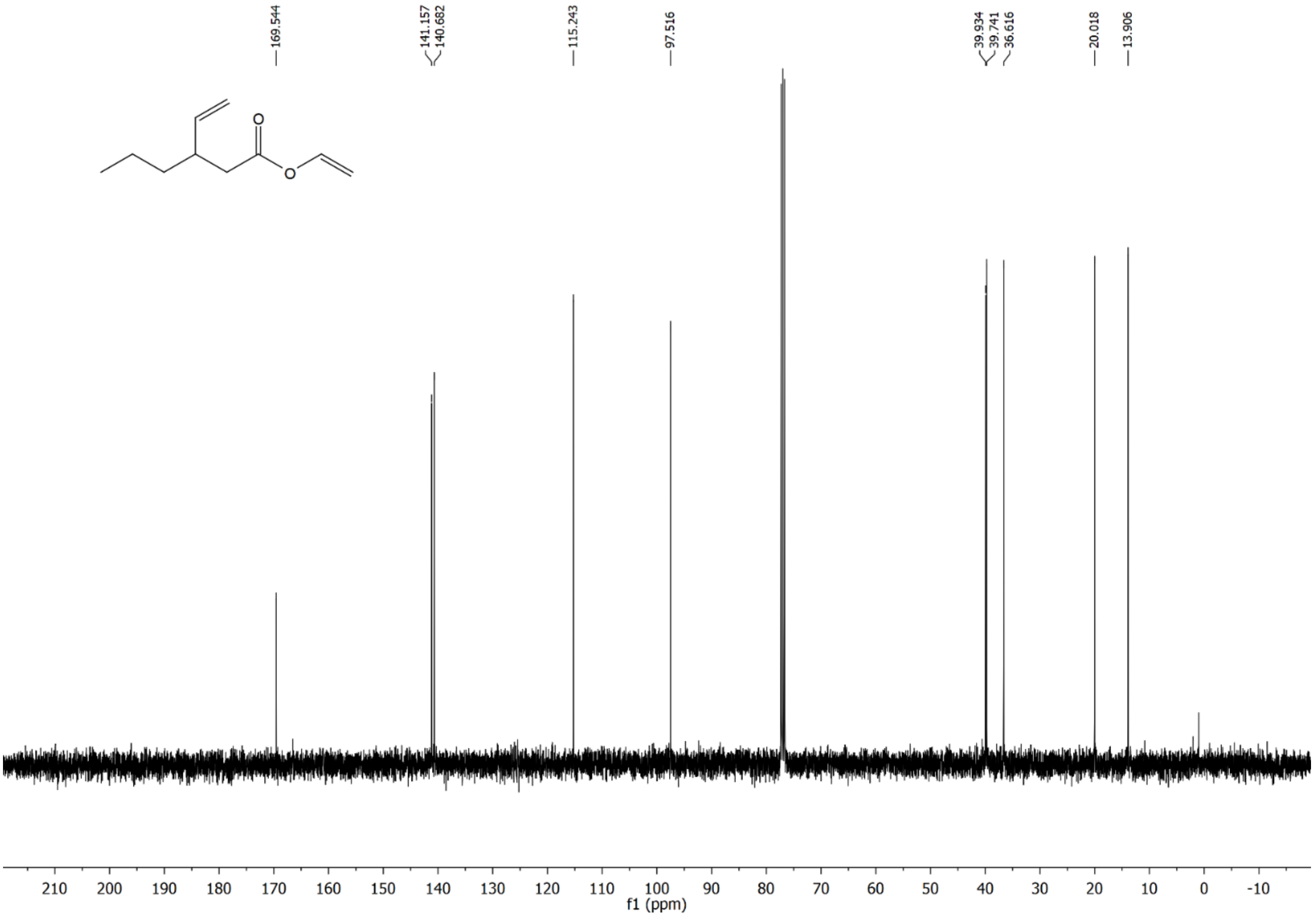

Allyl 3-phenyl-4-pentenoate (3a)

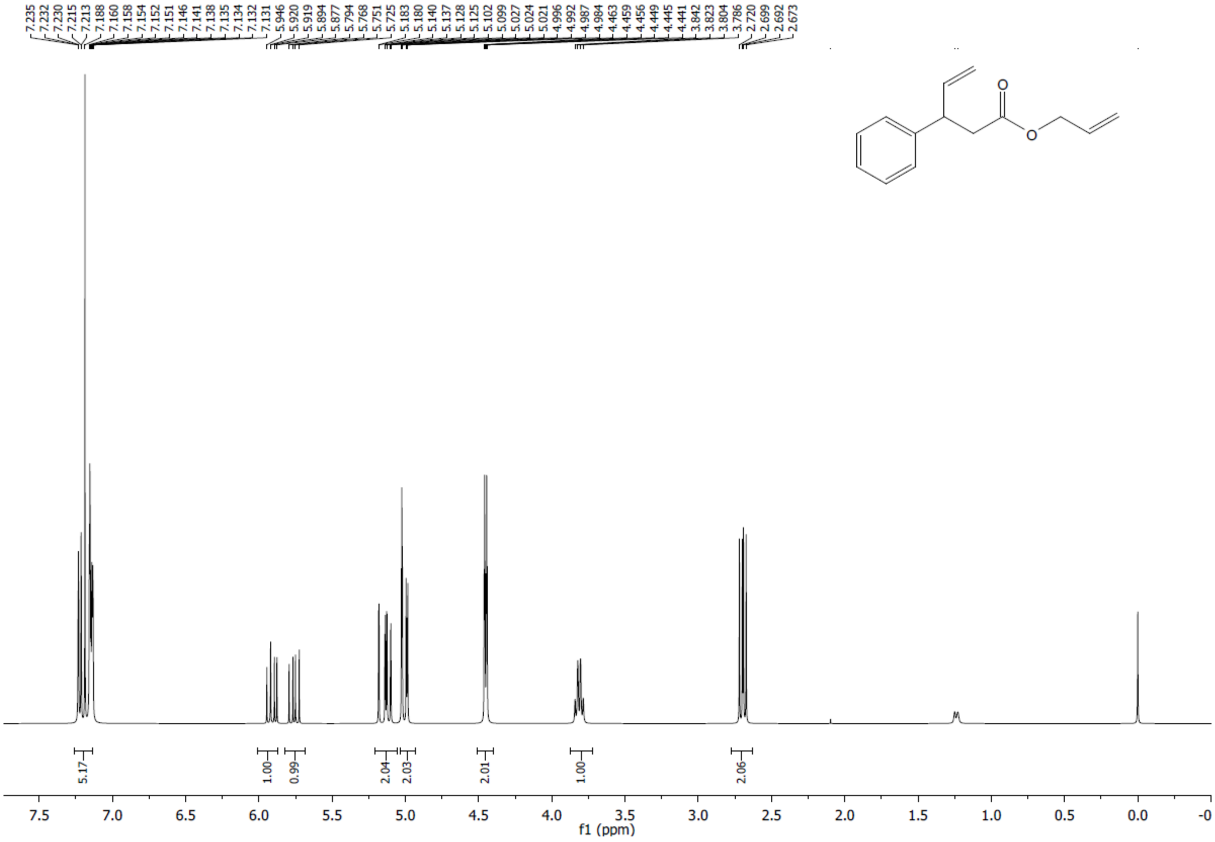

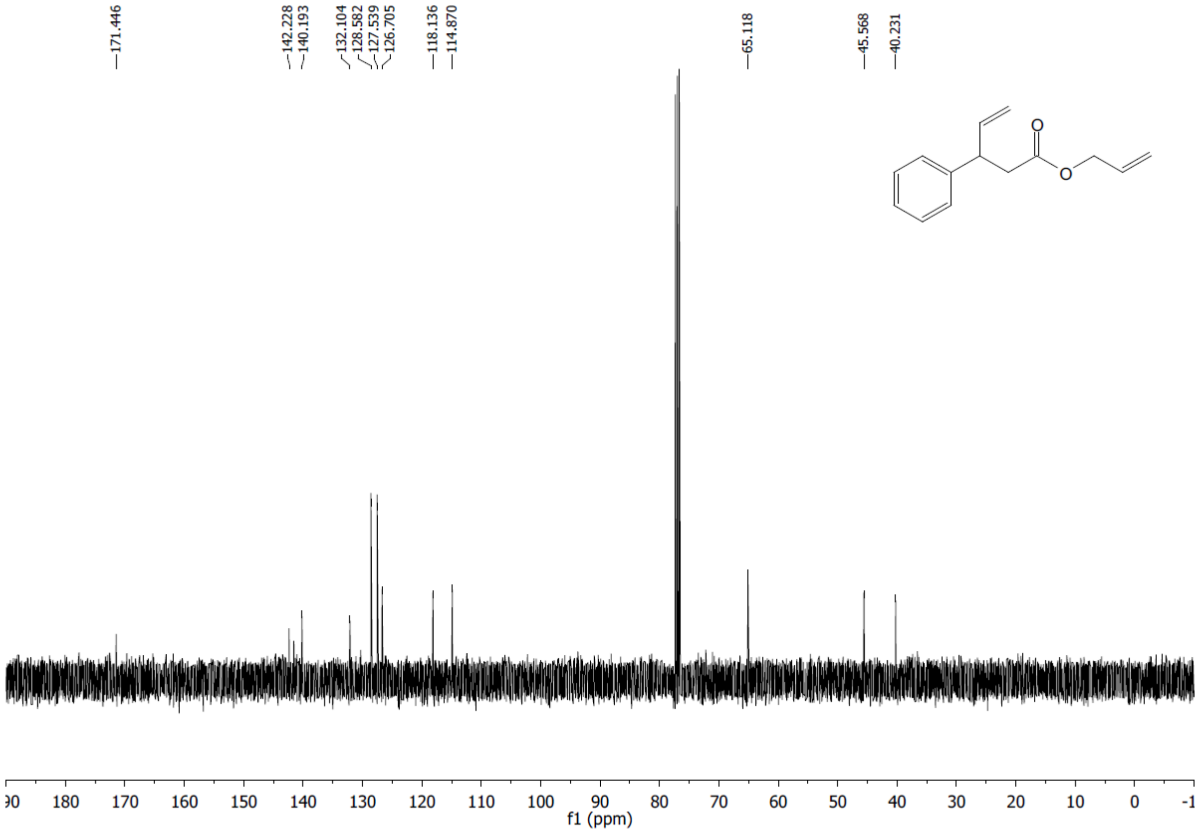

Allyl 3-(4-bromophenyl)-4-pentenoate (3b)

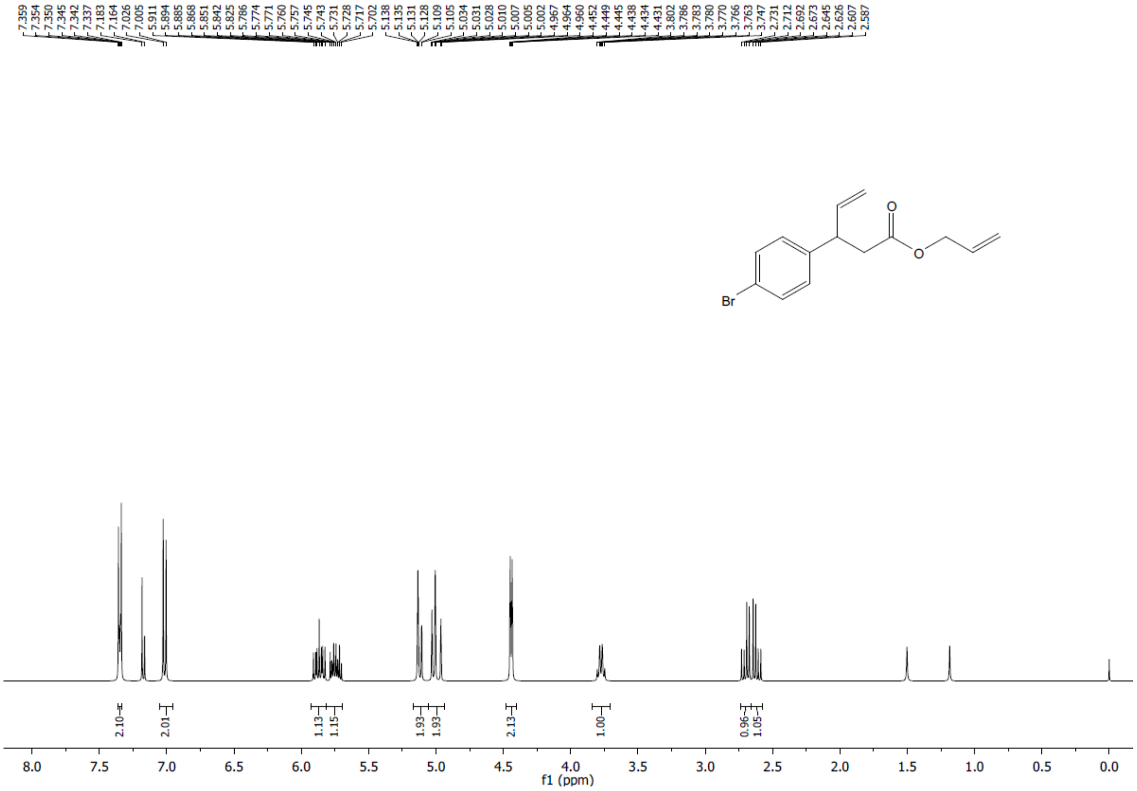

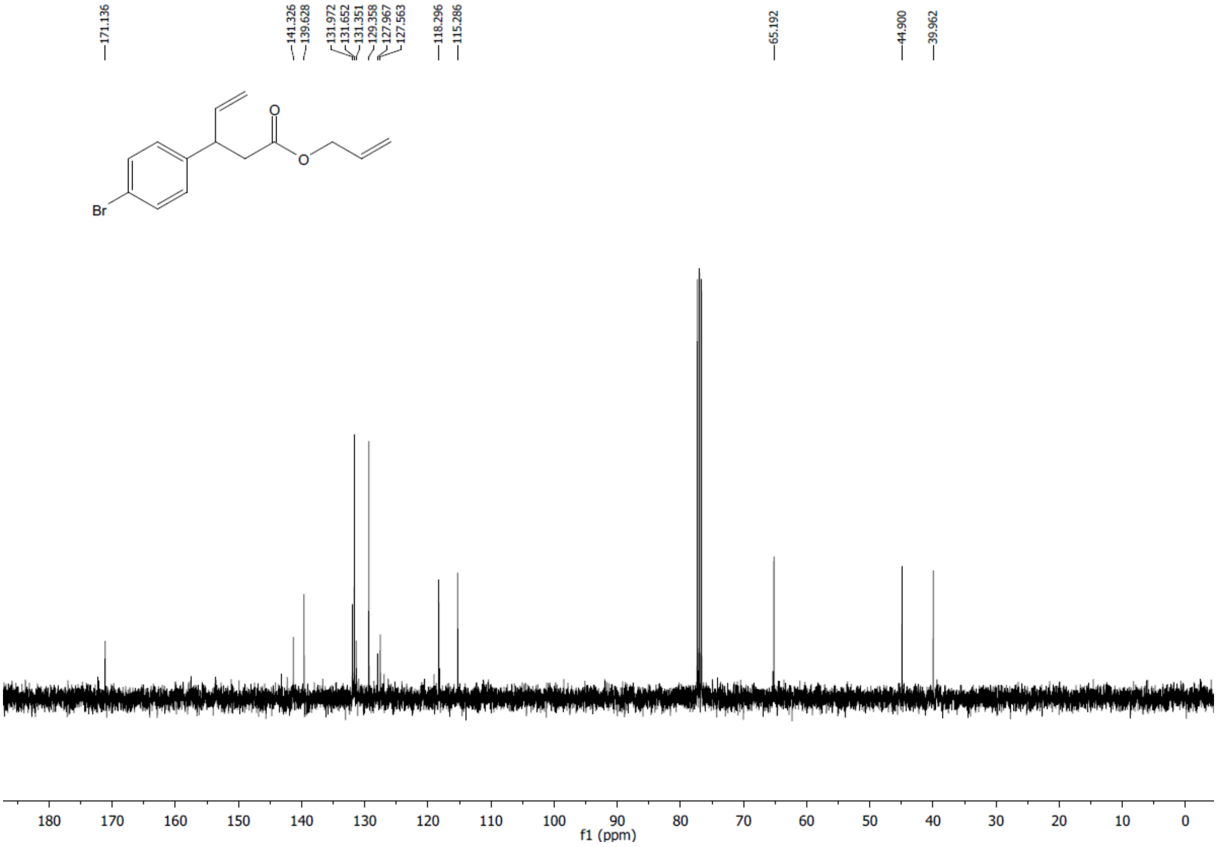

Allyl 3-(4-chlorophenyl)-4-pentenoate (3c)

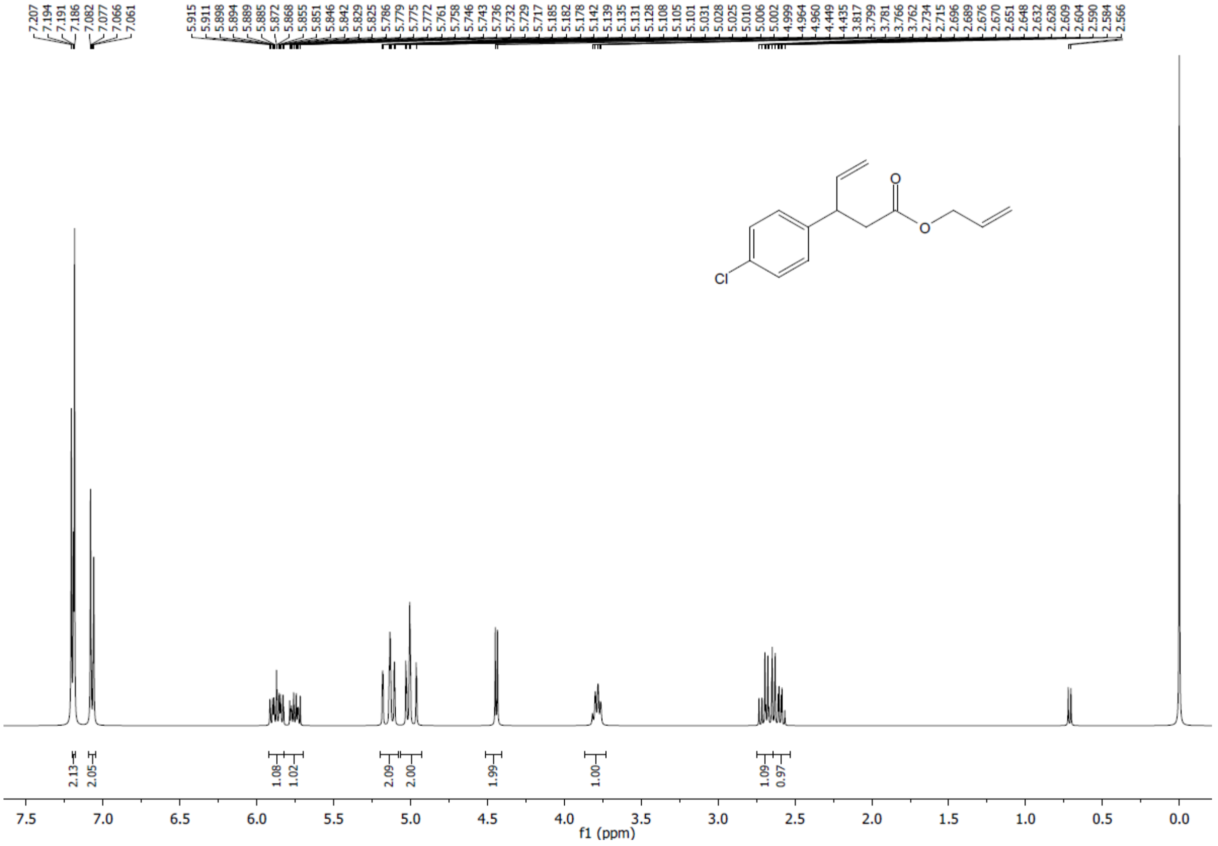

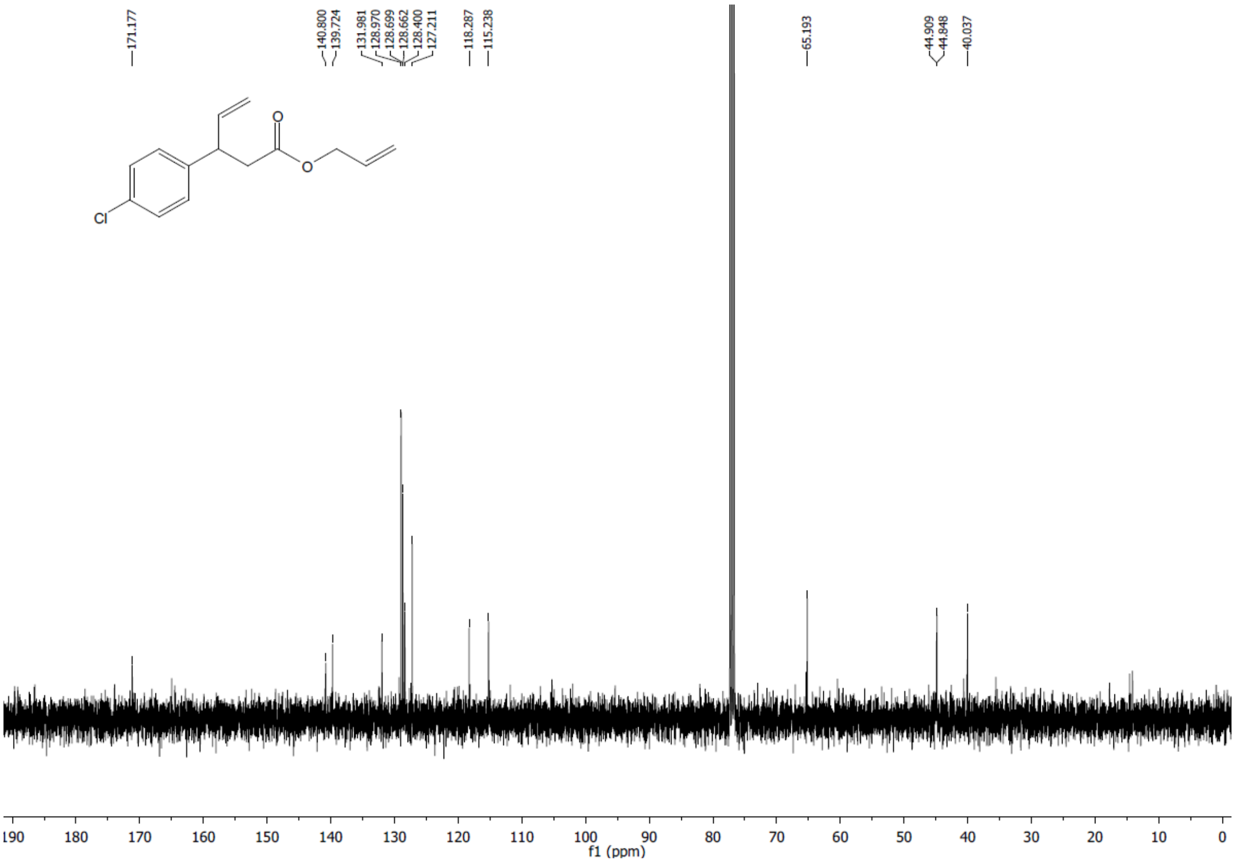

Allyl 3-propyll-4-pentenoate (3d)

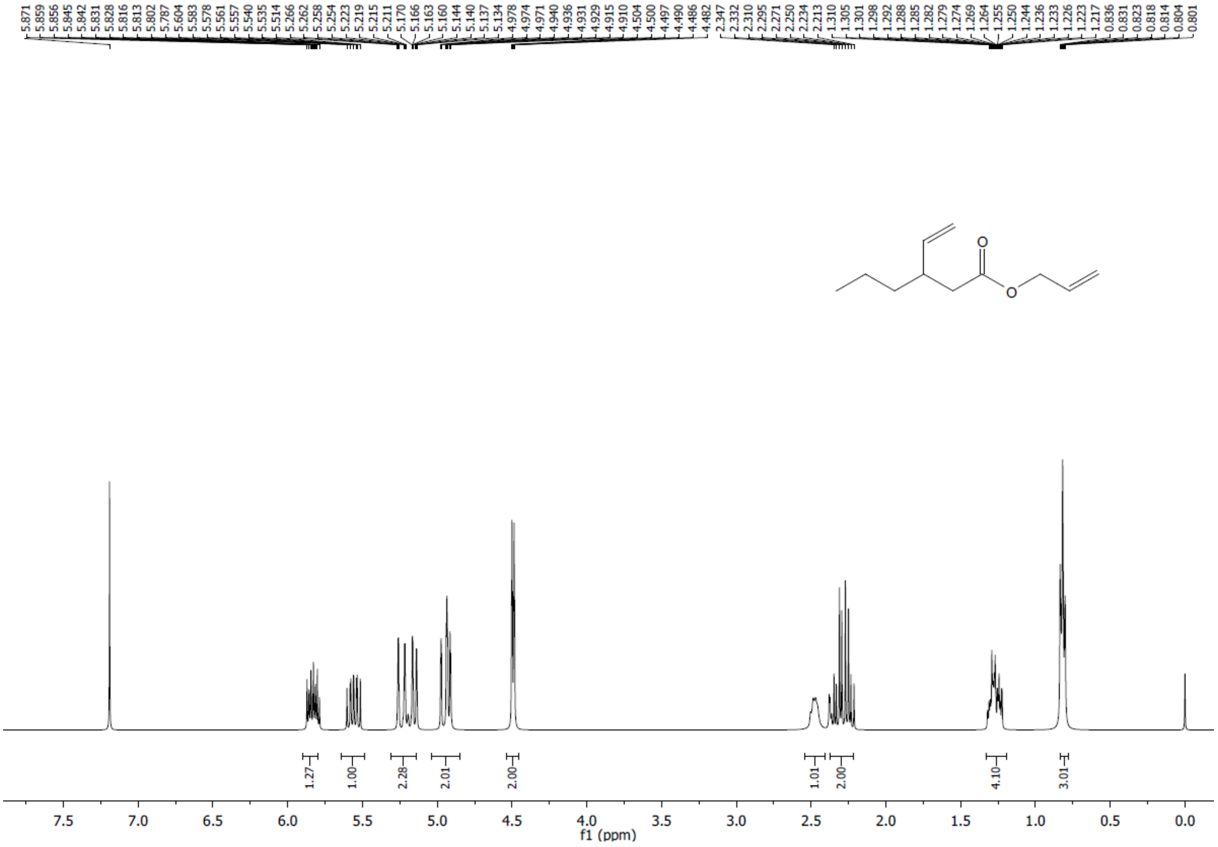

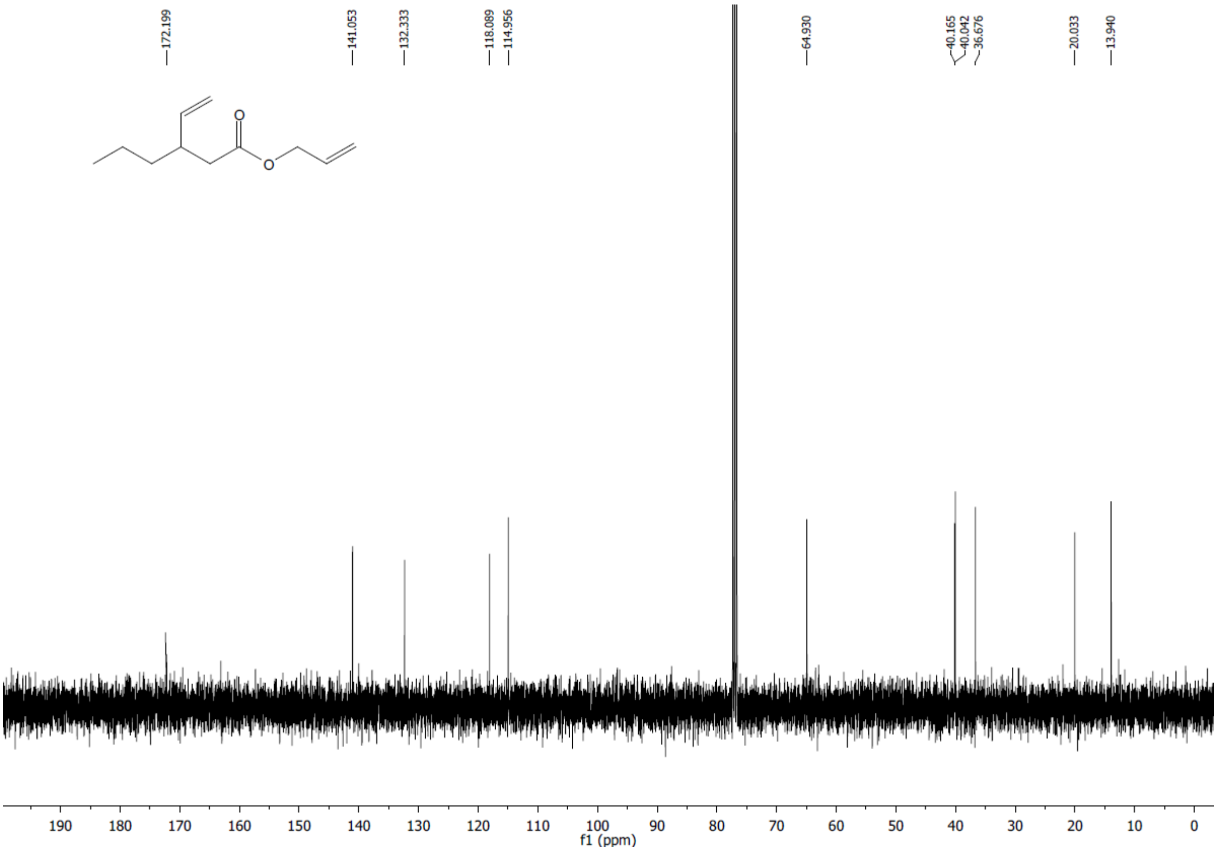

Prenyl 3-phenyl-4-pentenoate

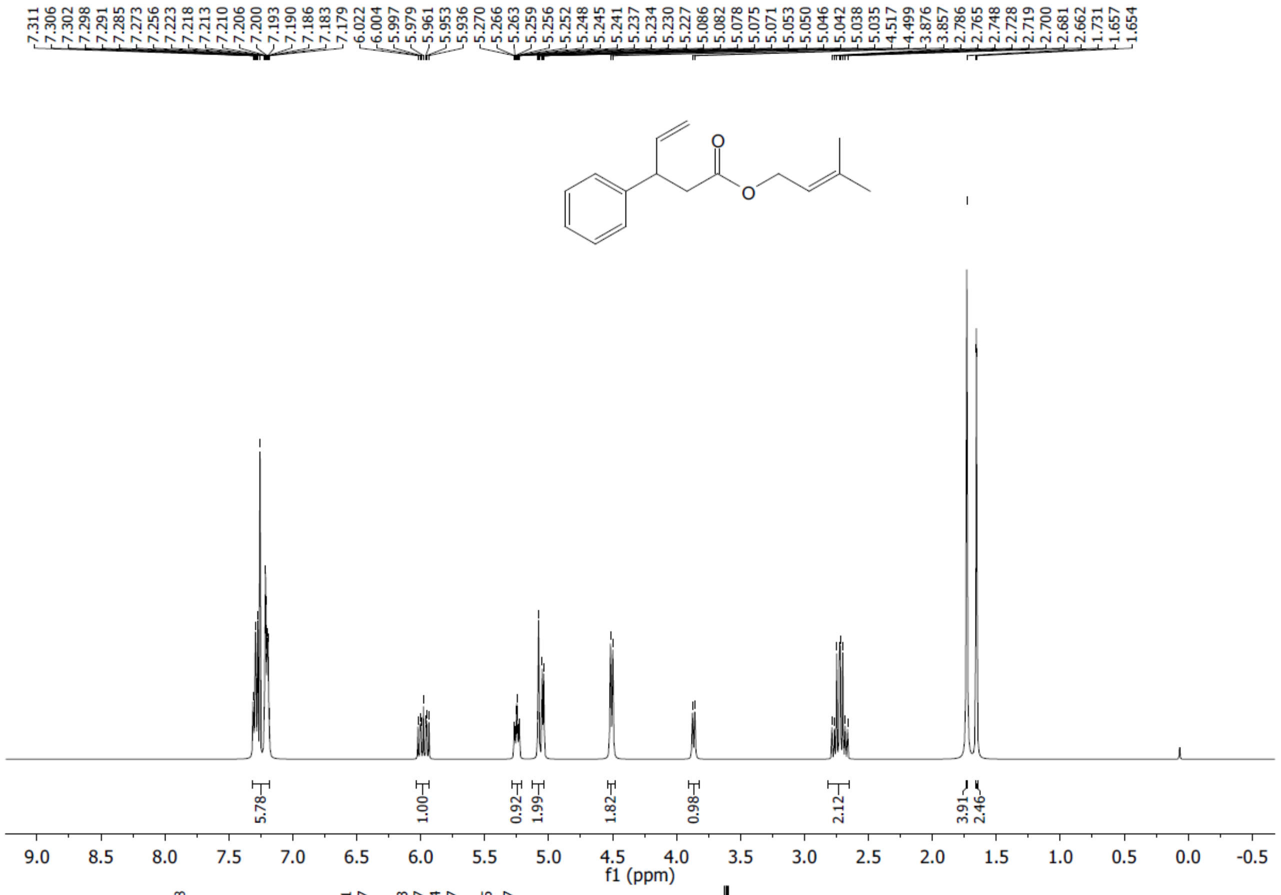

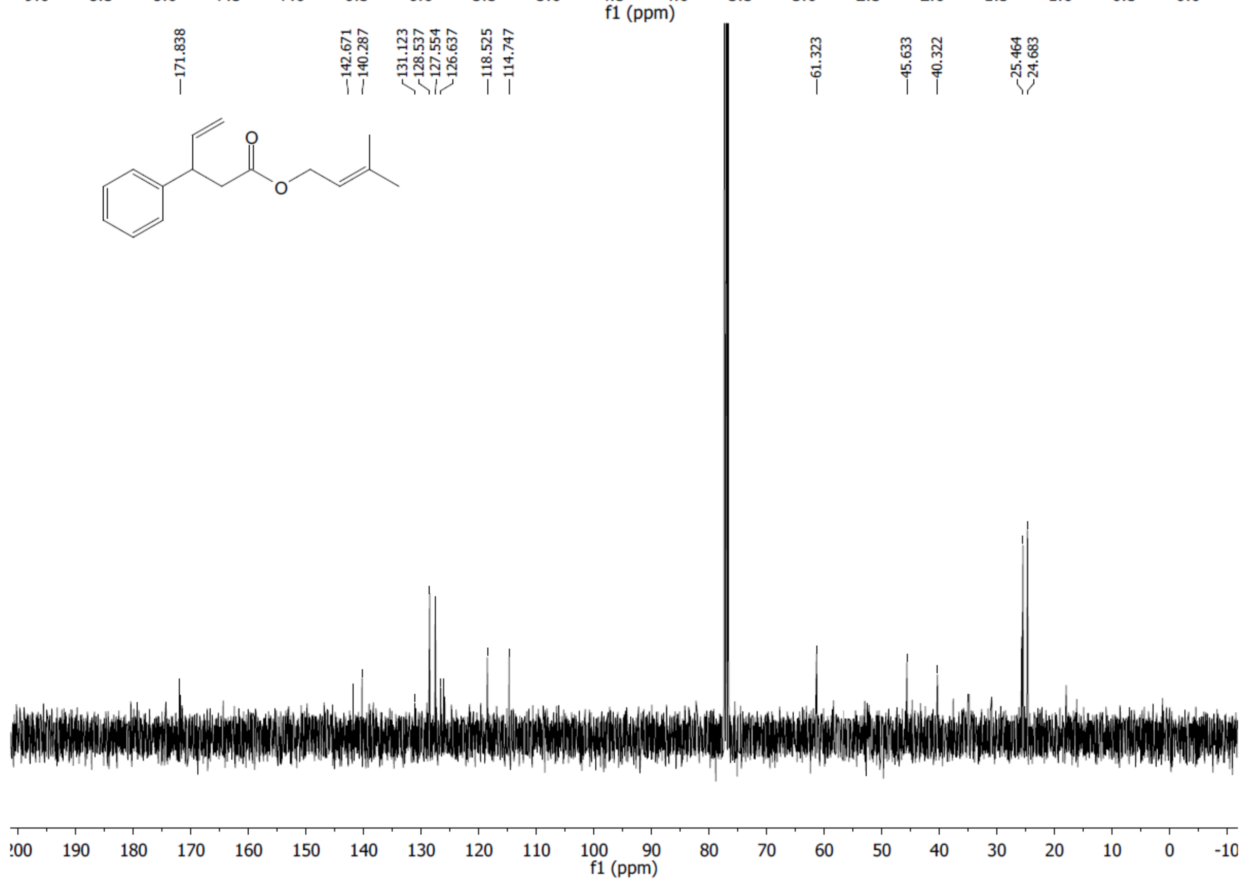

Cinnamyl 3-phenyl-4-pentenoate

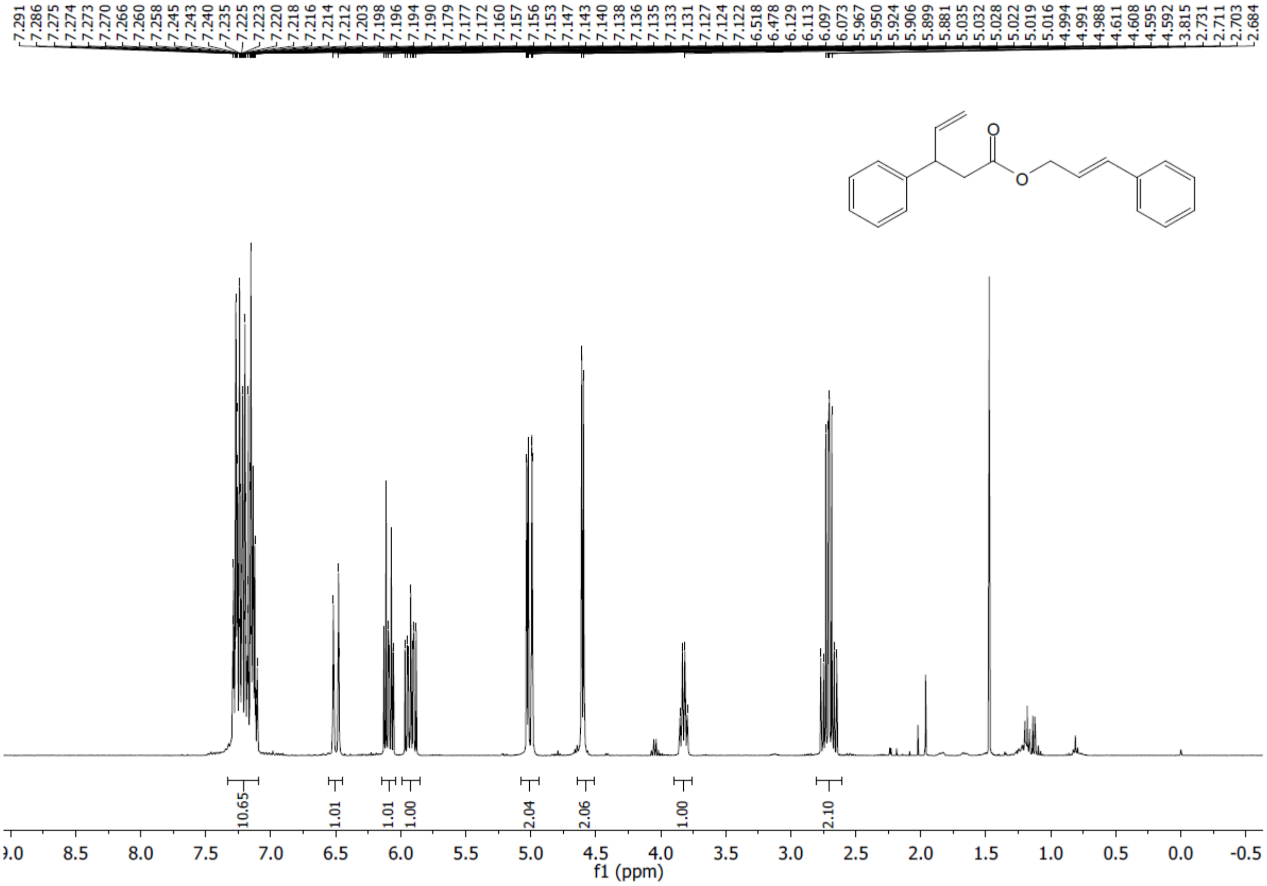

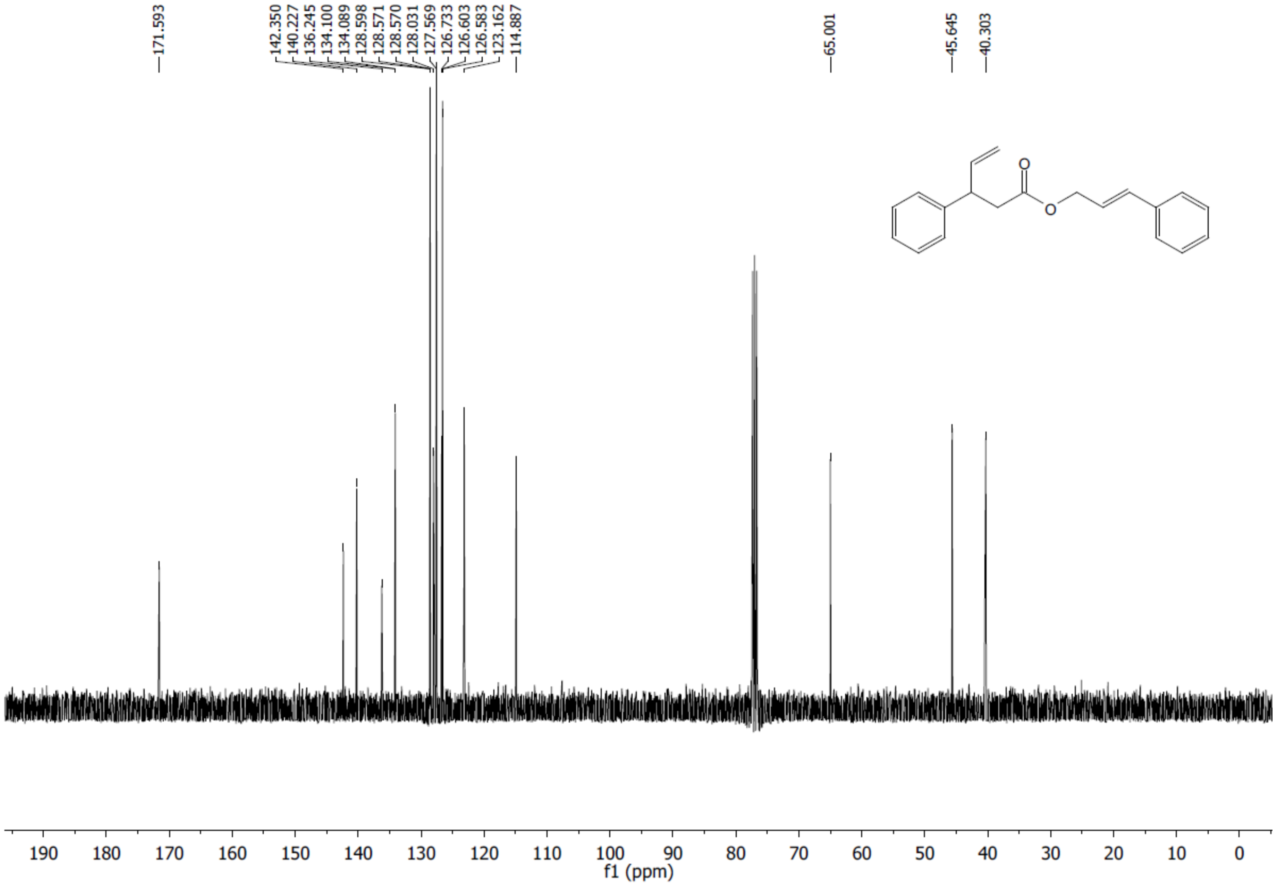

4-Phenyl-3,4-dihydro-2H-pyran-2-one (4a)

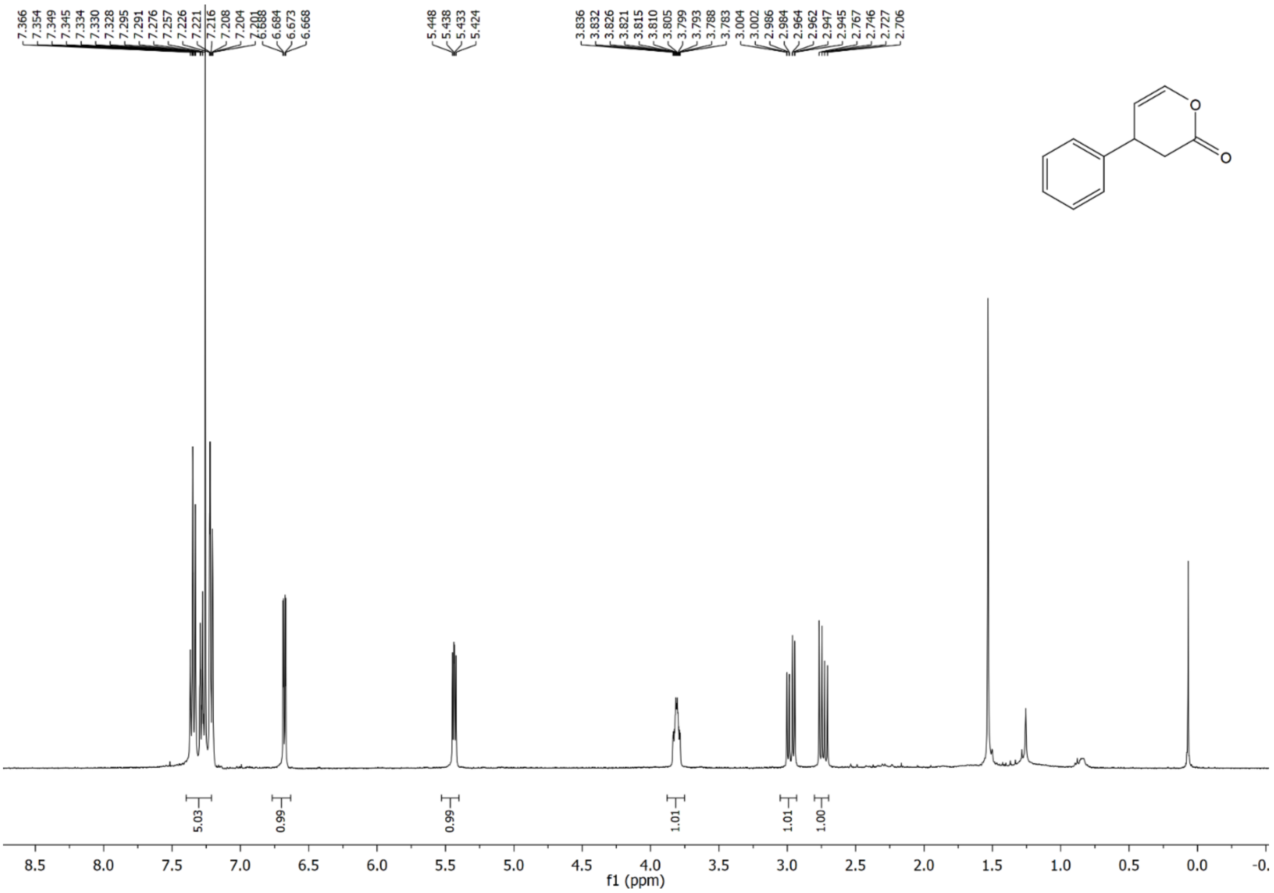

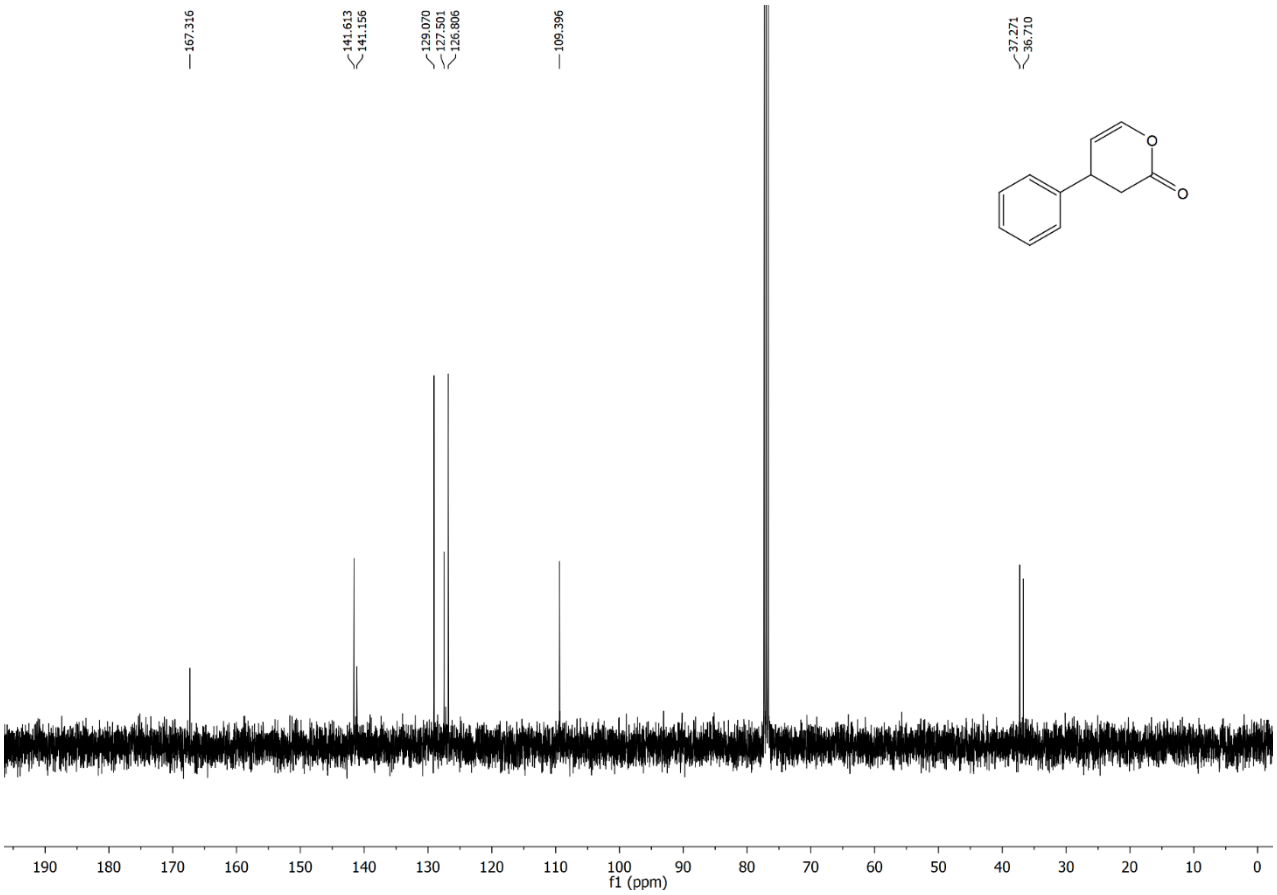

4-(4-Bromophenyl)-3,4-dihydro-2*H*-pyran-2-one (4b)

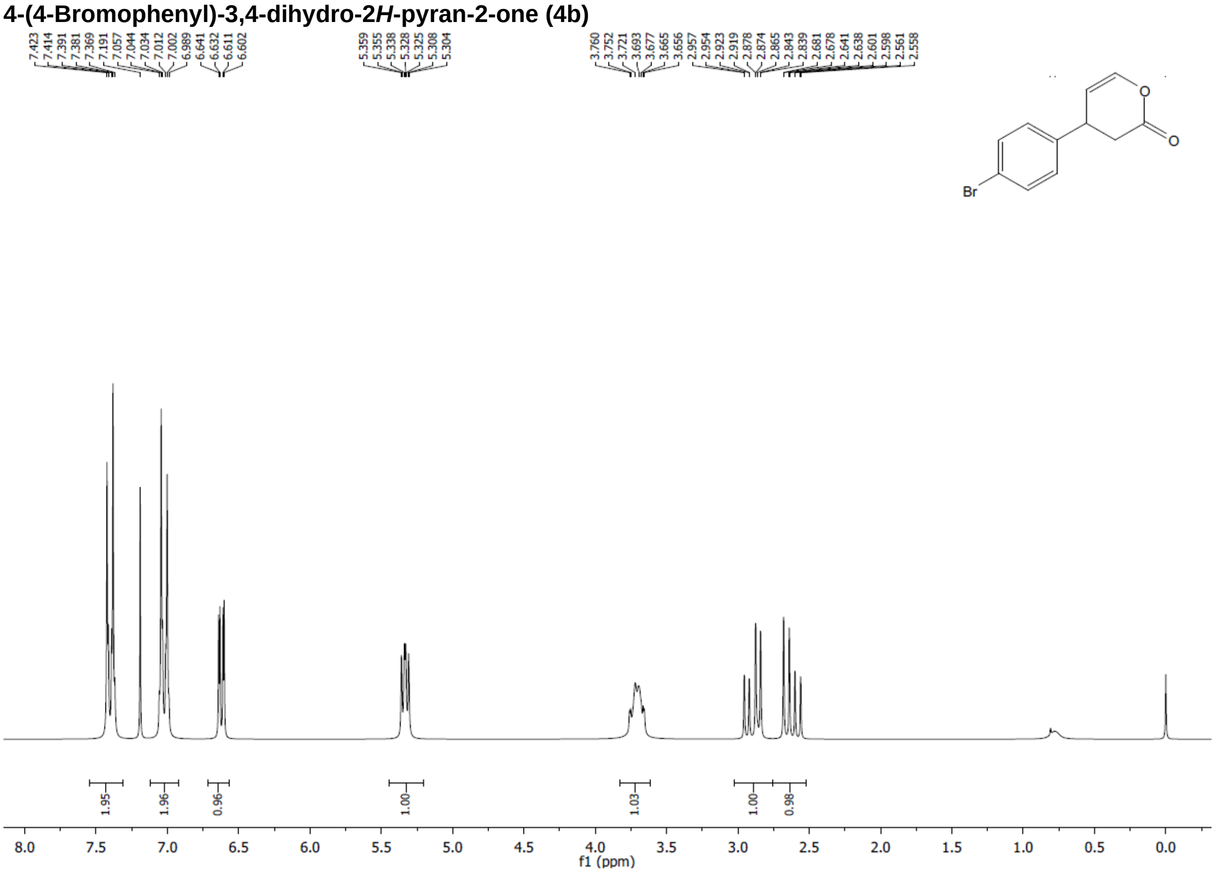

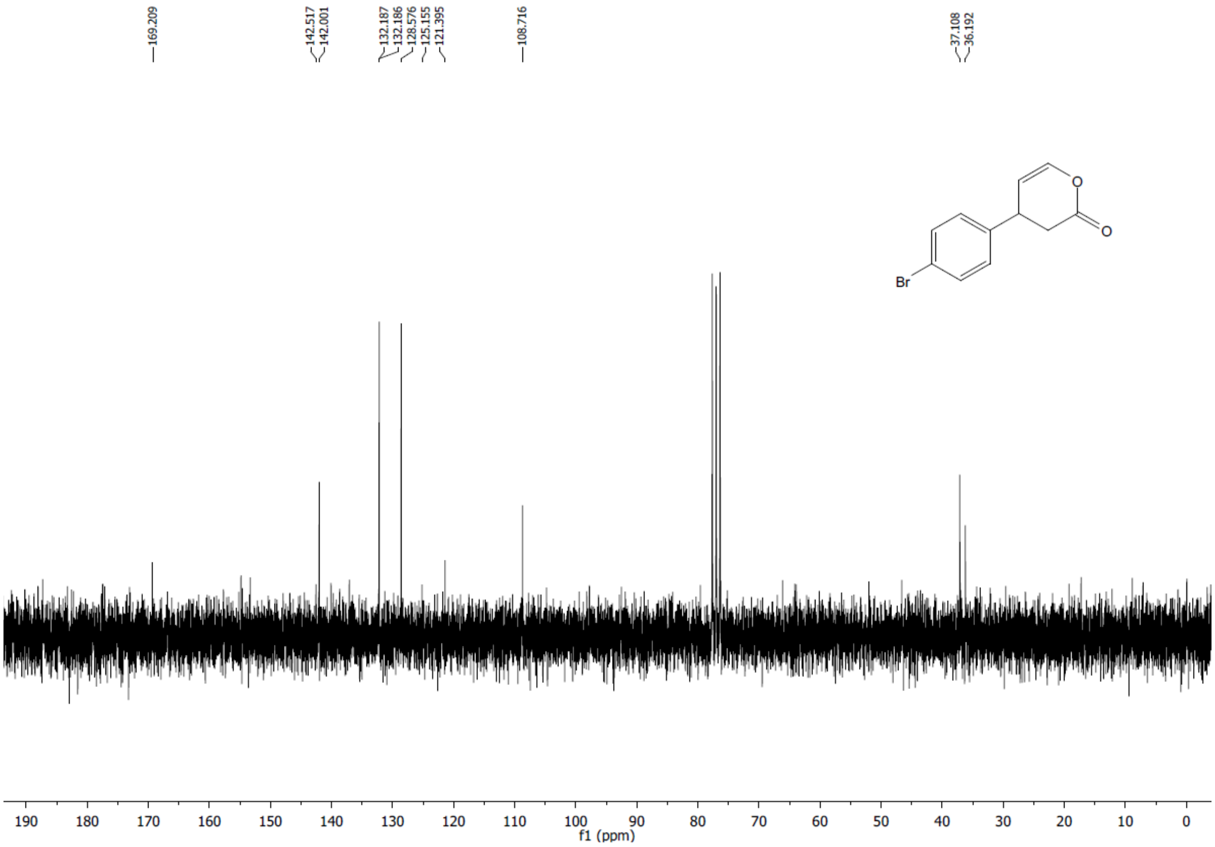

4-(4-Chlorophenyl)-3,4-dihydro-2*H*-pyran-2-one (4c)

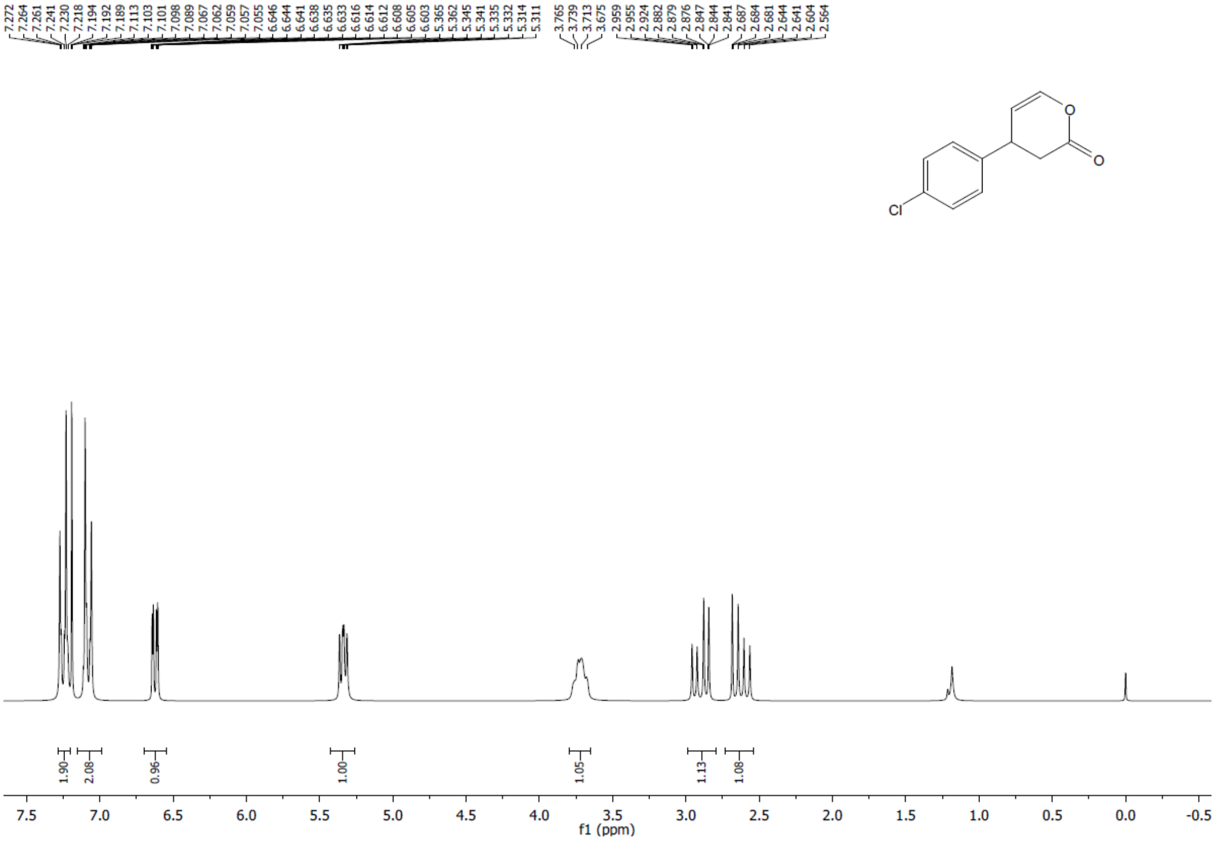

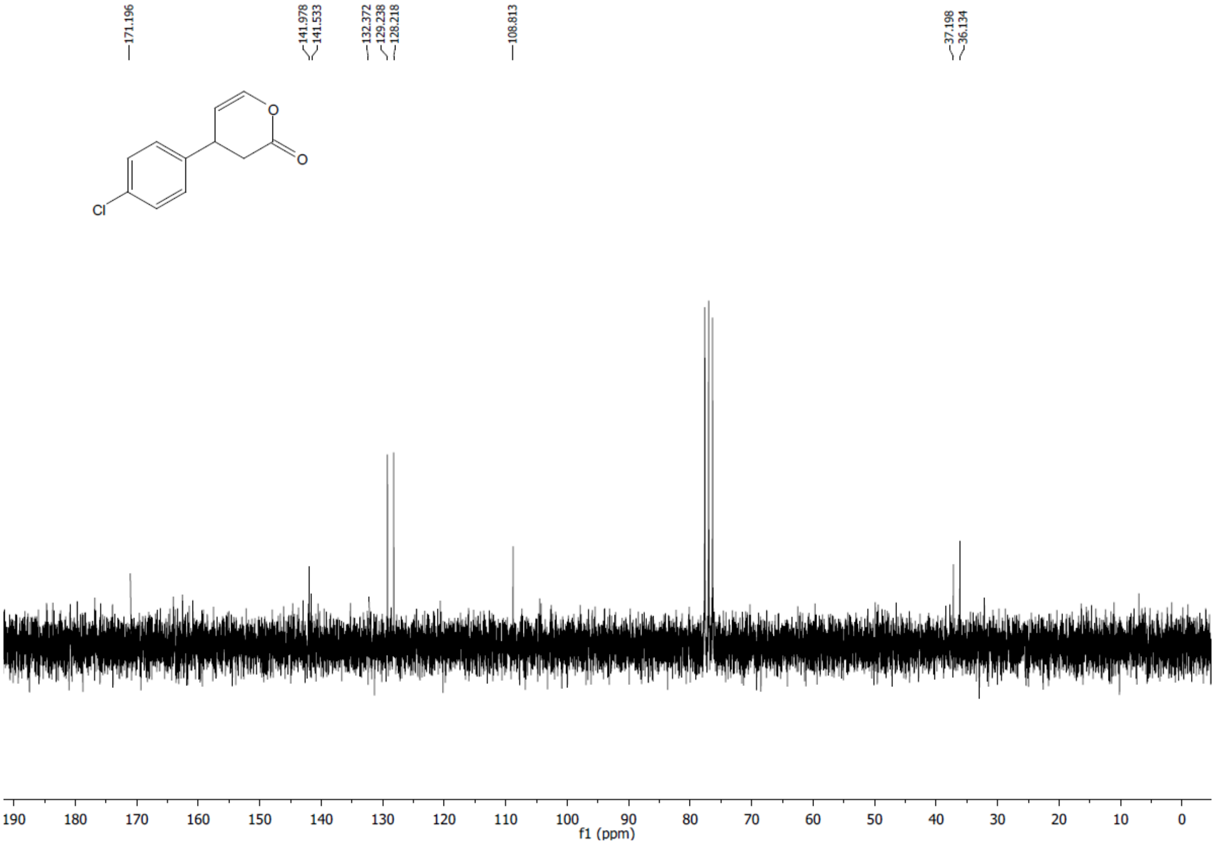

4-Propyl-3,4-dihydro-2*H*-pyran-2-one (4d)

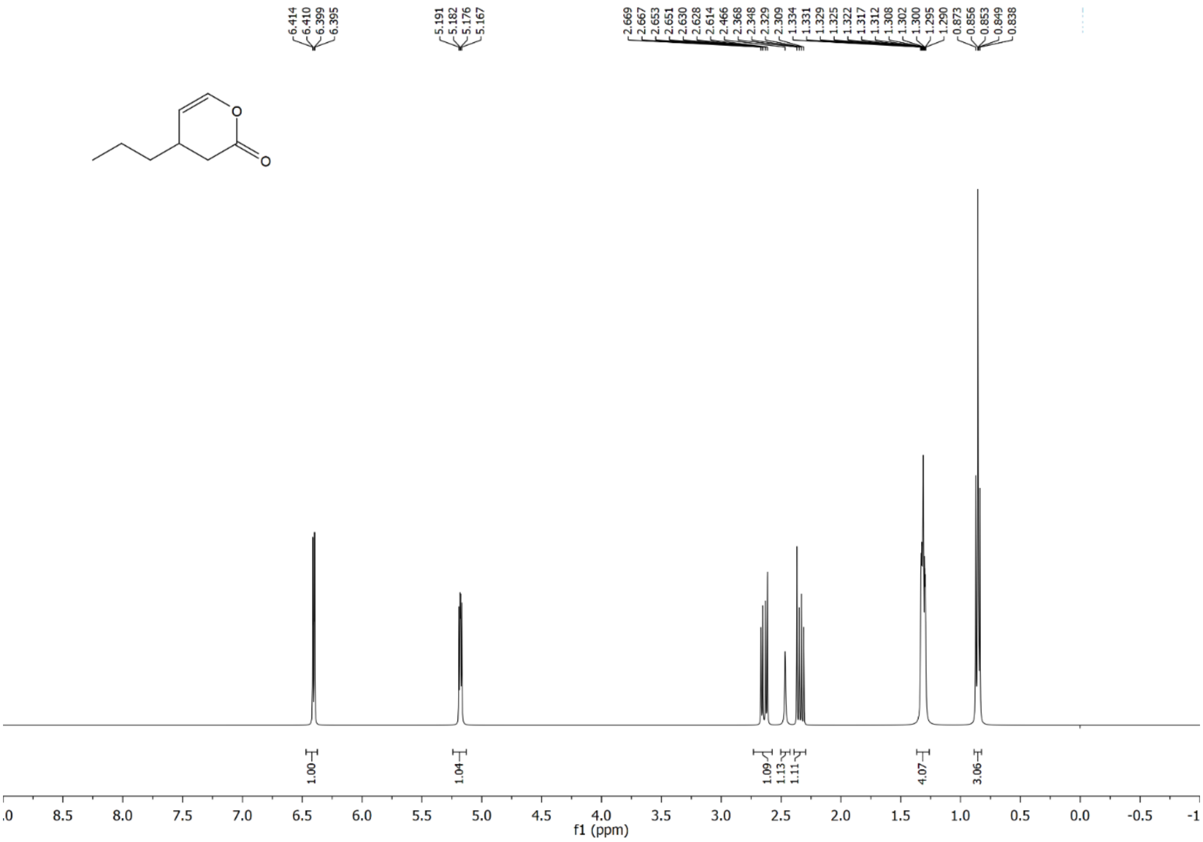

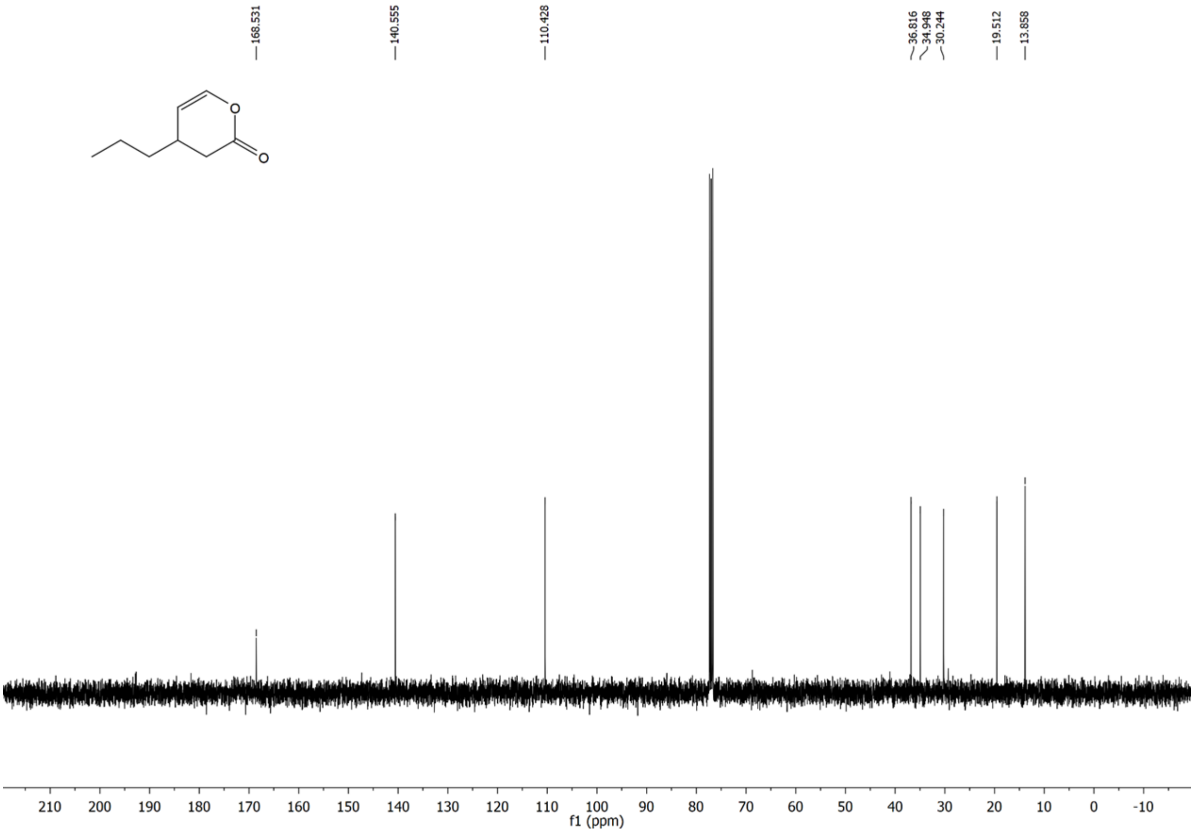

4-Phenyl-3,4-dihydrooxepin-2*(7H)*-one (5a)

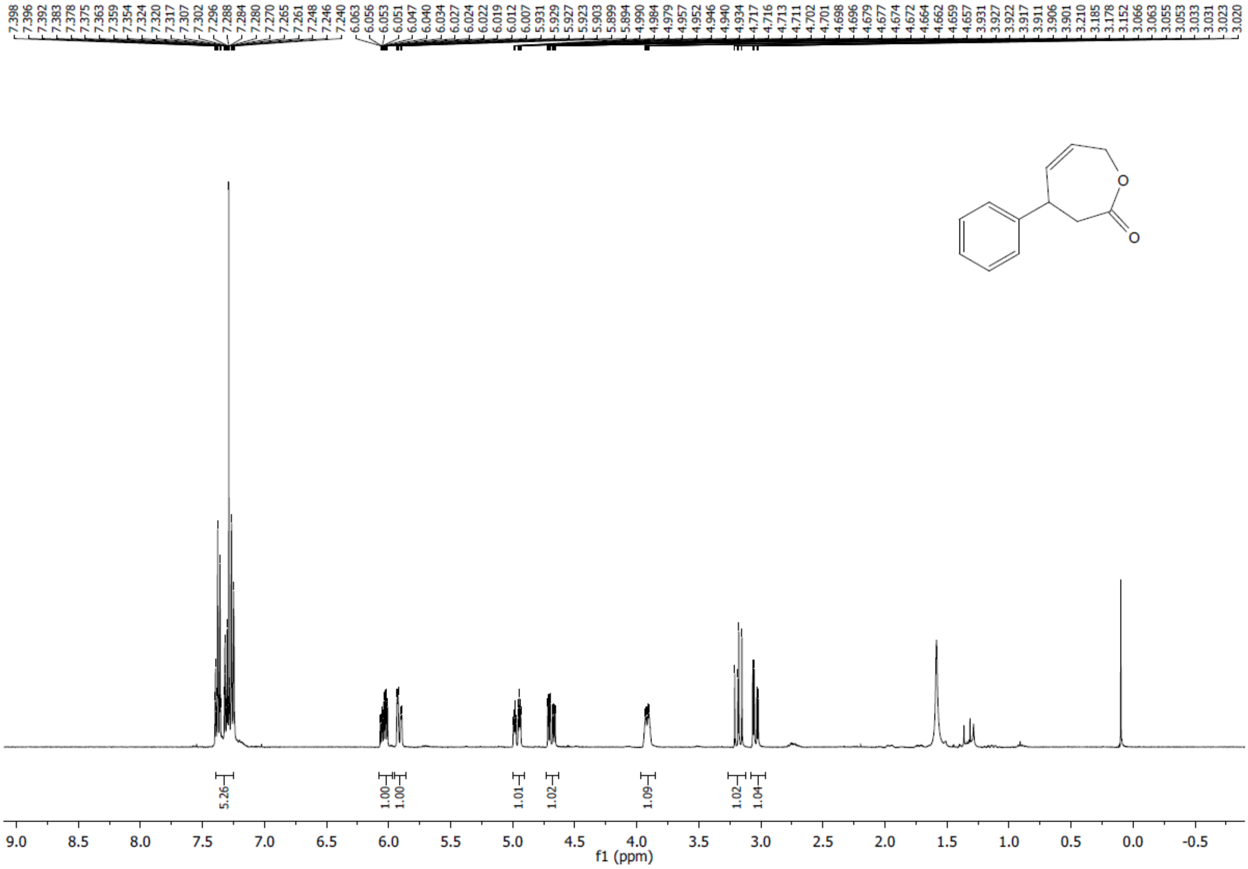

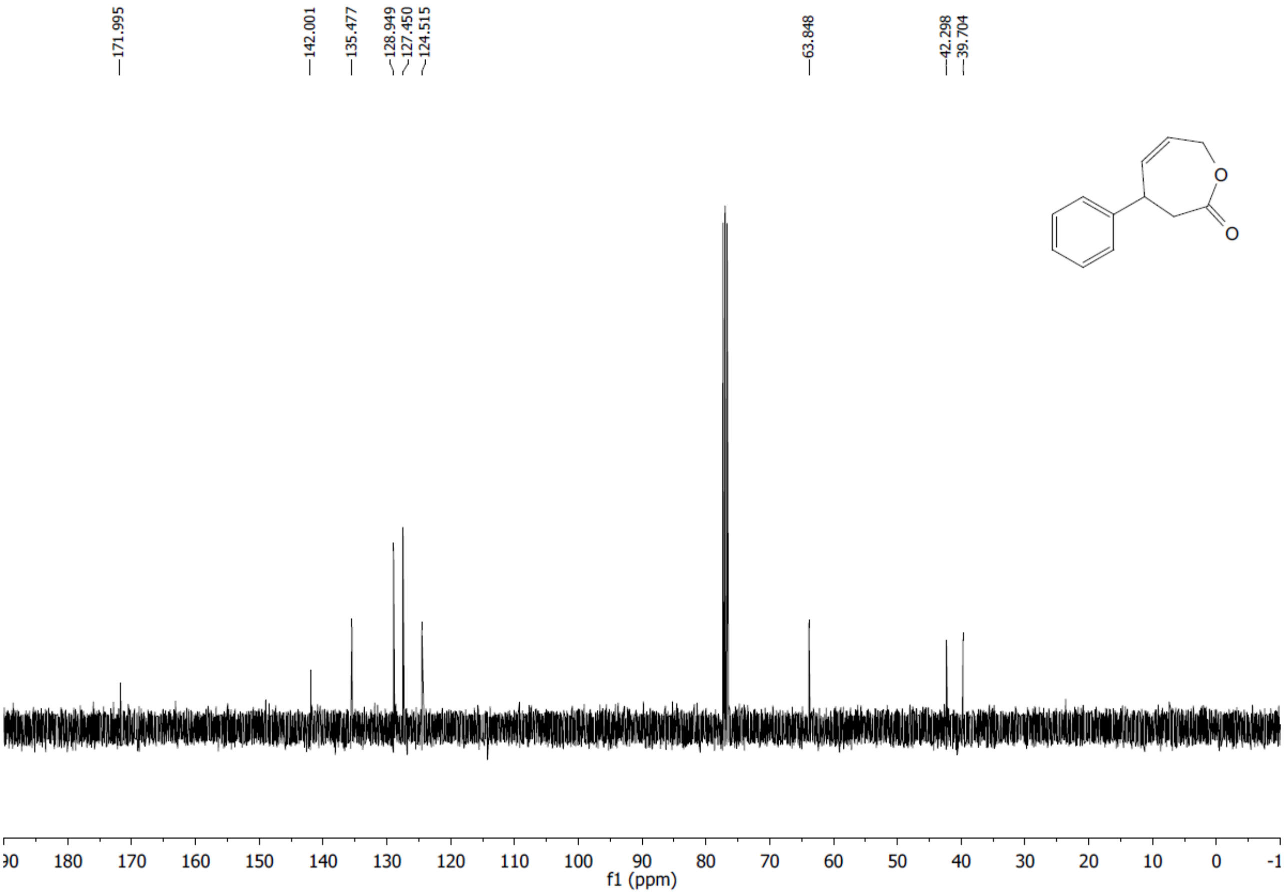

4-(4-Bromophenyl)-3,4-dihydrooxepin-2*(7H)*-one (5b)

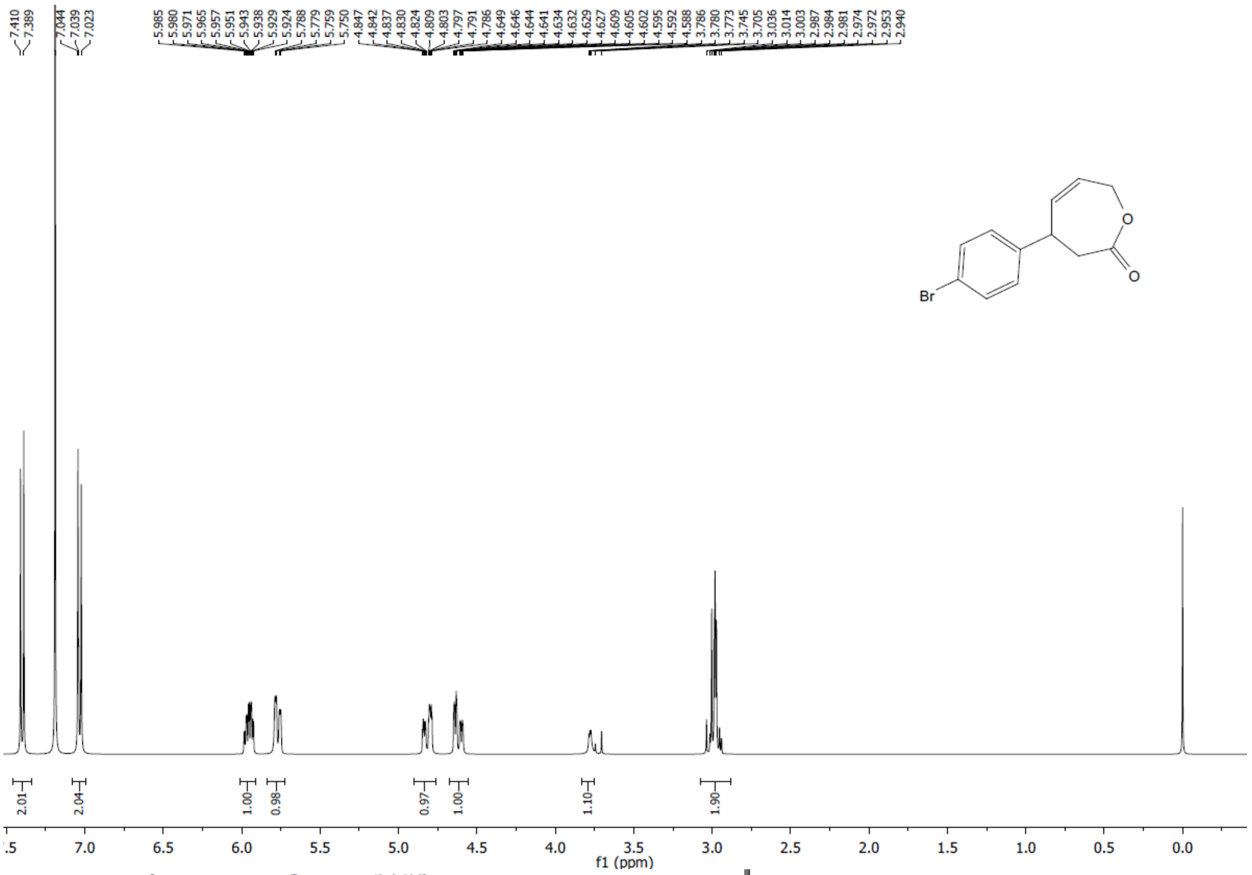

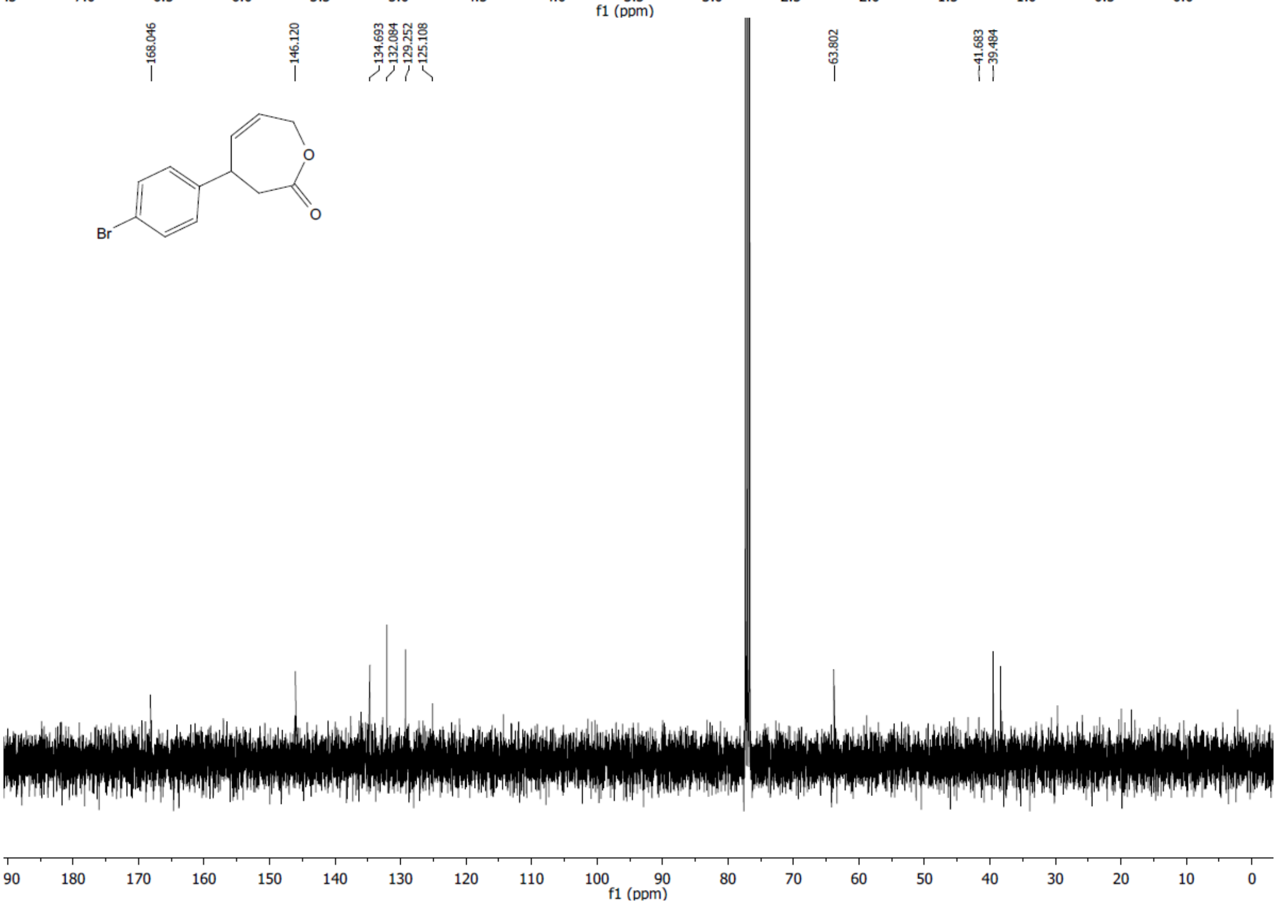

4-(4-Chlorophenyl)-3,4-dihydrooxepin-2*(7H)*-one (5c)

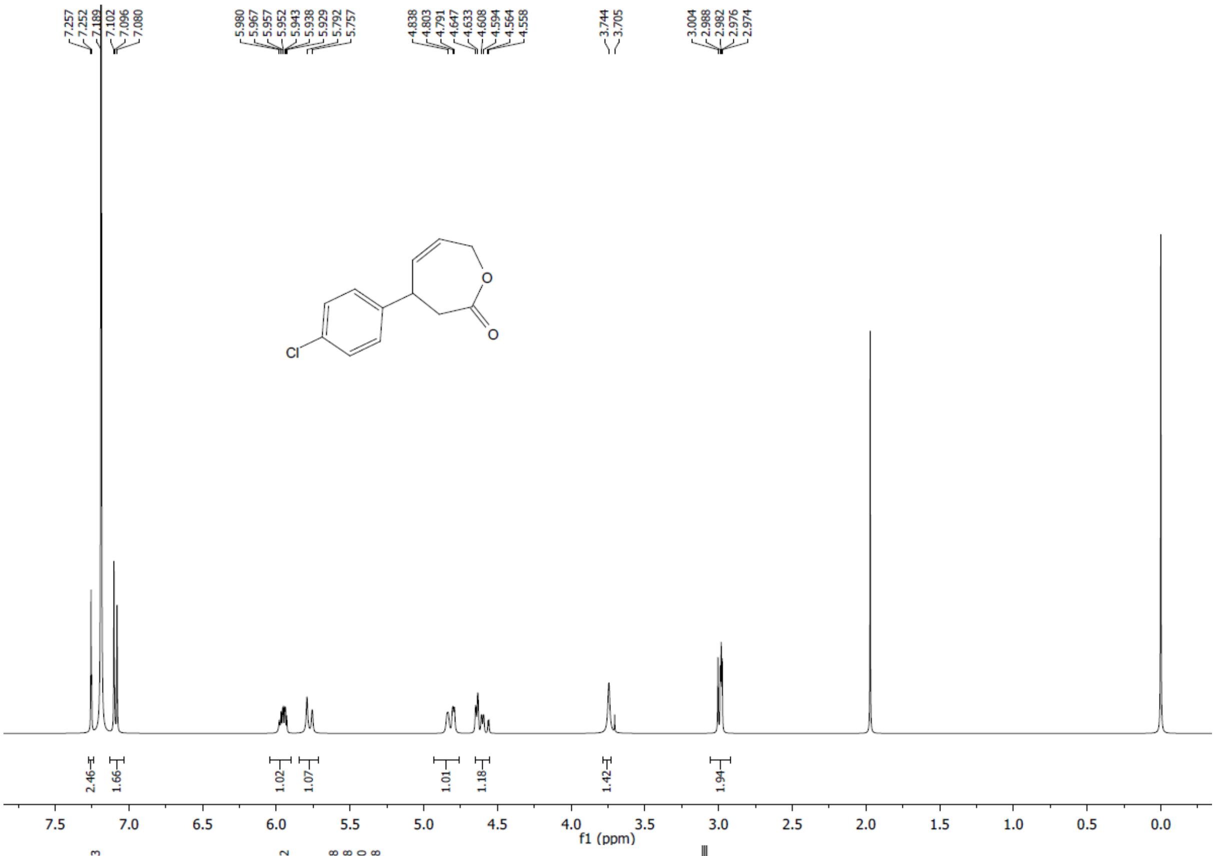

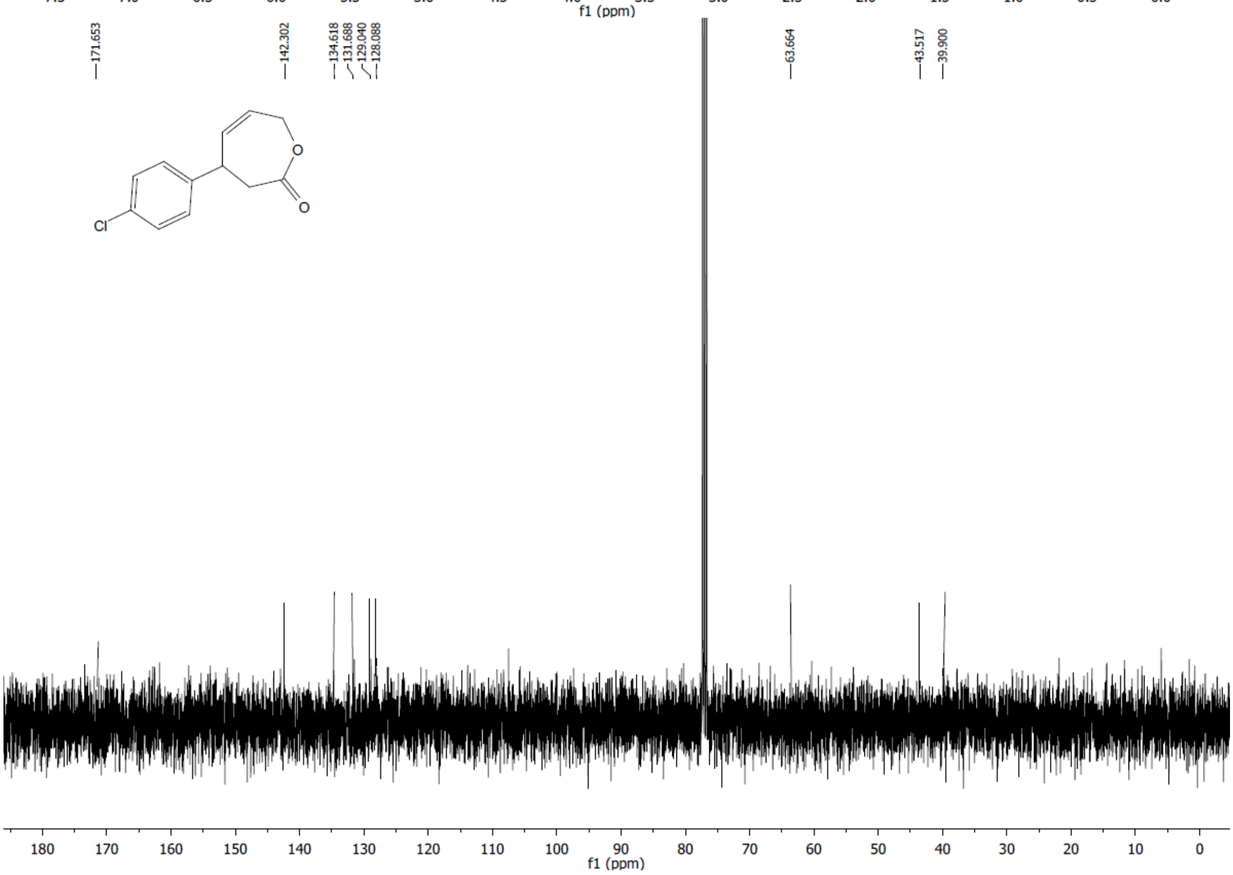

4-Propyl-3,4-dihydrooxepin-2*(7H)*-one (5d)

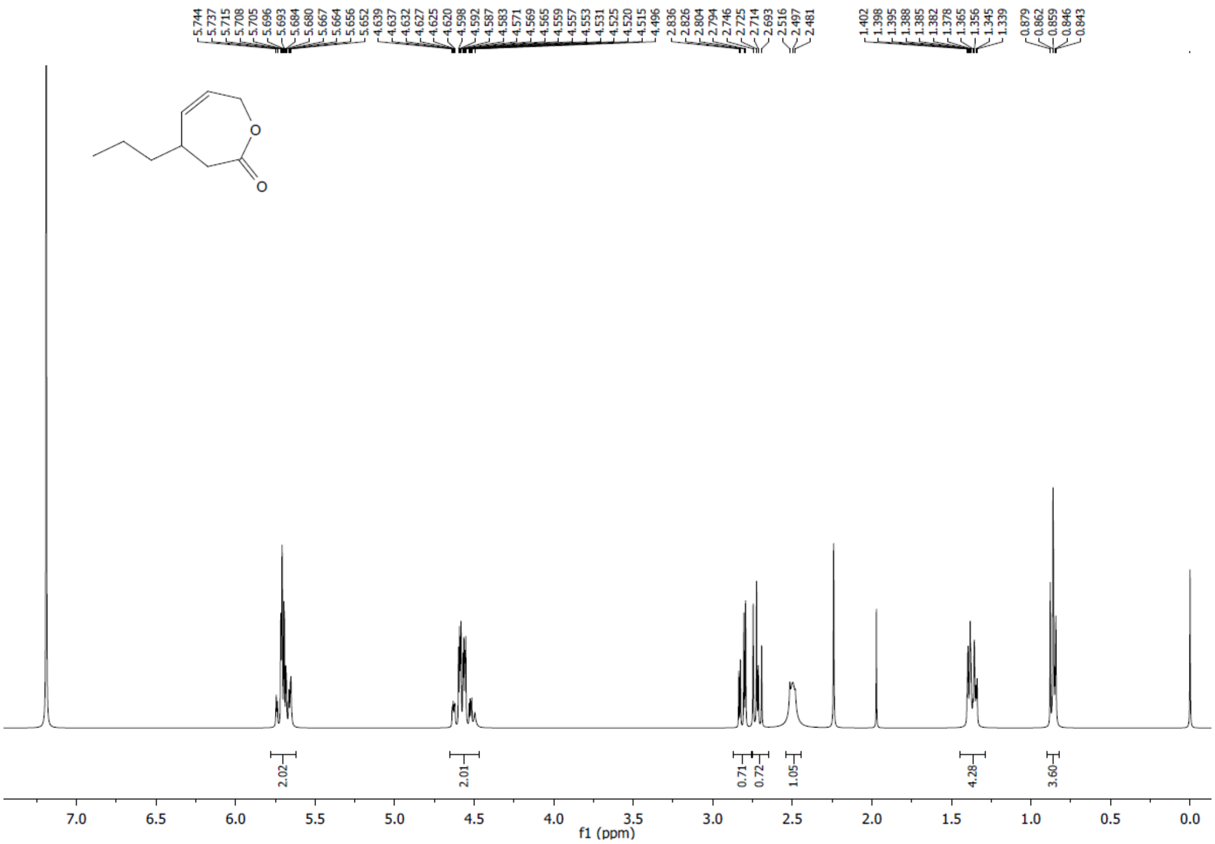

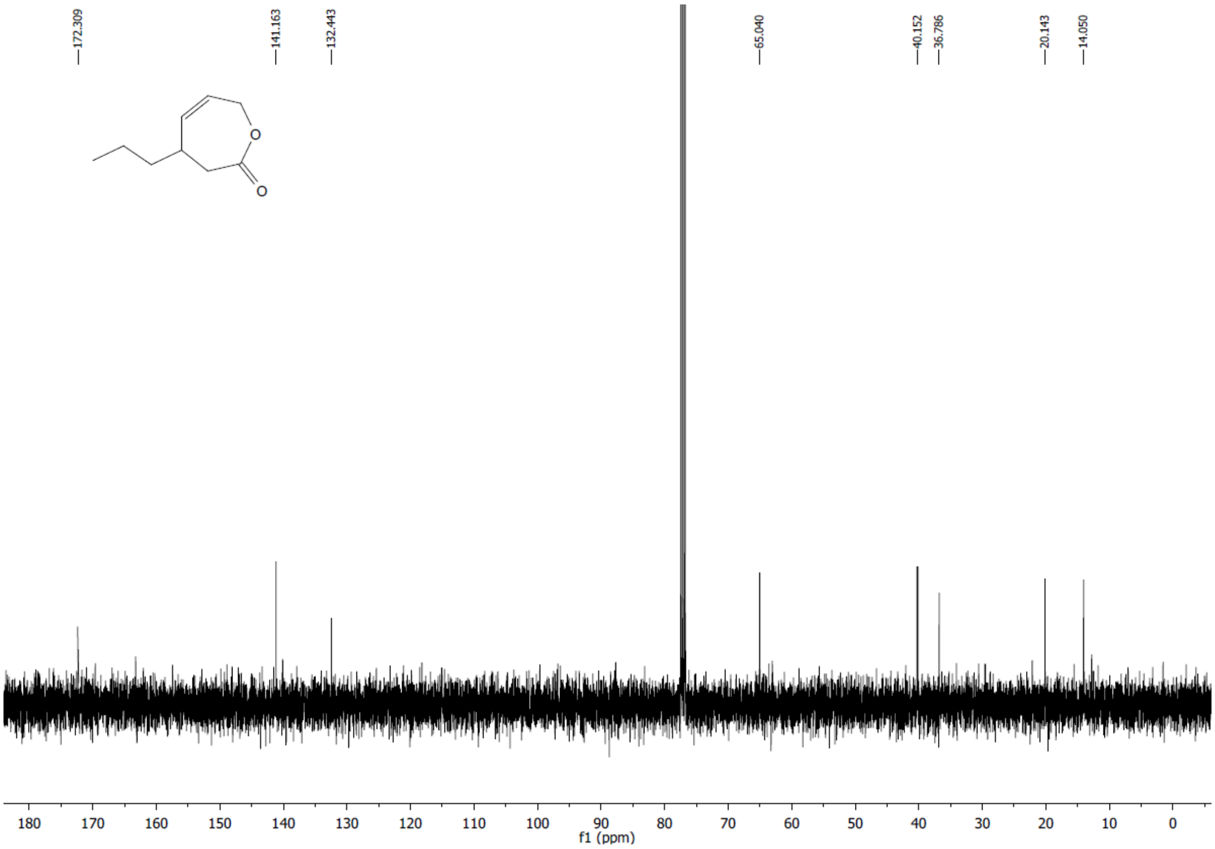

N-allyl-N-benzyl-3-phenyl-4-pentenamide (7a)

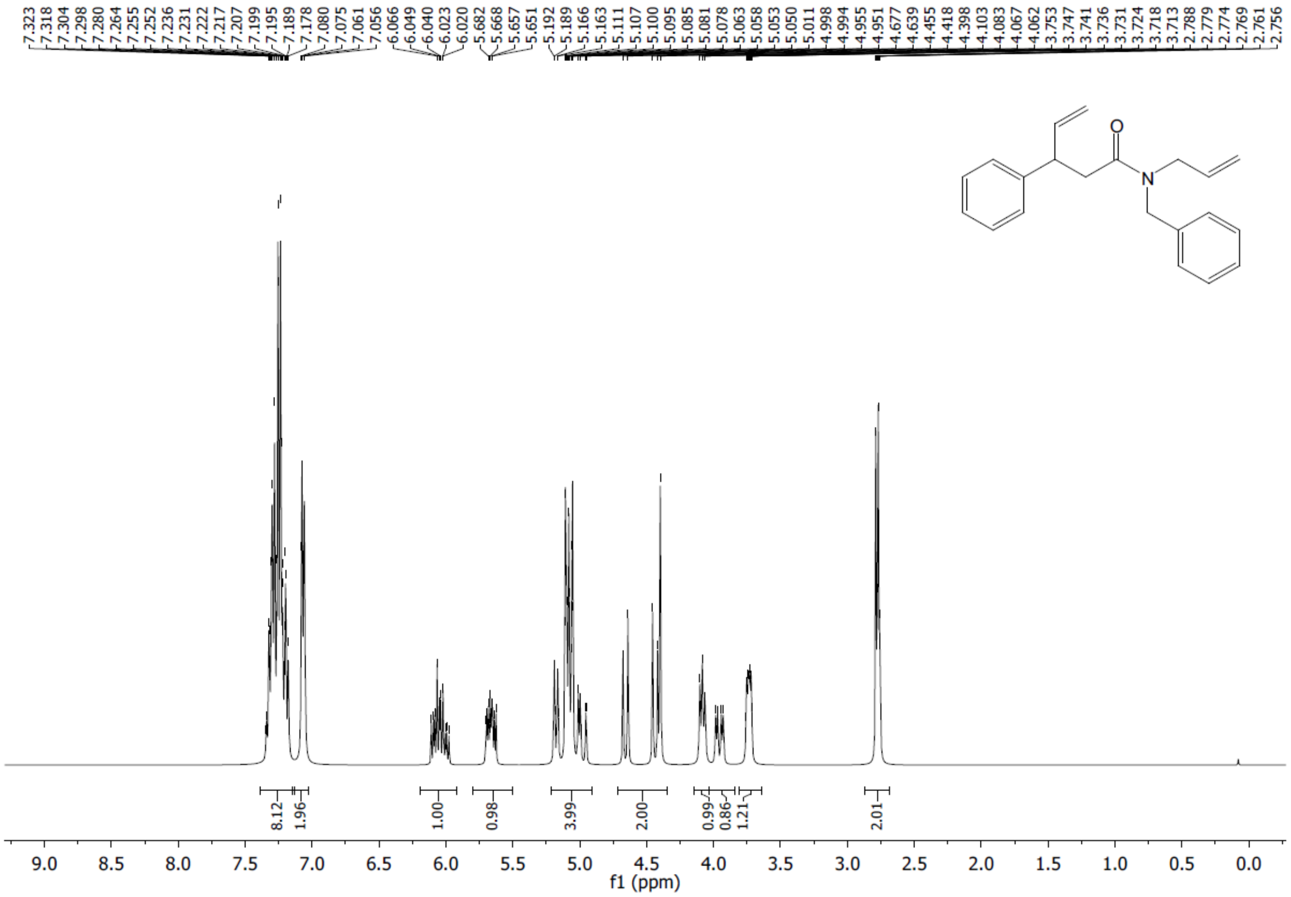

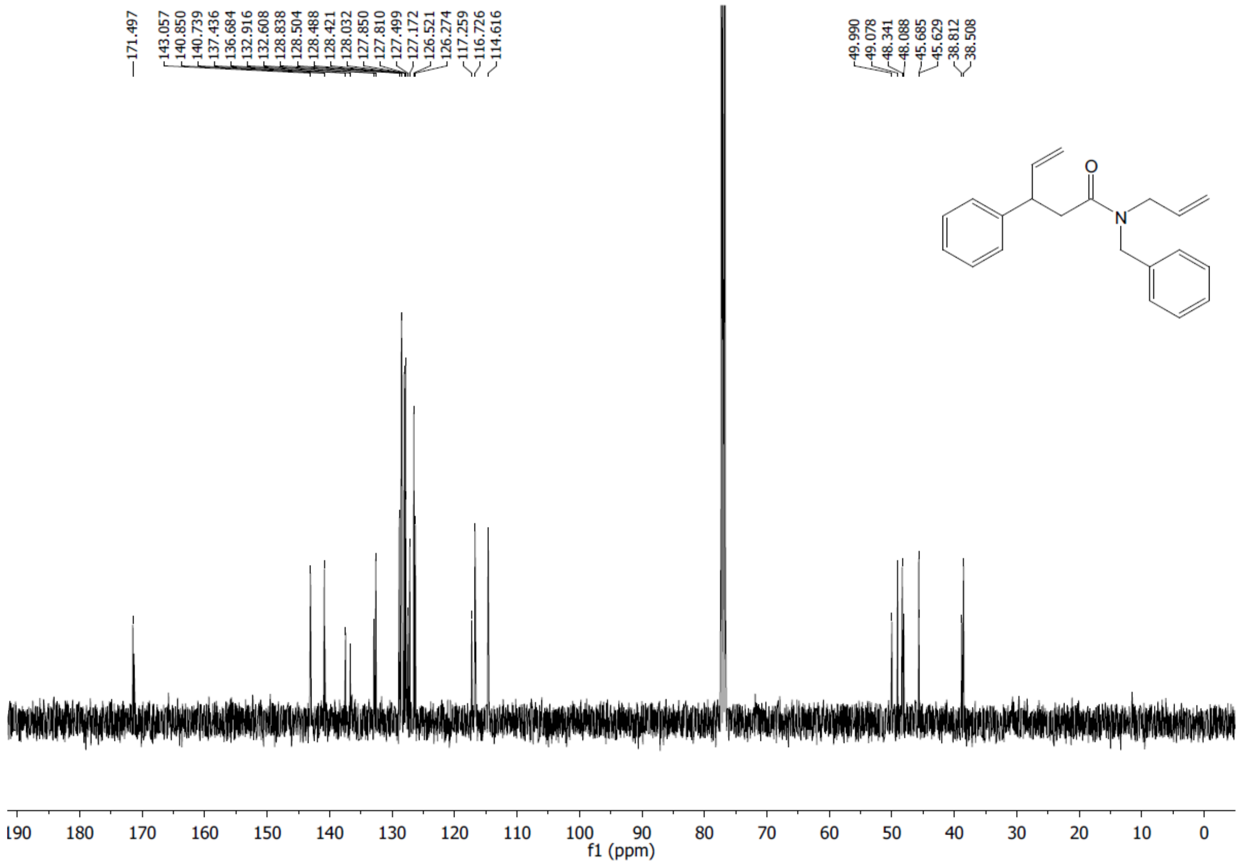

N-allyl-N-benzyl-3-(4-bromophenyl)-4-pentenamide (7b)

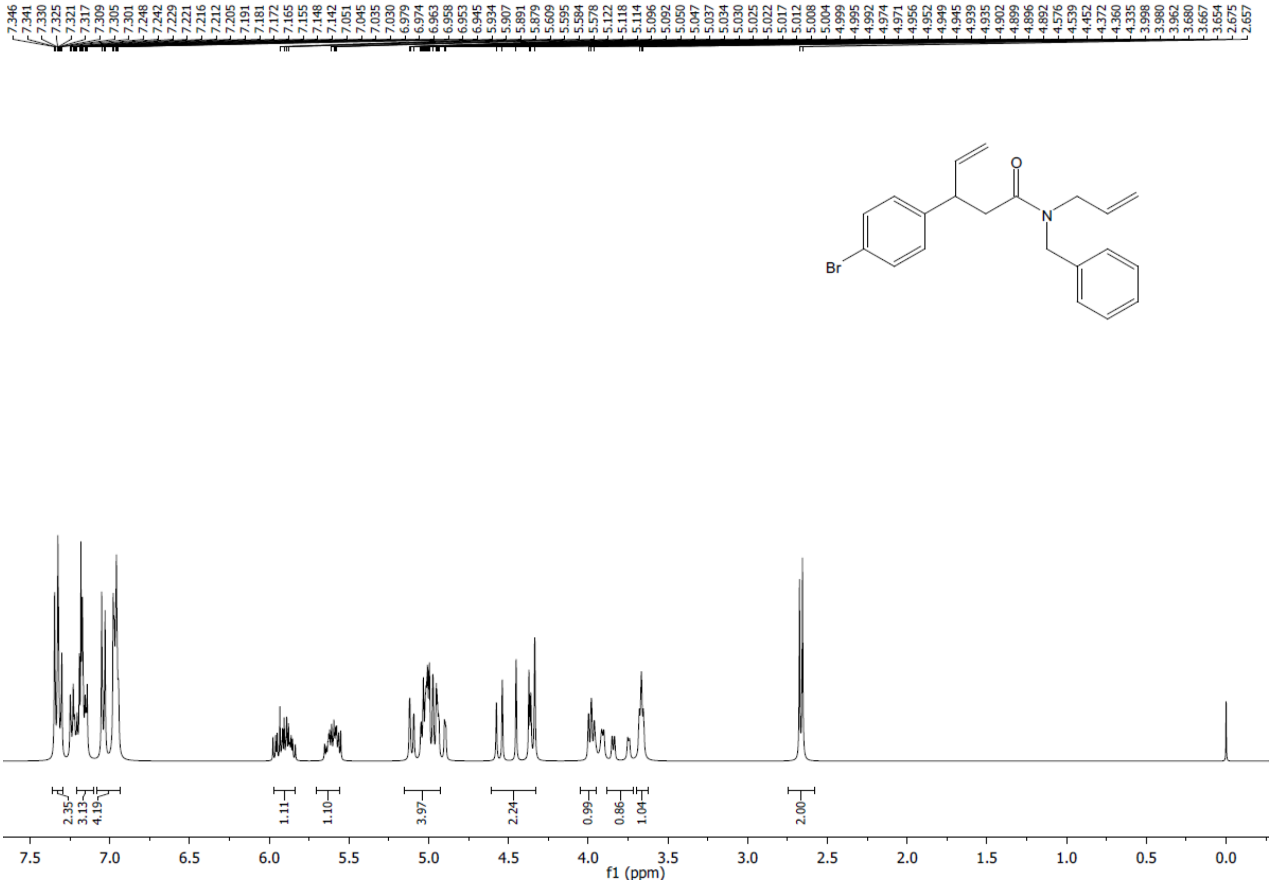

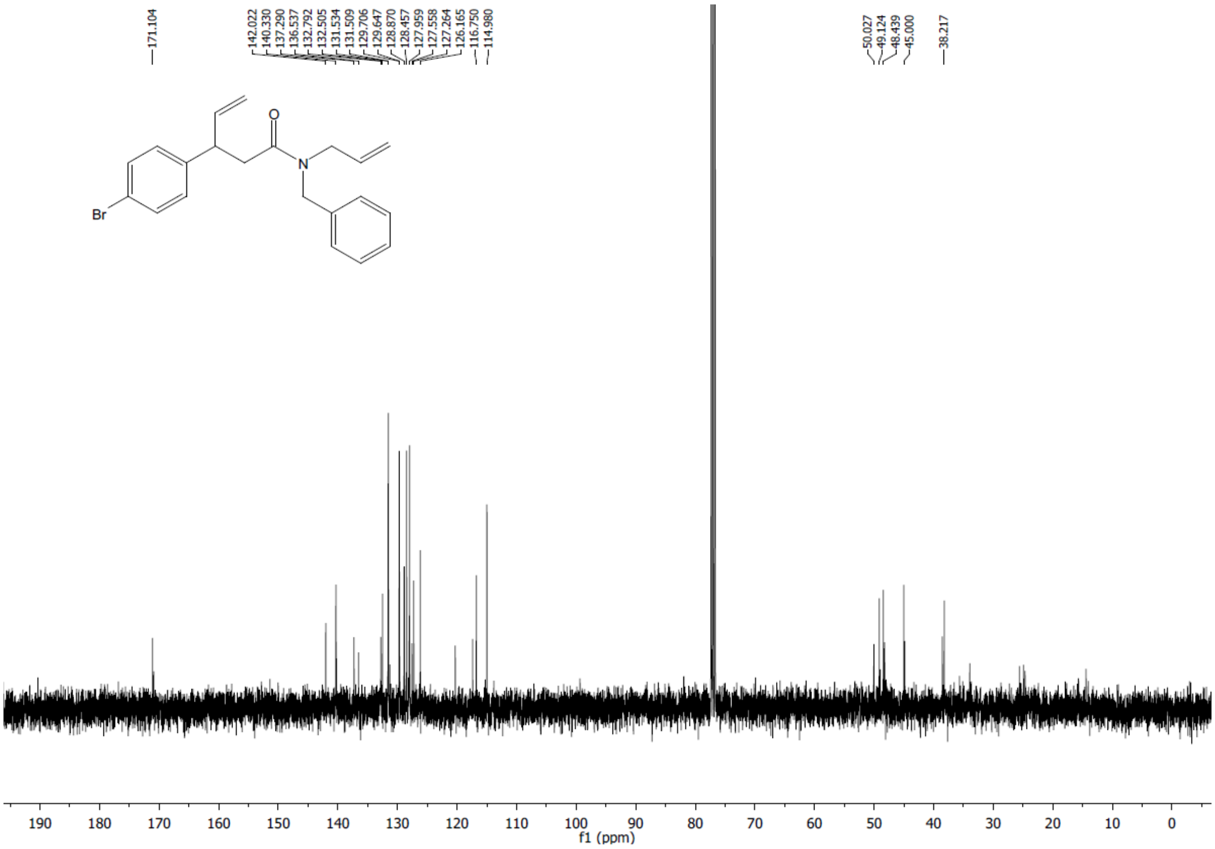

N-allyl-N-benzyl-3-(4-chlorophenyl)-4-pentenamide (7c)

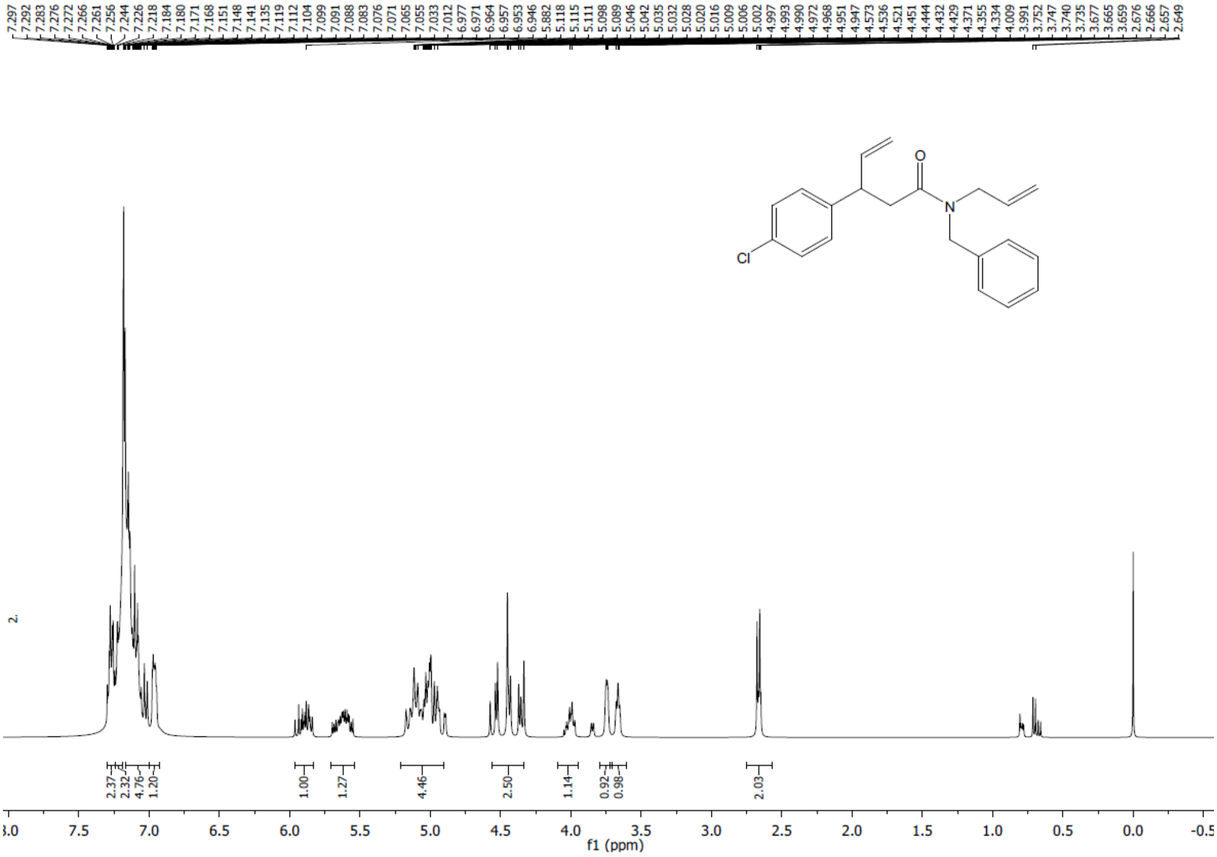

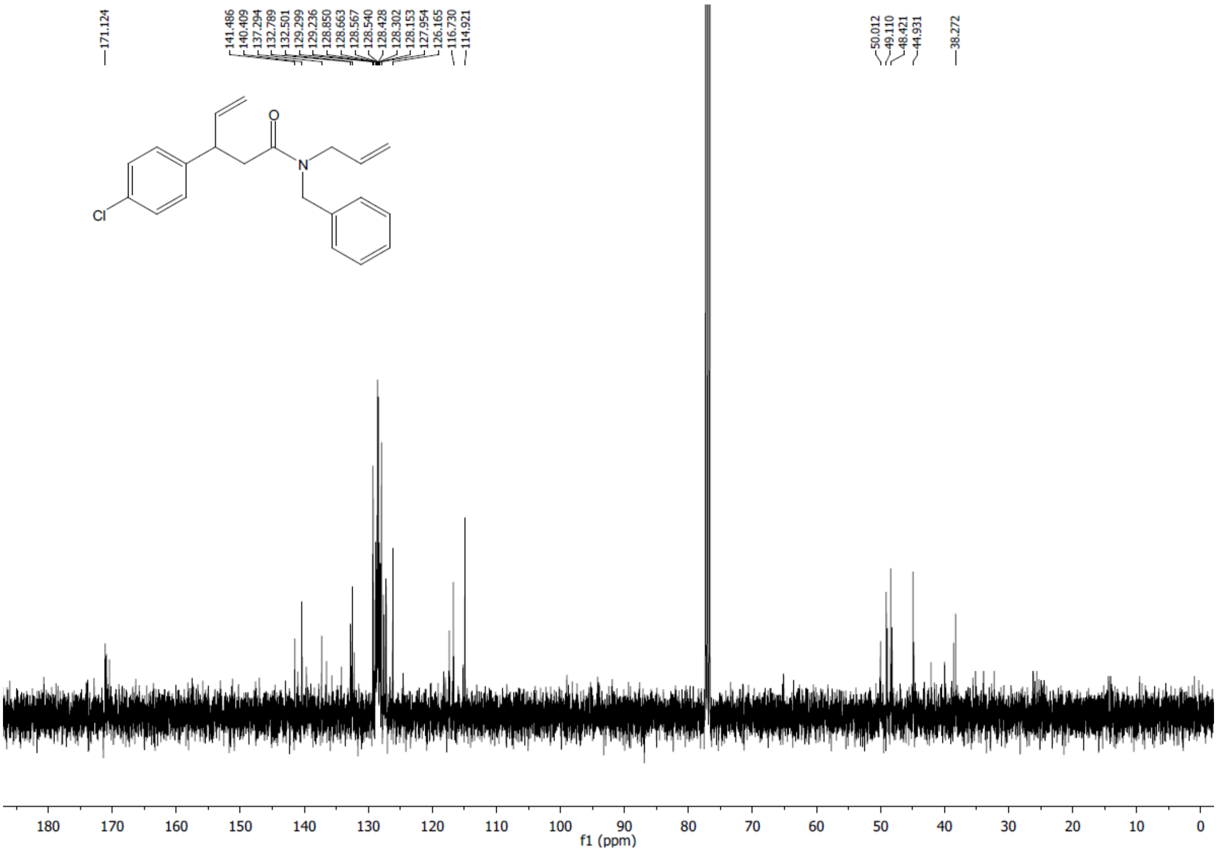

N-allyl-N-benzyl-3-propyl-4-pentenamide (7d)

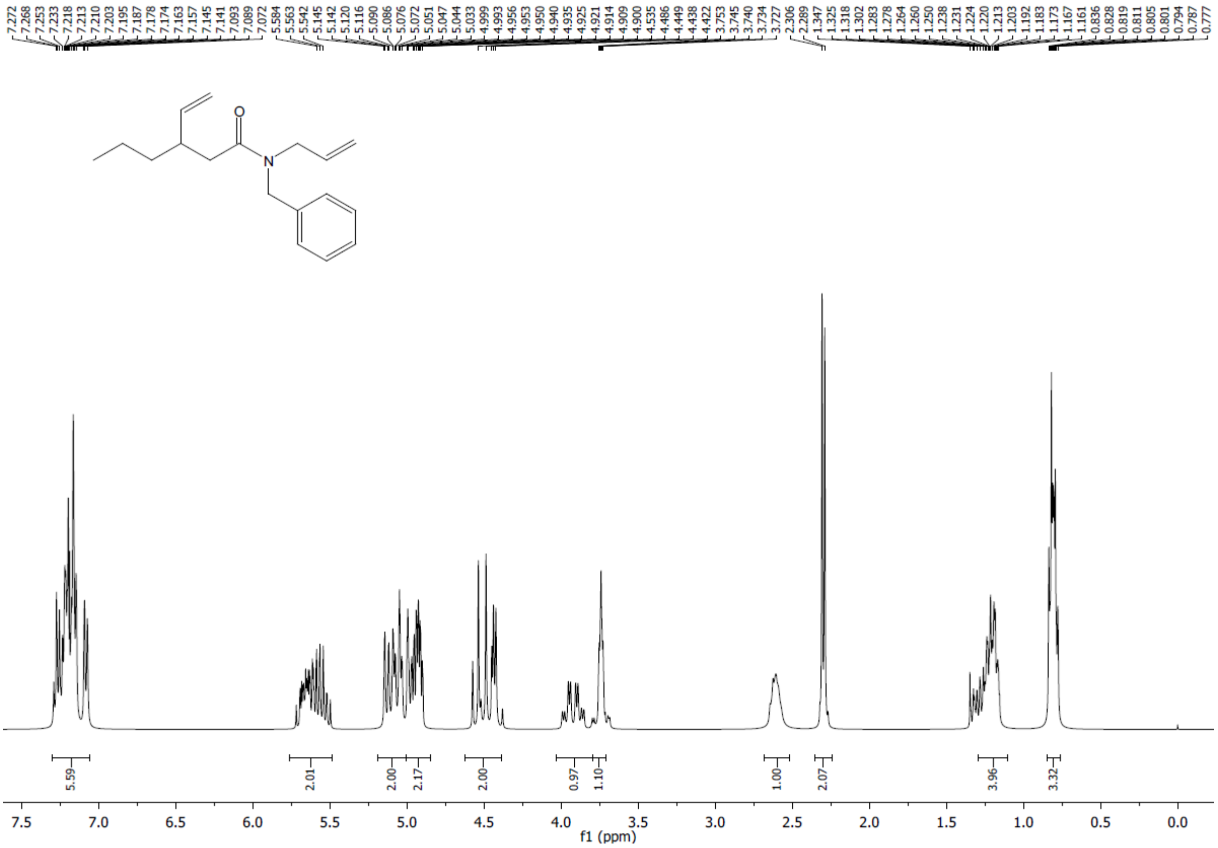

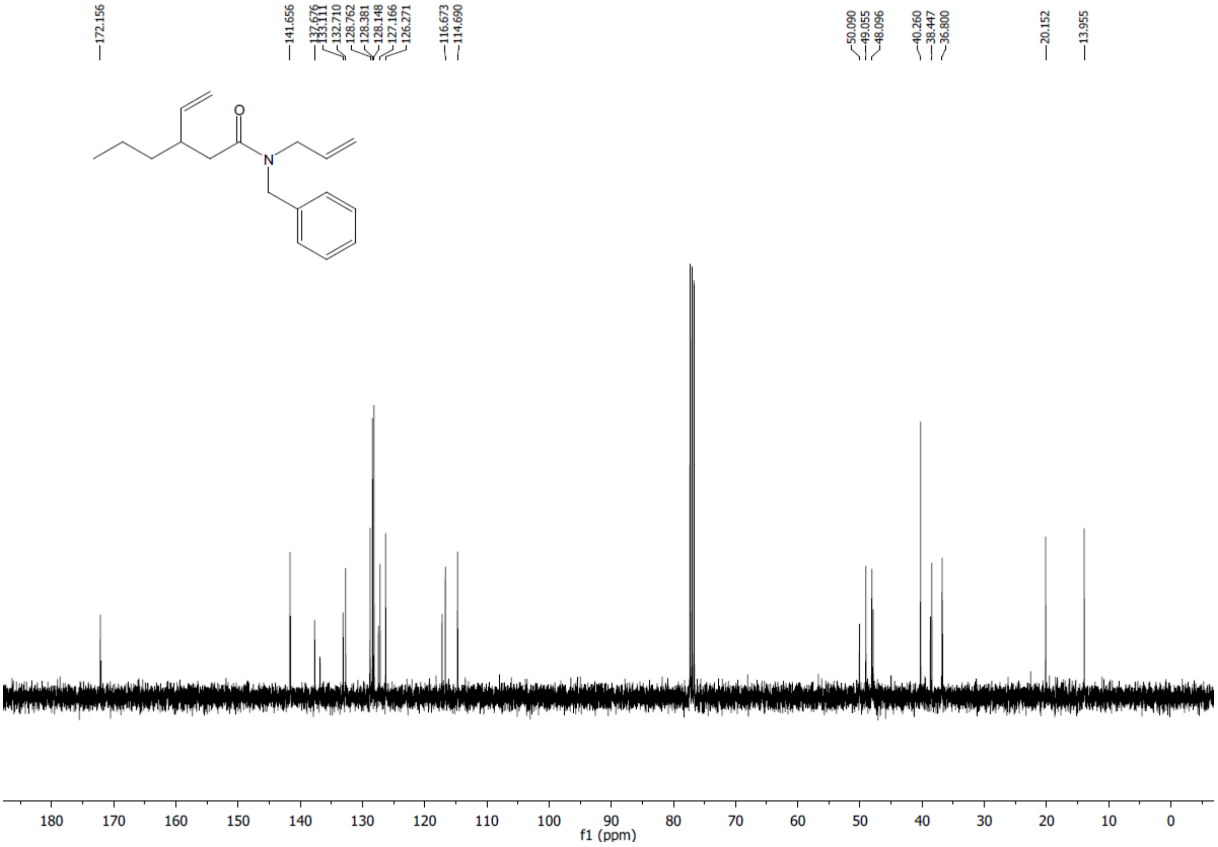

1-Benzyl-4-phenyl-1,3,4,7-tetrahydro-azepin-2-one (8a)

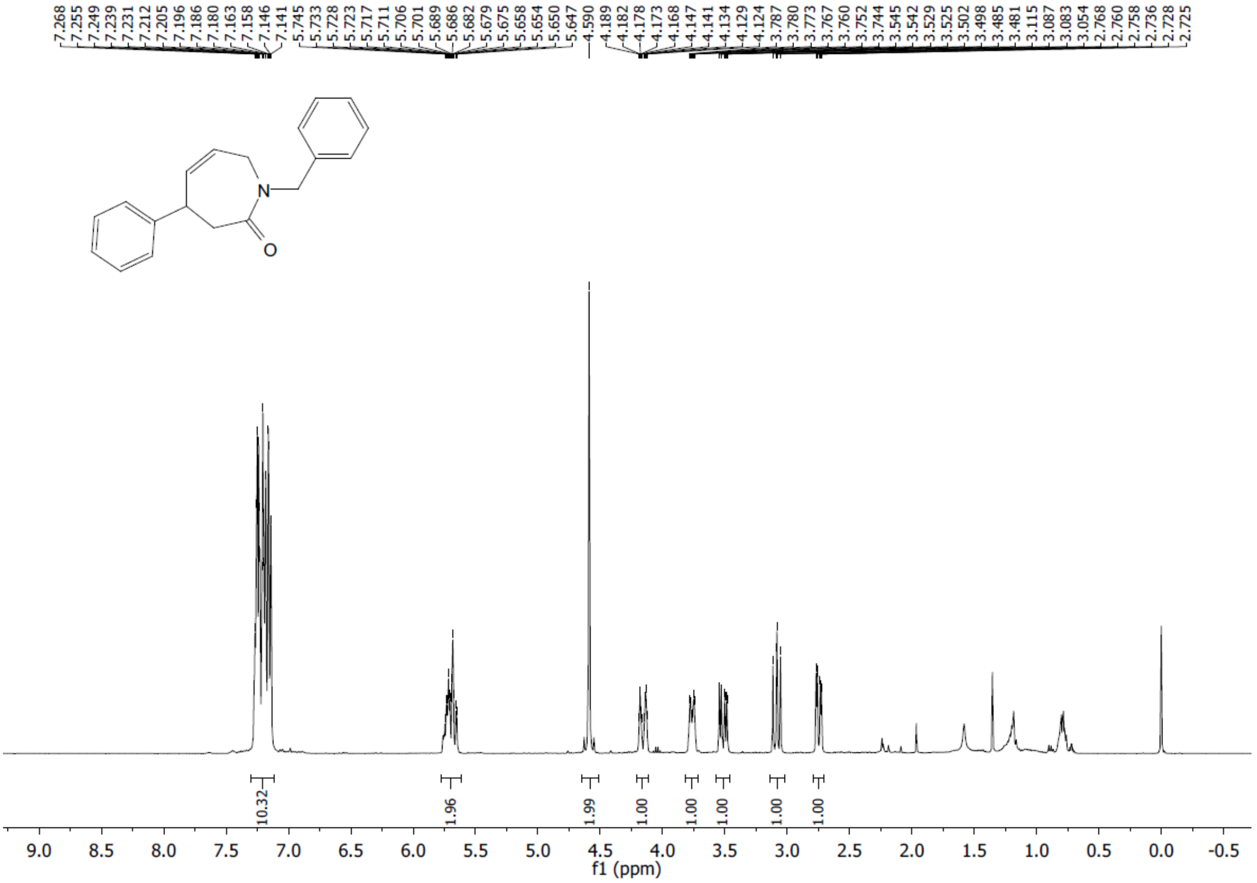

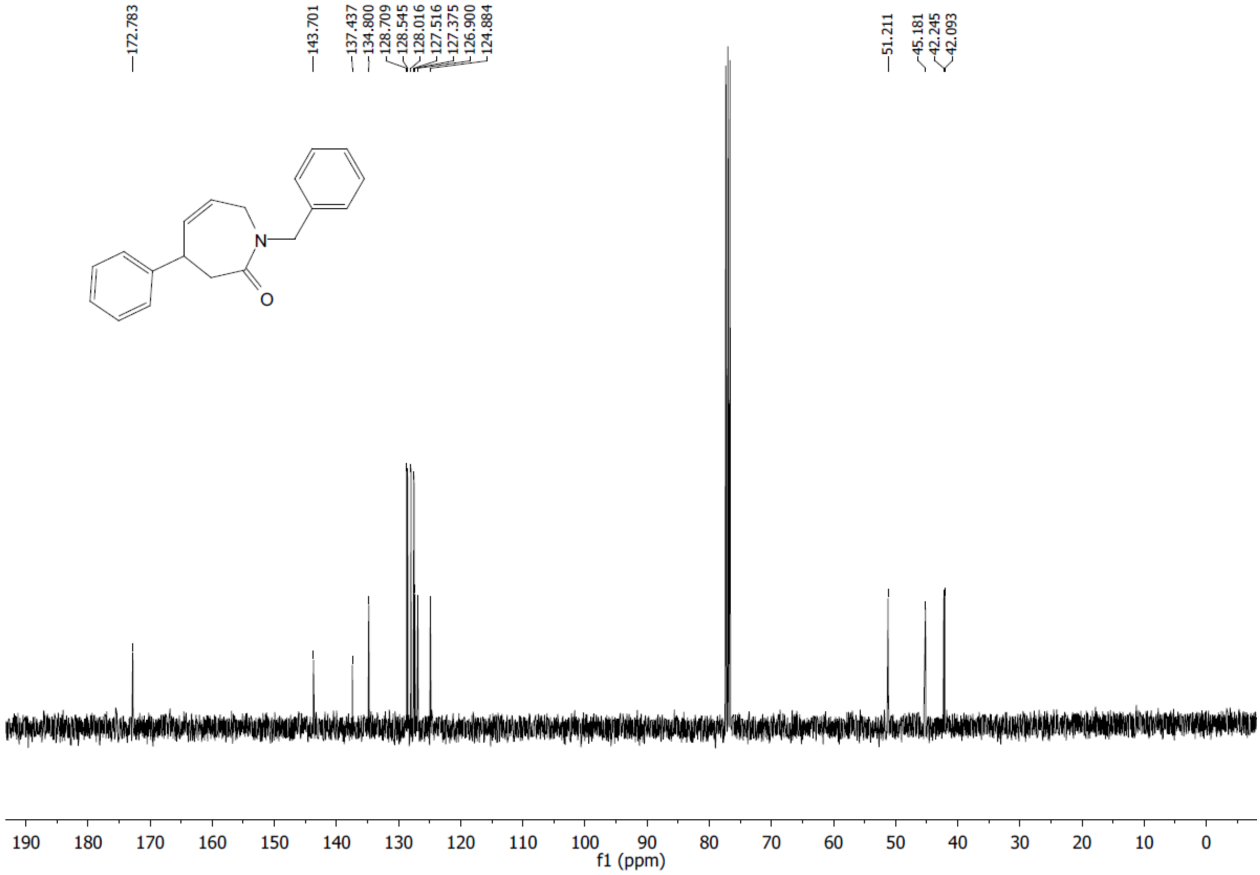

1-Benzyl-4-(4-bromophenyl)-1,3,4,7-tetrahydro-azepin-2-one (8b)

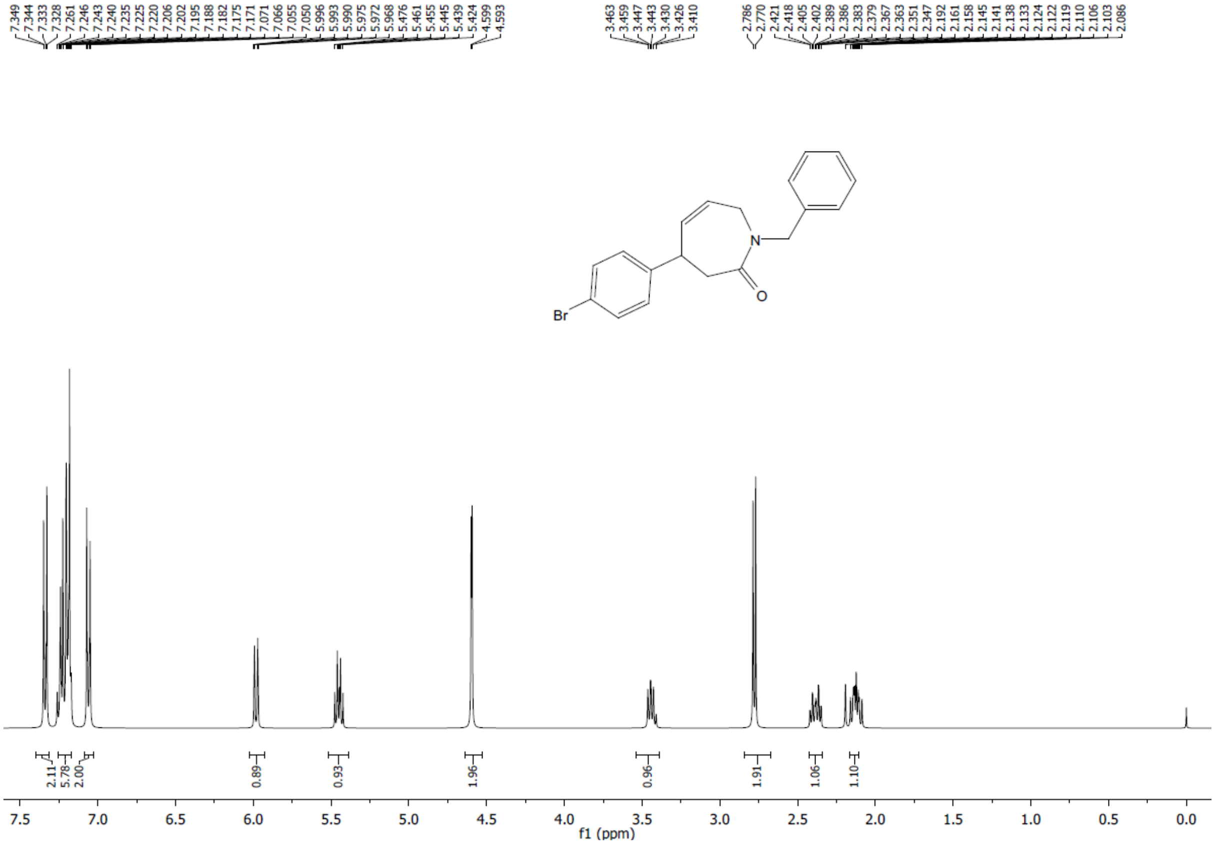

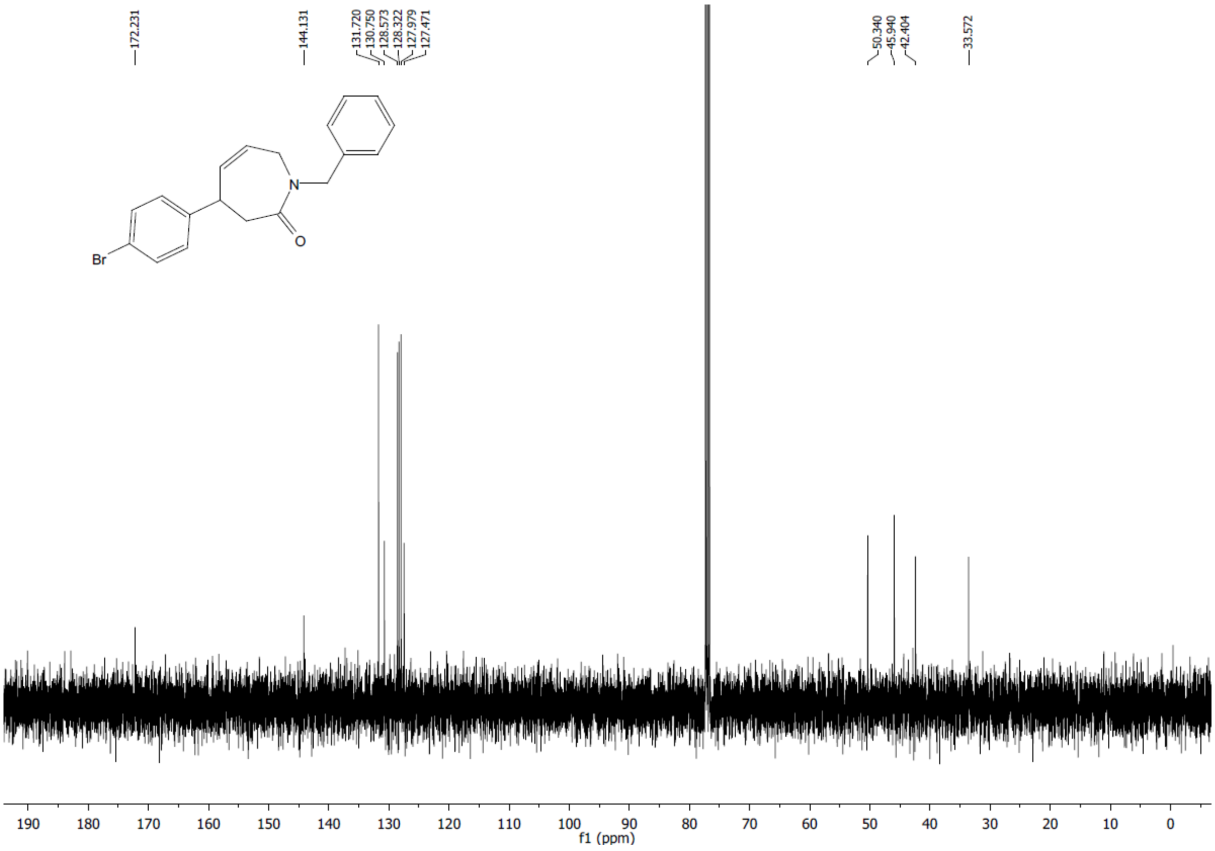

1-Benzyl-4-(4-chlorophenyl)-1,3,4,7-tetrahydro-azepin-2-one (8c)

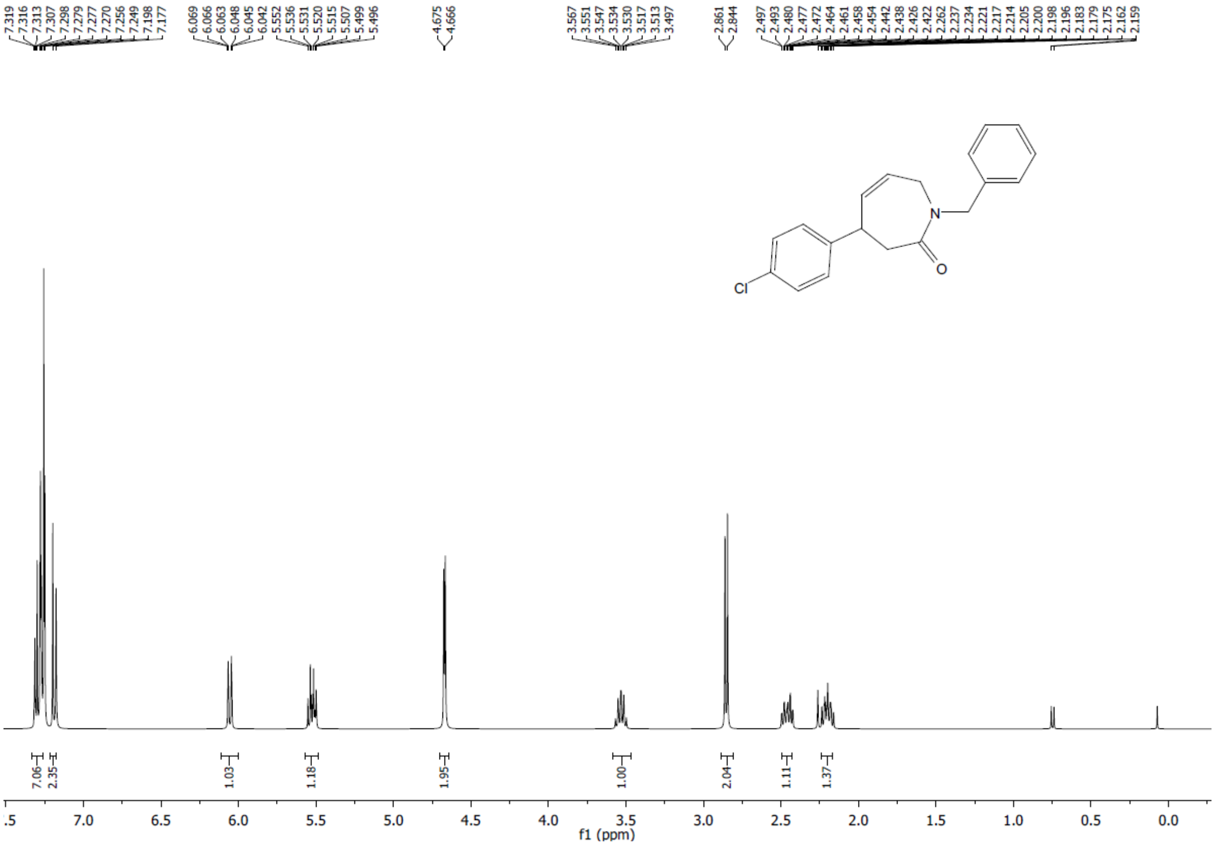

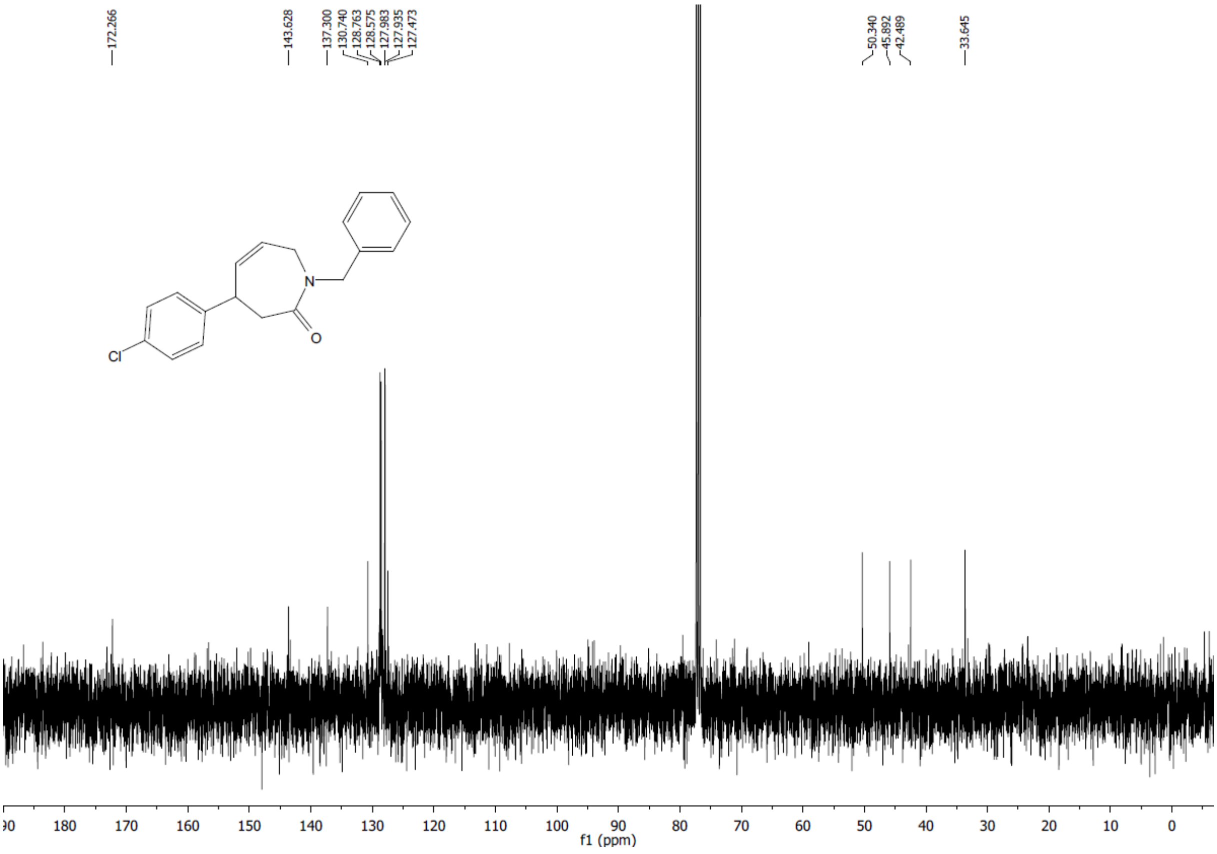

1-Benzyl-4-propyl-1,3,4,7-tetrahydro-azepin-2-one (8d)

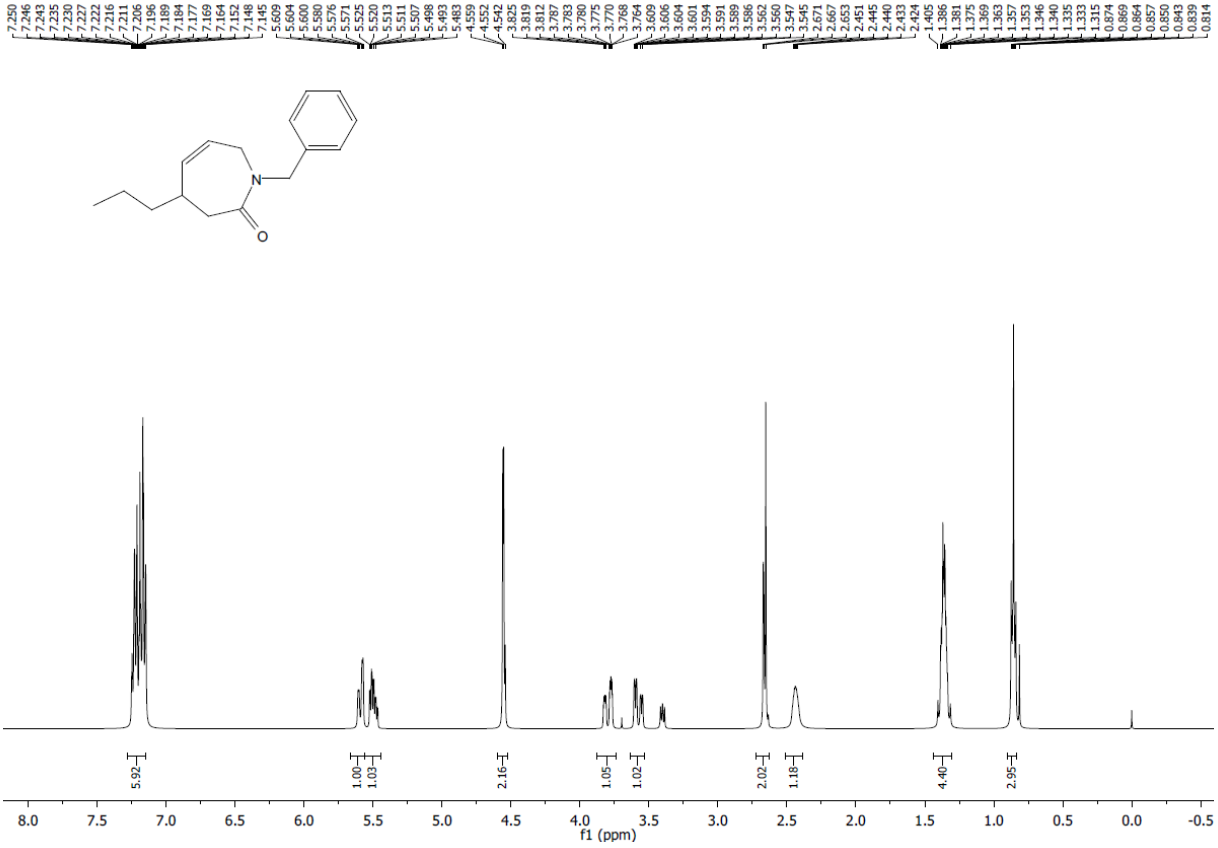

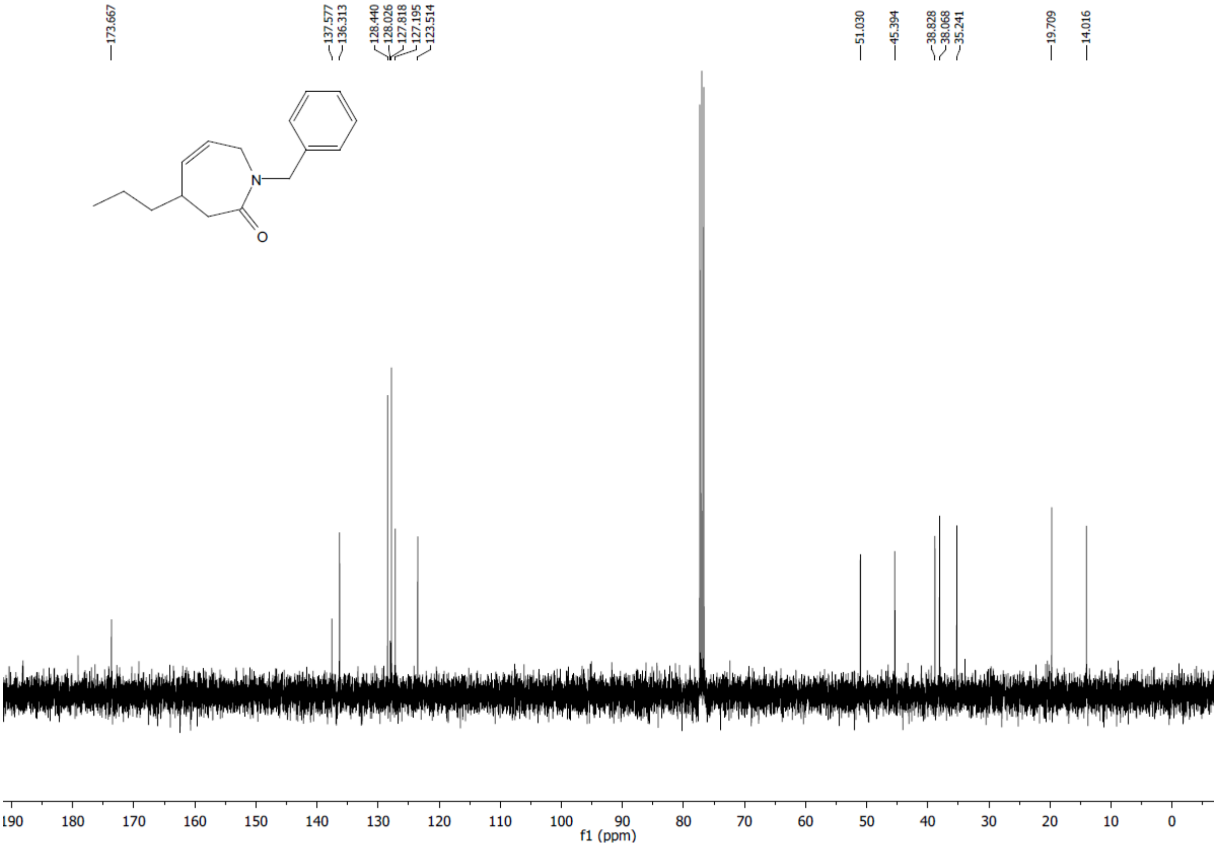

4-Phenyltetrahydro-2H-pyran-2-one (9)

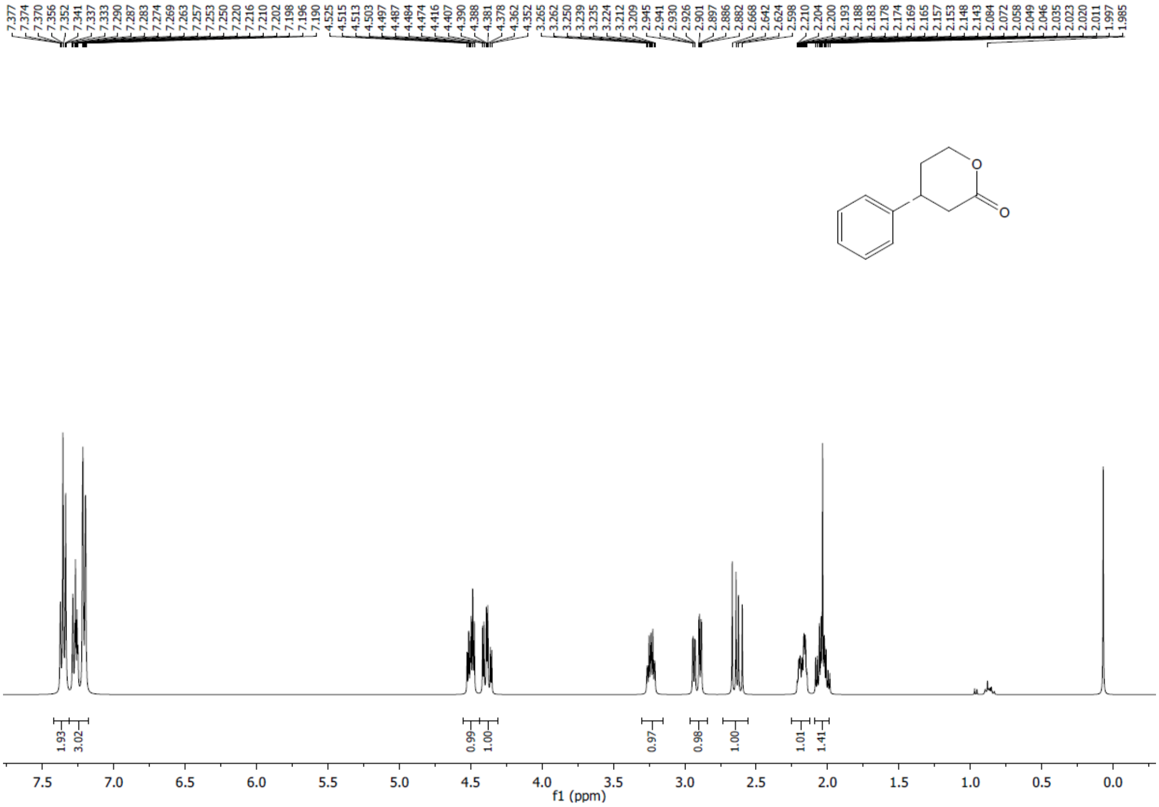

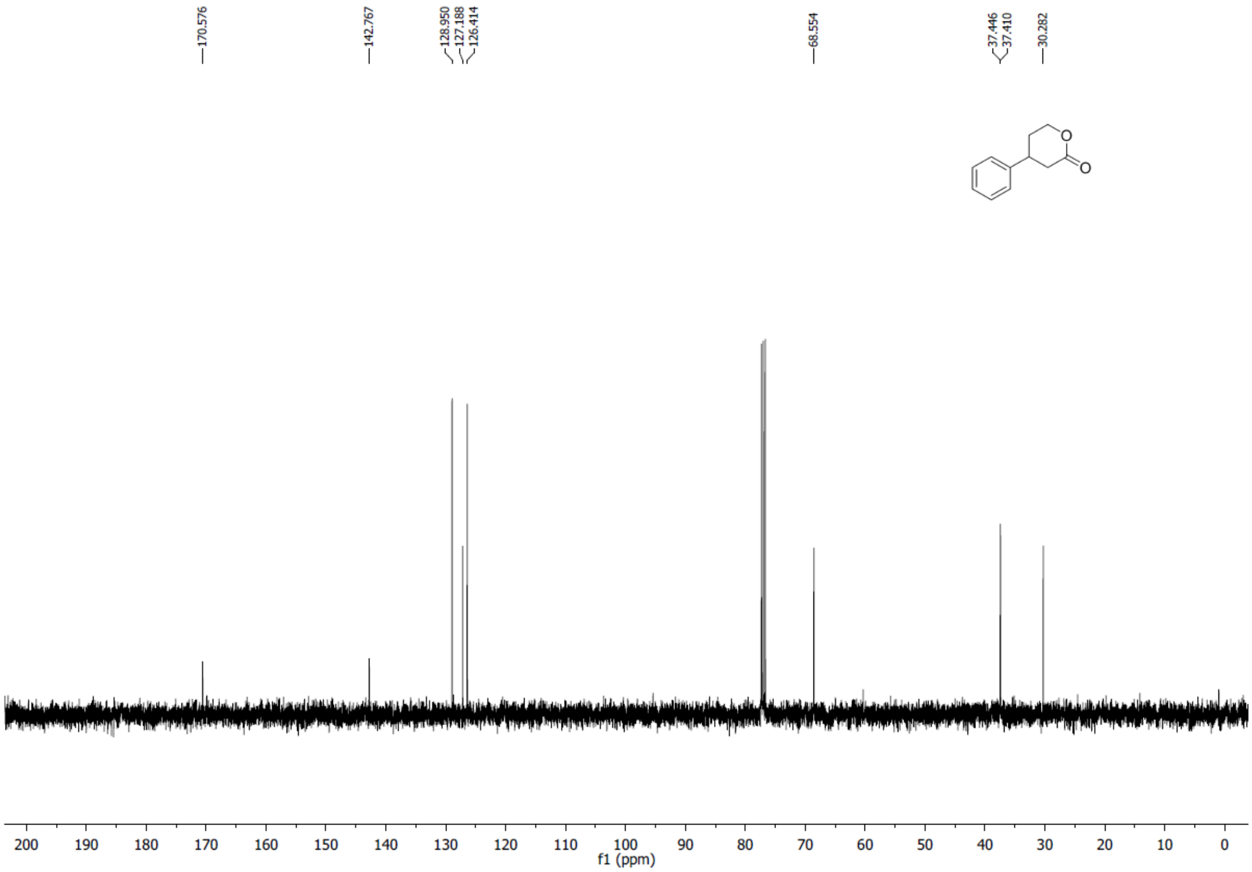

Methyl 5-oxo-3-phenylpentanoate (10)

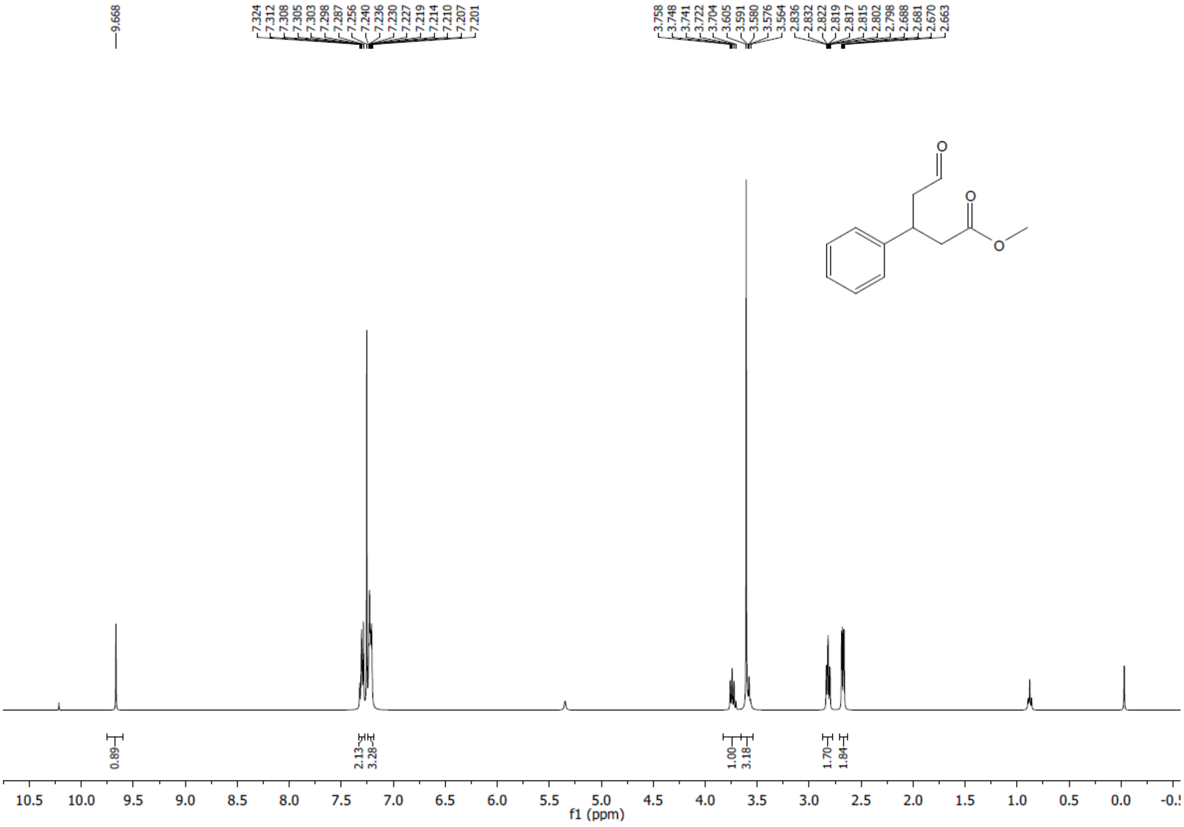

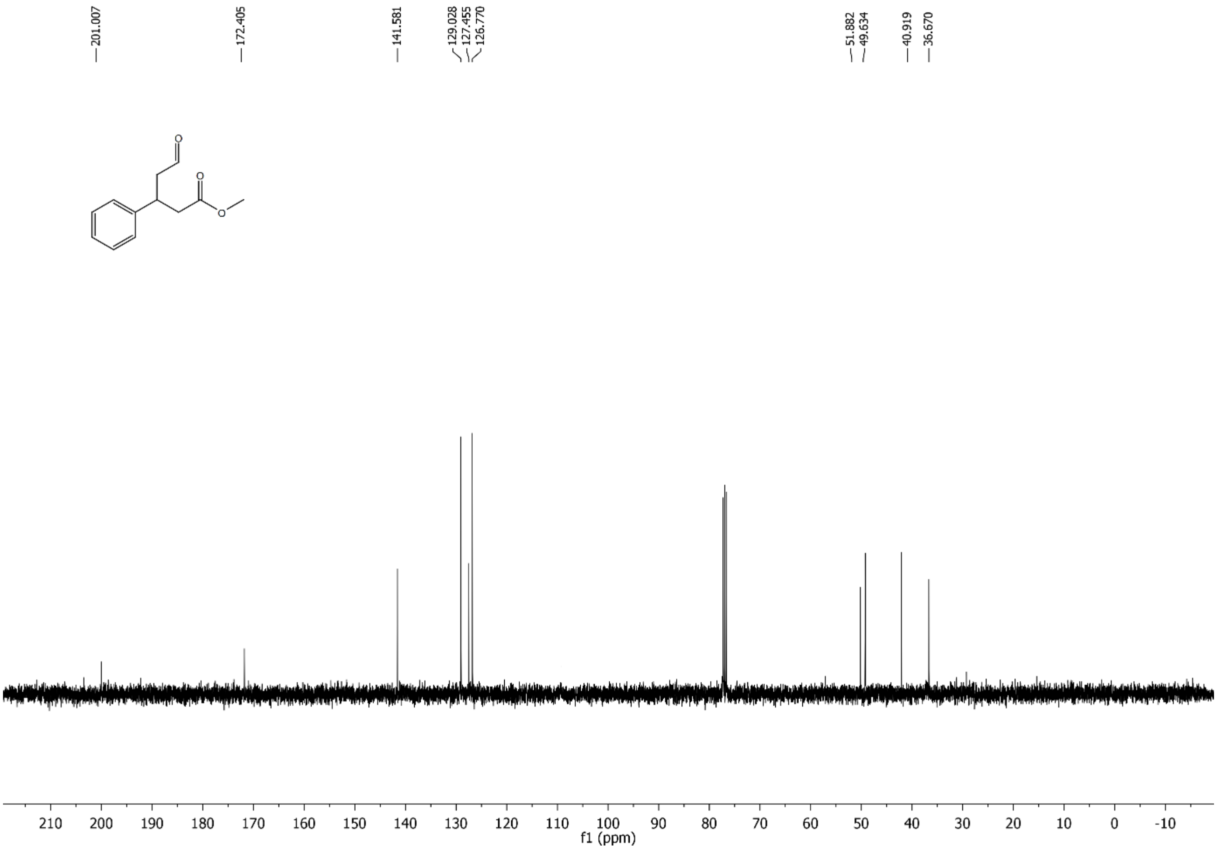

7. HPLC traces

Compound	Column	Mobile phase (Hep : IPA)	λ [nm]	mL/min	T °C	t_1 [min]	t_2 [min]
**1a**	OD-H	99 : 1	216	1.0	15	21.1	25.2
**4a**	AD-H	95 : 5	216	1.0	20	9.4	10.2
**4b**	Lux® 5 μm Amylose-1	97 : 3	216	1.0–10 min, then 0.5	20	22.5	24.0
**4d**	Lux® 5 mm Amylose-1	99 : 1	213	1.0	20	16.4	17.9
**5a**	AD-H	95 : 5	216	1.0	20	15.4	18.3
**5b**	Lux® 5 mm Amylose-1	97 : 3	216	1.0–10 min, then 0.5	20	29.5	33.9
**5d**	Lux® 5 mm Amylose-1	99 : 1	213	1.0	20	23.6	27.4
**7a**	AD-H	95 : 5	216	1.0	20	21.5	23.6
**8a**	AD-H	90 : 10	216	1.0	20	15.7	20.8
**8b**	Lux® 5 mm Amylose-1	90 :10	216	1.0	20	11.3	13.2
**8c**	Lux® 5 mm Amylose-1	94 : 6	213	1.0	20	19.9	21.3

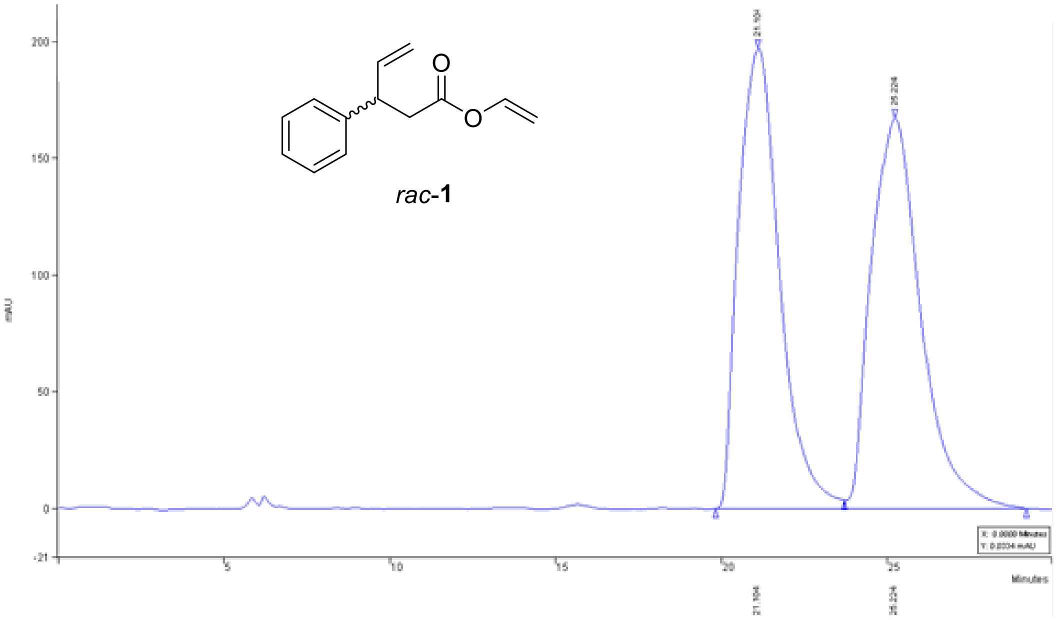

rac-1
mAU
Minutes

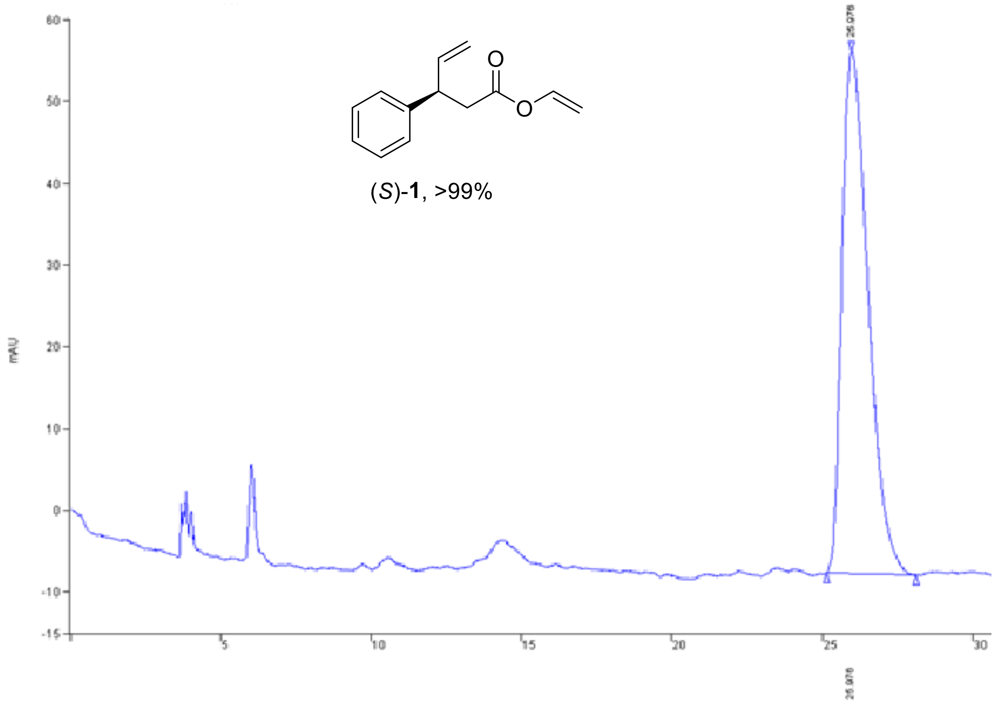

(S)-1, >99%
mAU

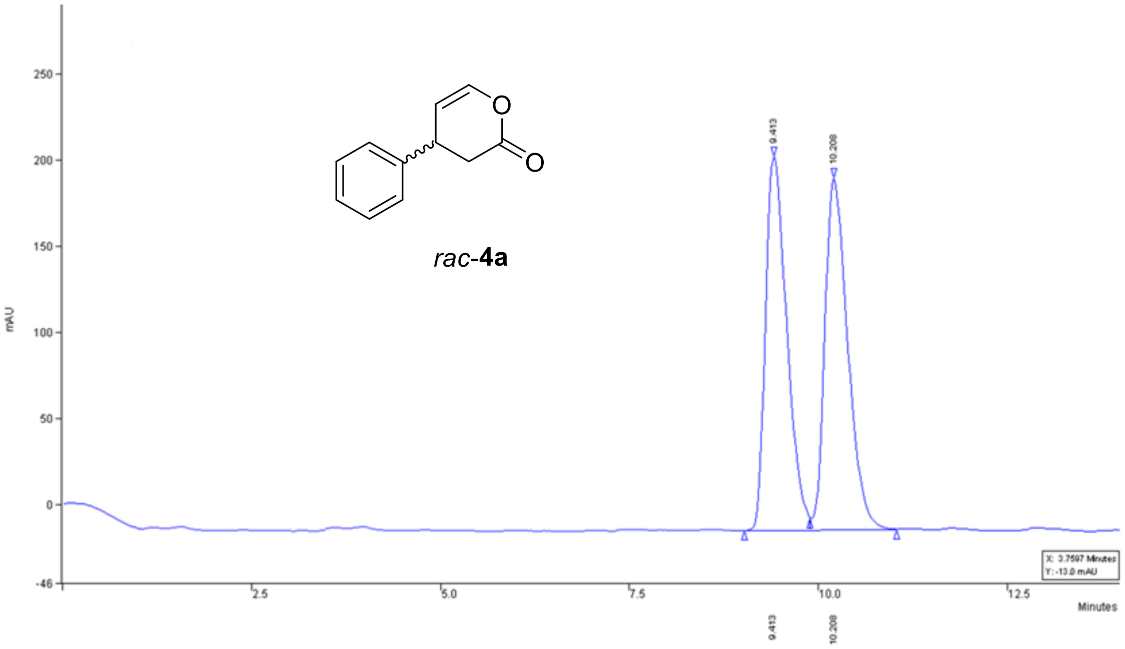

rac-4a
9.413
10.208
mAU
Minutes

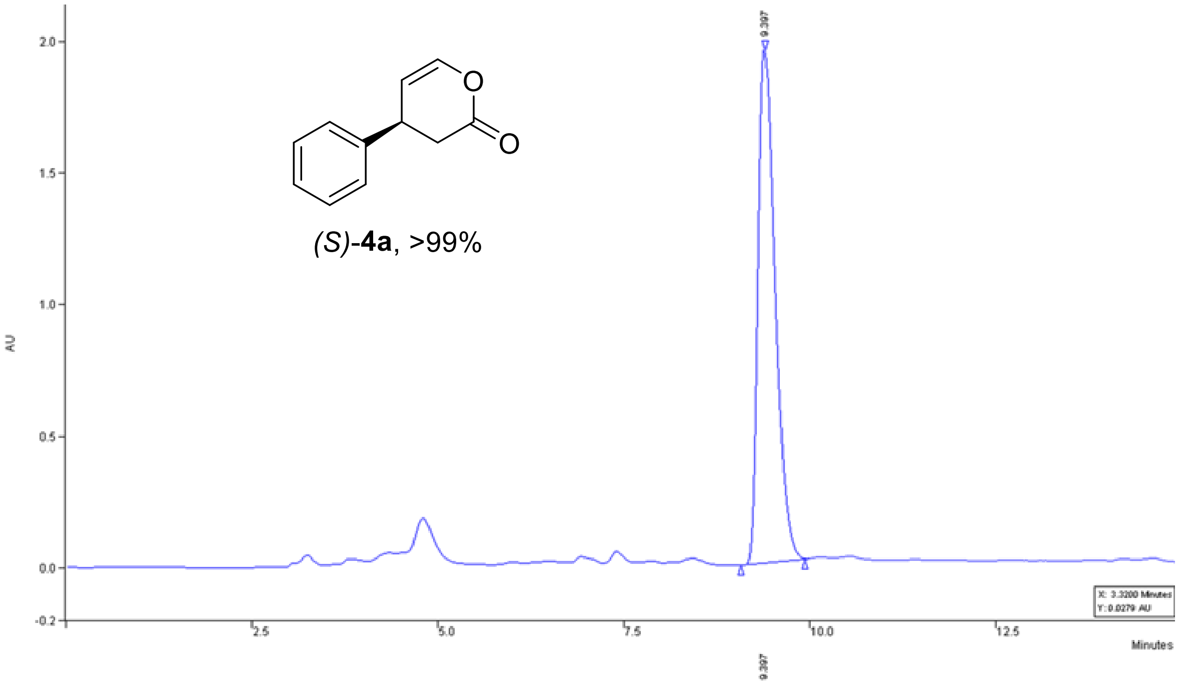

(S)-4a, >99%
9.397
AU
Minutes

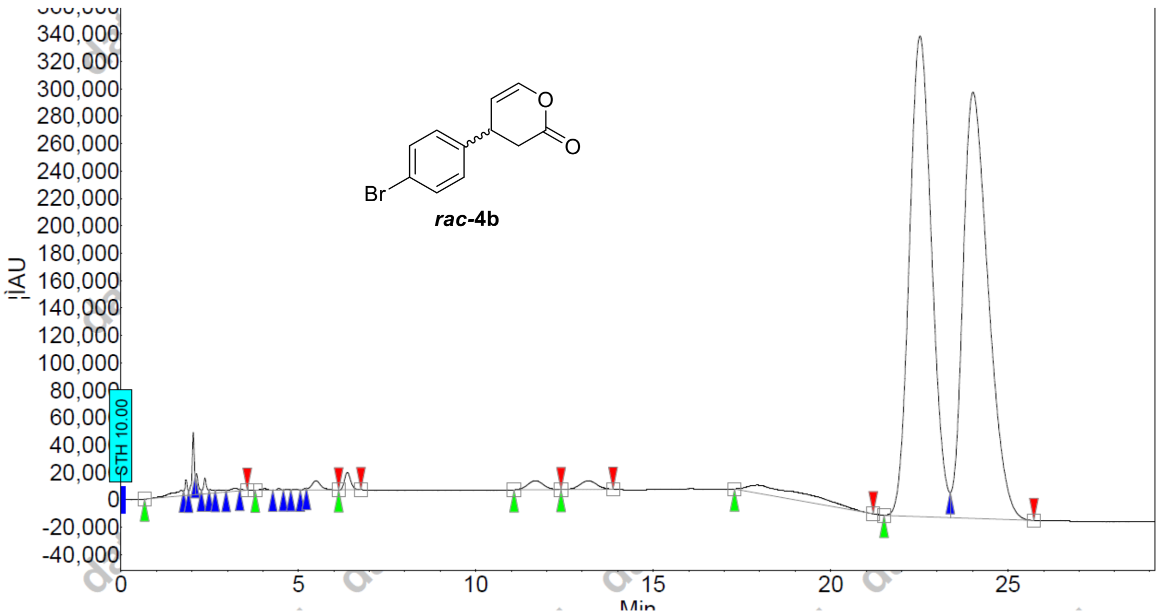
Br
rac-4b
μAU
Min
STH 10.00

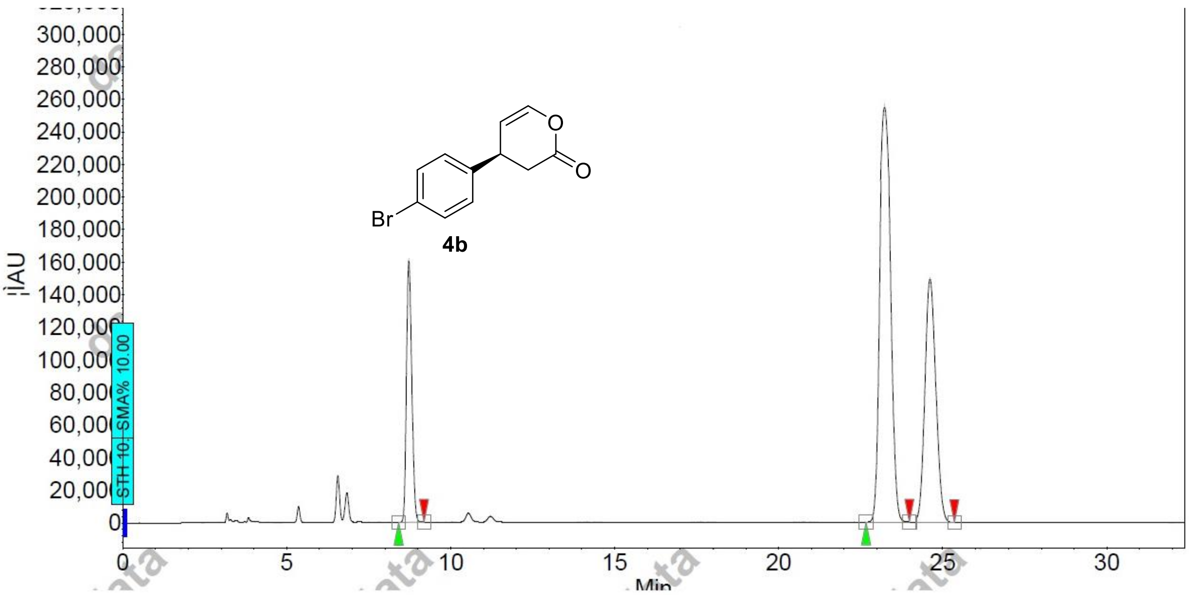
Br
4b
μAU
Min
STH 10 SMA% 10.00

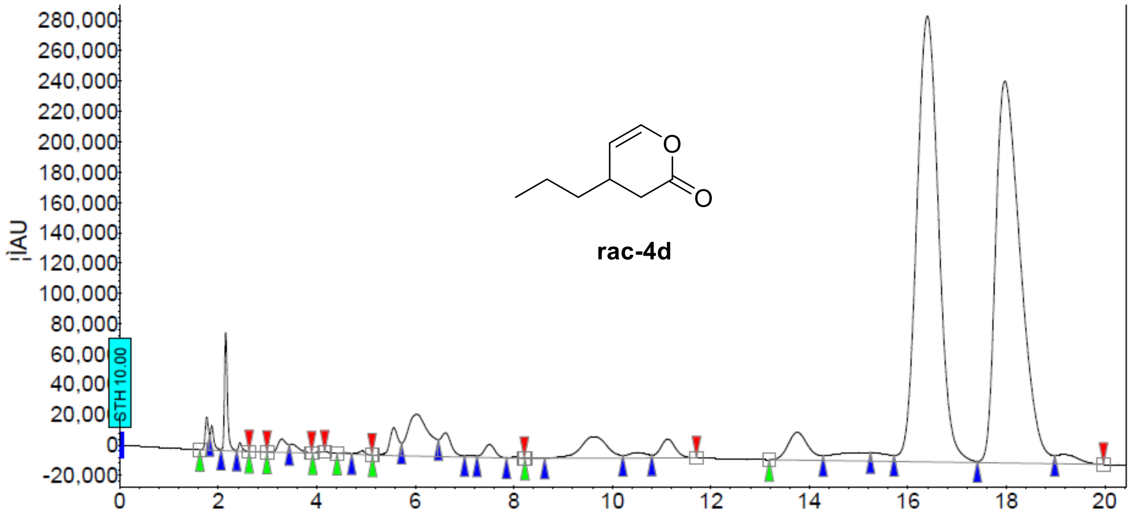

280,000
260,000
240,000
220,000
200,000
180,000
160,000
140,000
120,000
100,000
80,000
60,00
40,00
20,00
0
-20,000
¡IAU
STH 10.00
0
2
4
6
8
10
12
14
16
18
20

rac-4d

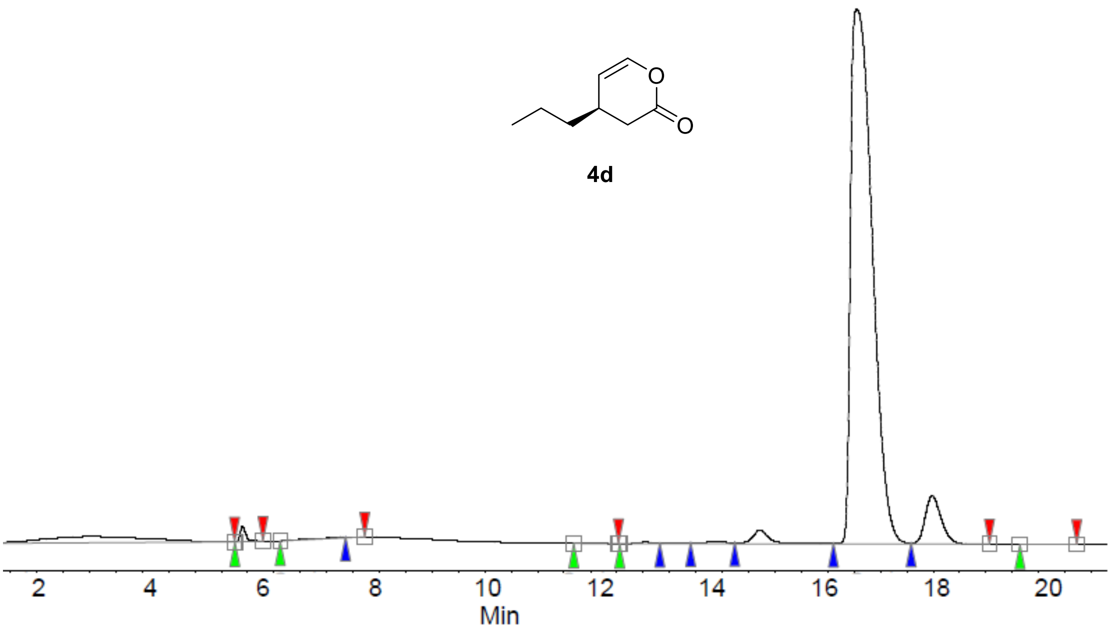

2
4
6
8
10
12
14
16
18
20
Min

4d

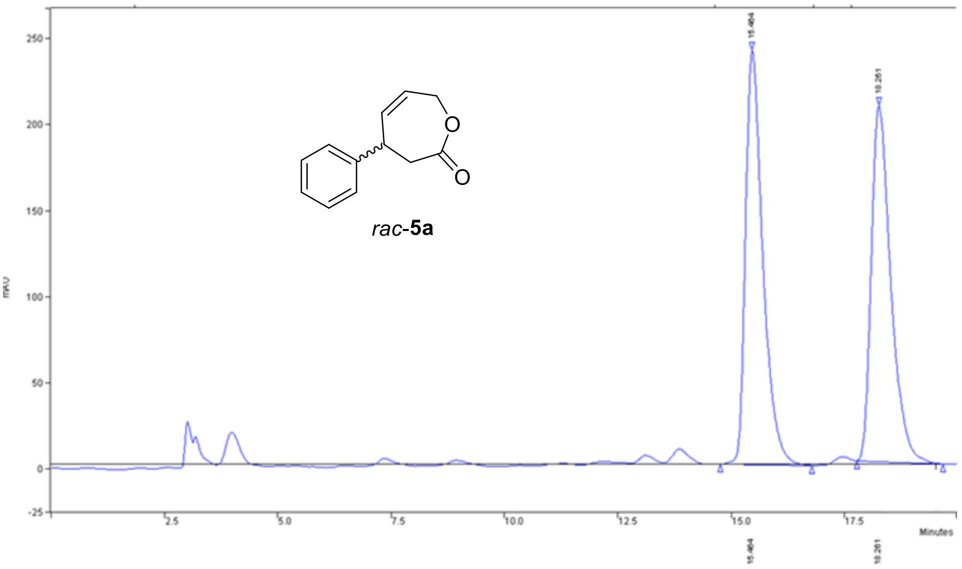
rac-5a
mAU
Minutes
15.464
18.261

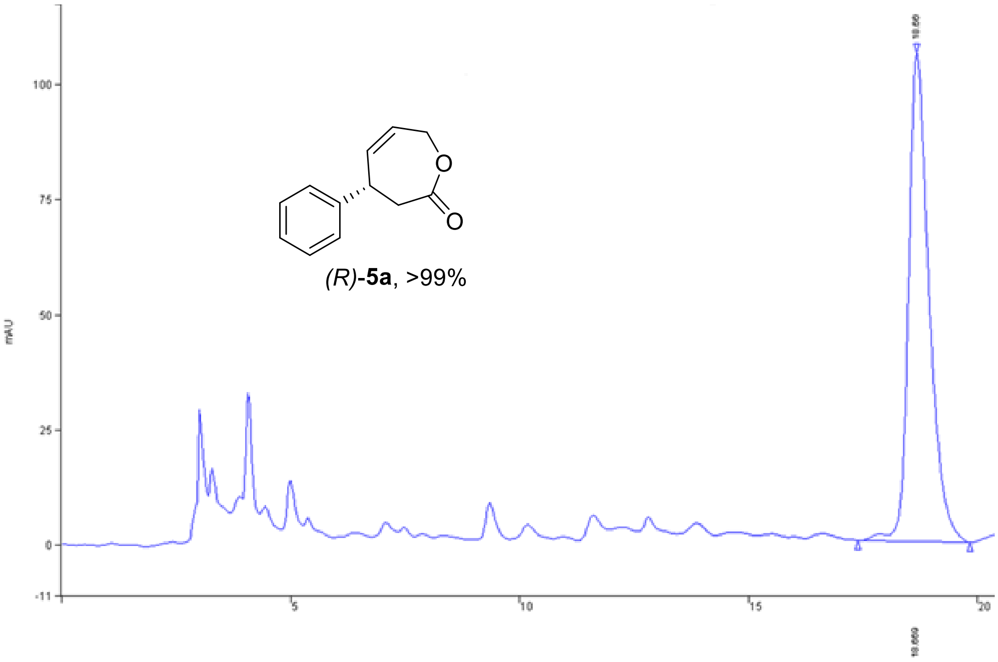
(R)-5a, >99%
mAU
18.669

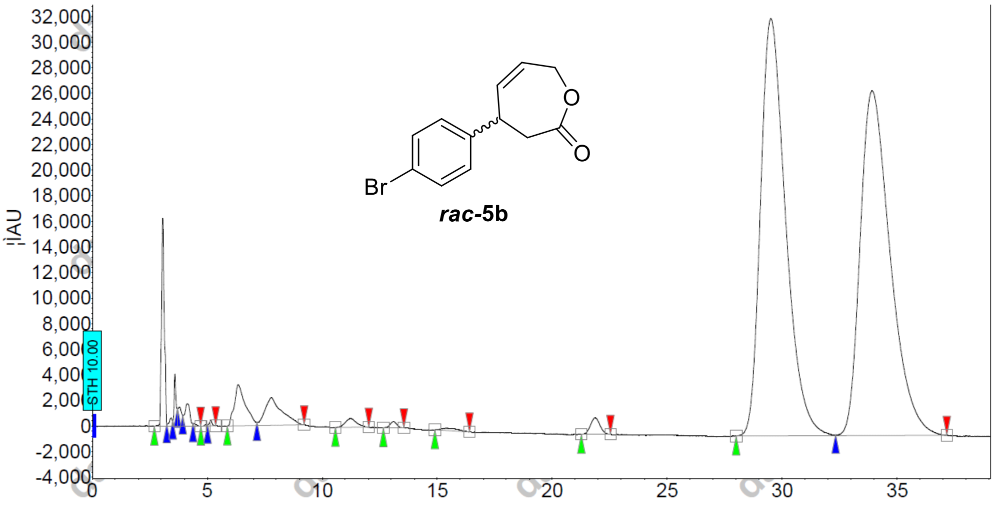
32,000
30,000
28,000
26,000
24,000
22,000
20,000
18,000
16,000
14,000
12,000
10,000
8,000
6,000
4,000
2,000
0
-2,000
-4,000
¡AU
STH 10.00
Br
O
O
rac-5b
0
5
10
15
20
25
30
35

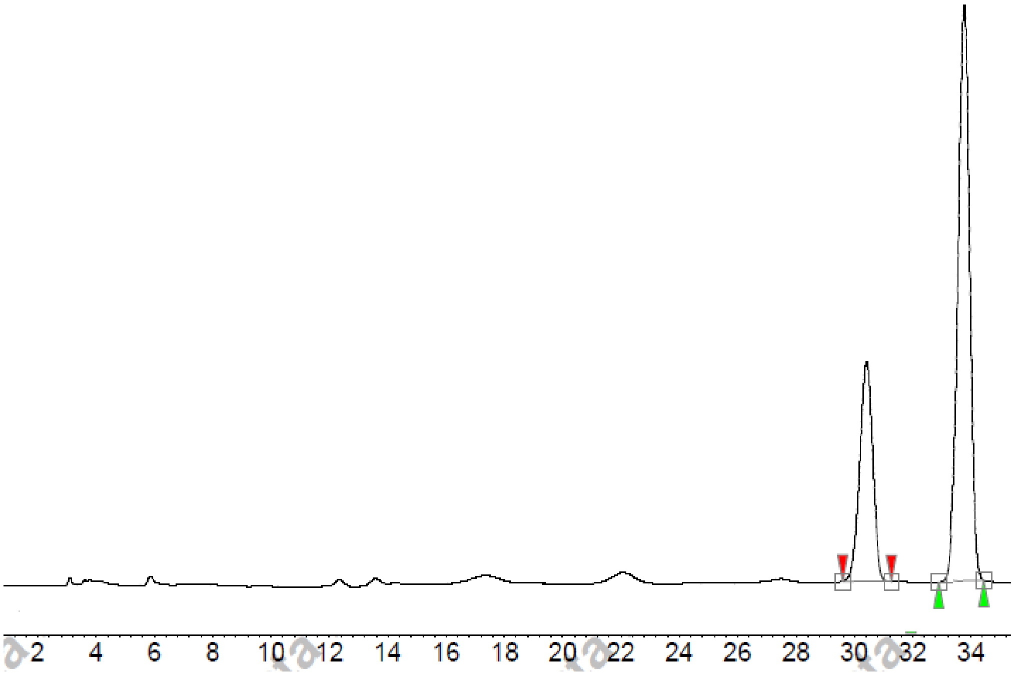
2
4
6
8
10
12
14
16
18
20
22
24
26
28
30
32
34

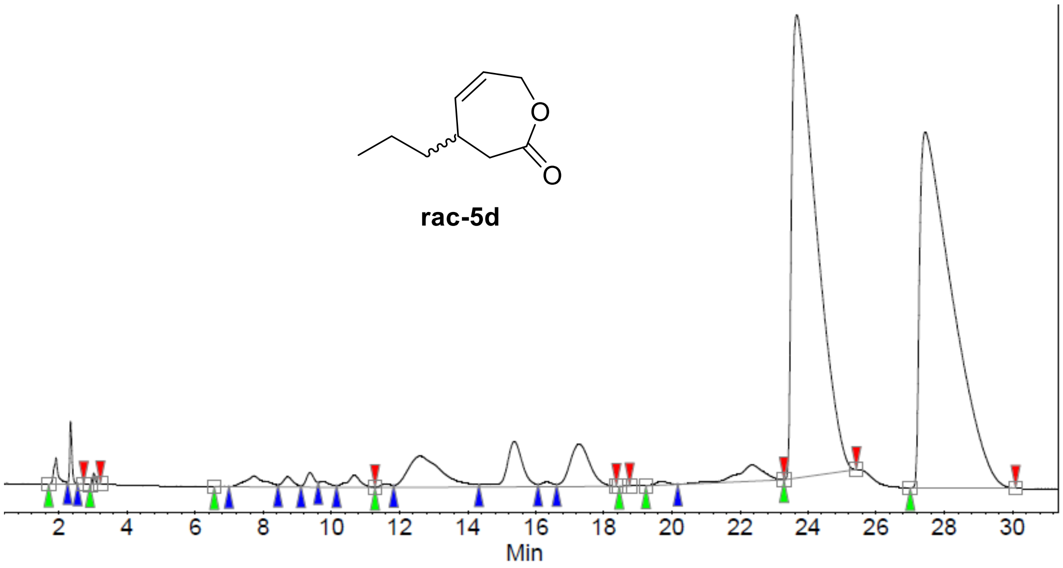

rac-5d
2
4
6
8
10
12
14
16
18
20
22
24
26
28
30
Min

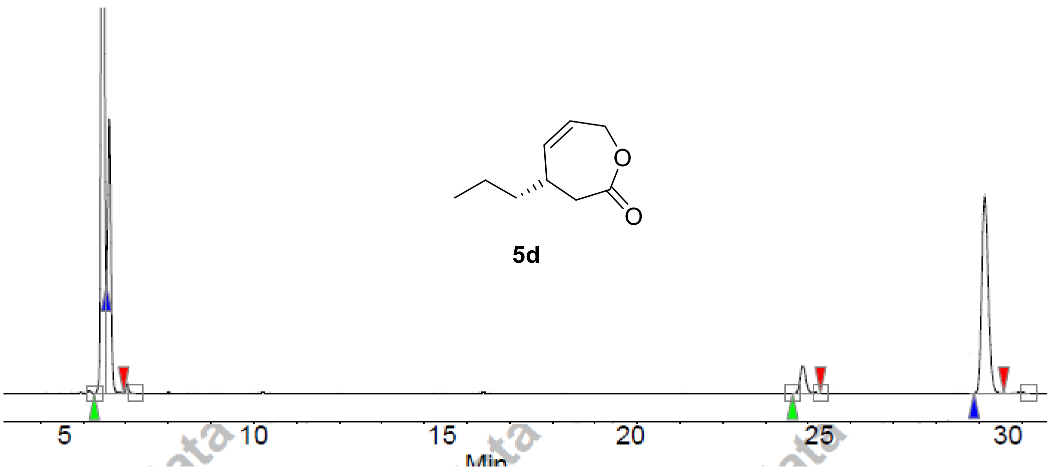

5d
5
10
15
20
25
30

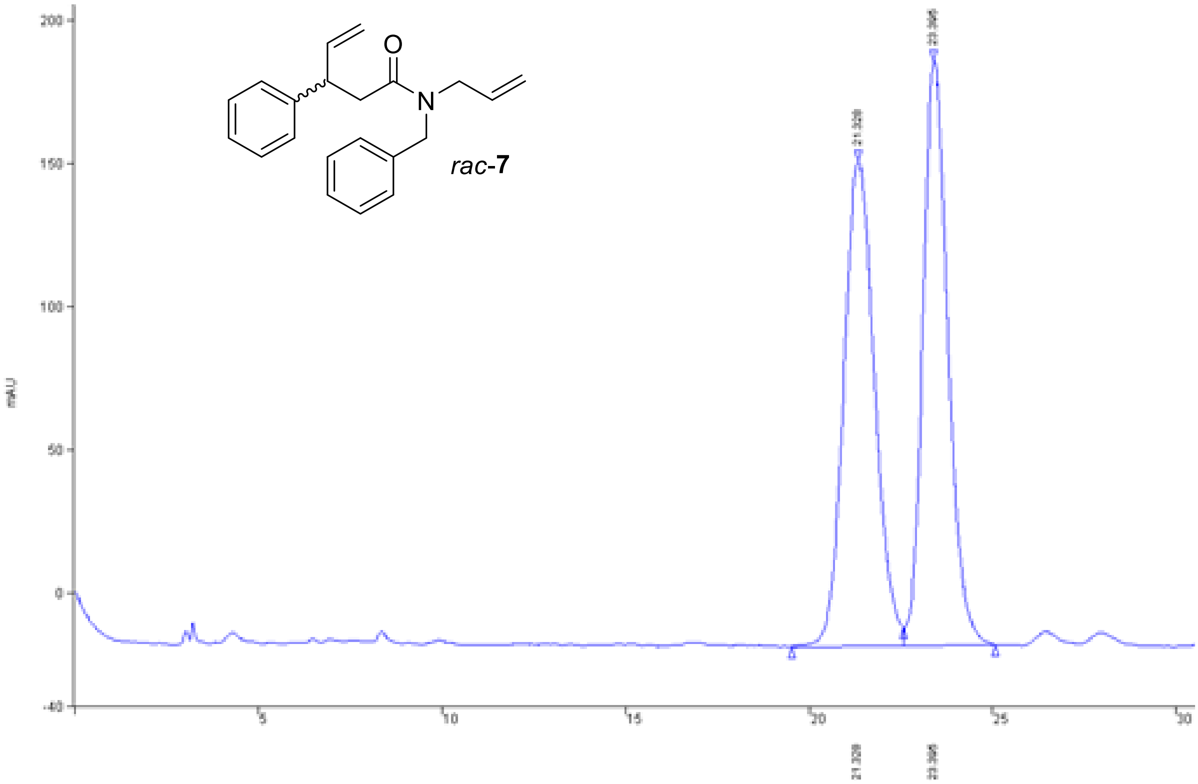

rac-7
mAU
200
150
100
50
0
-40
5
10
15
20
25
30
21.328
23.395

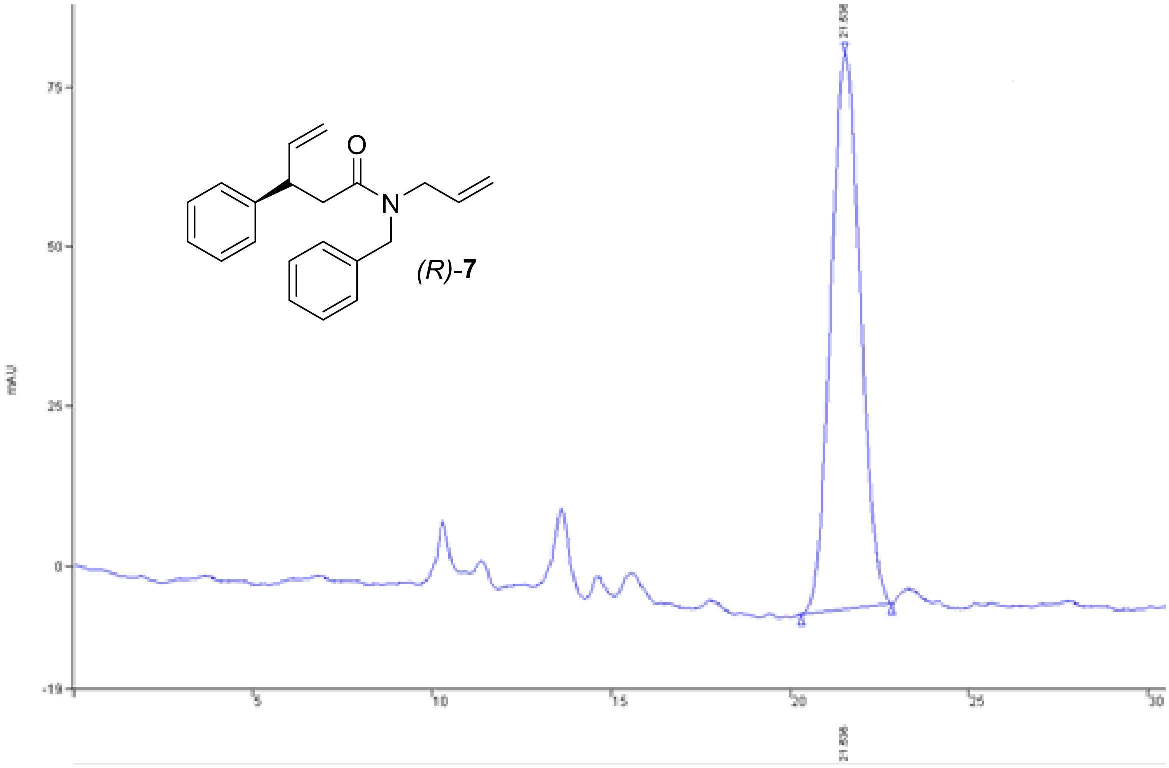

(R)-7
mAU
75
50
25
0
-19
5
10
15
20
25
30
21.536

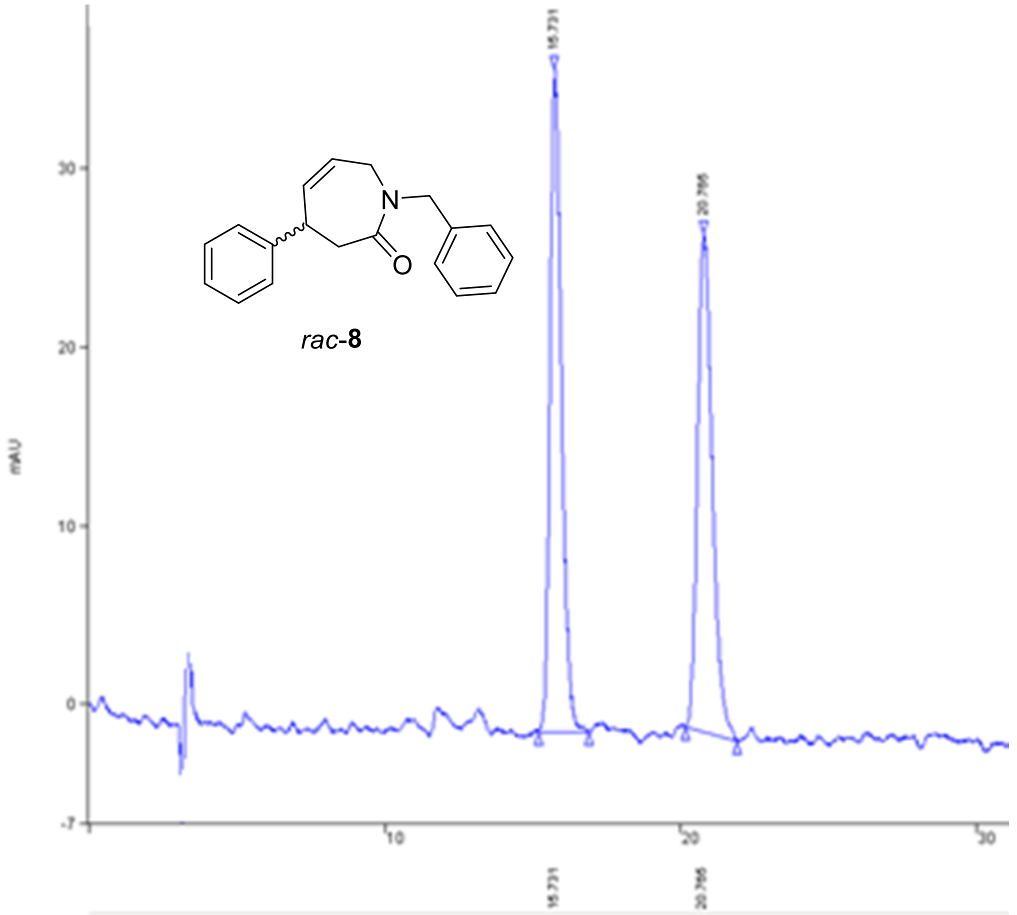
rac-8
N
O
mAU
15.731
20.766

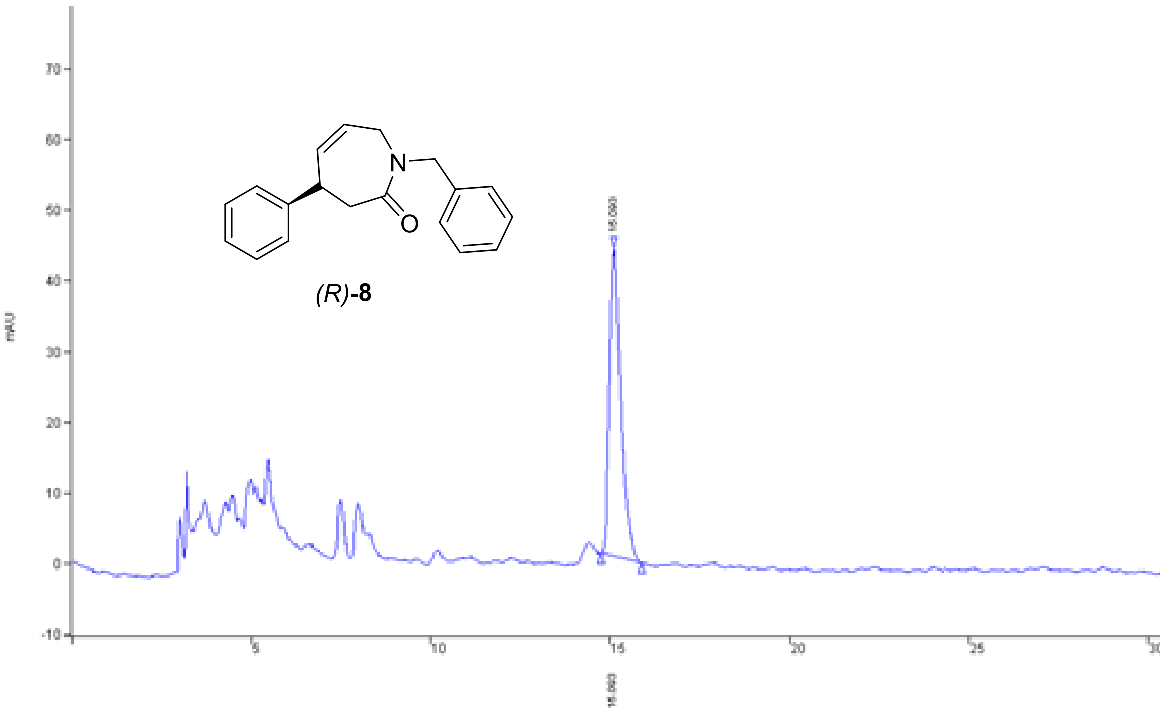
(R)-8
N
O
mAU
15.093

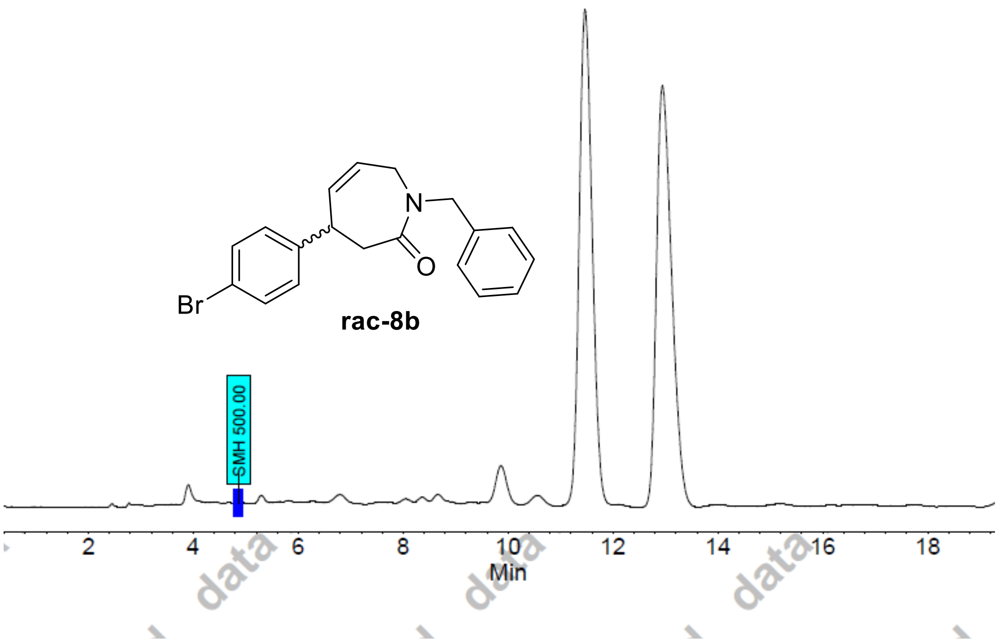

Br
N
O
rac-8b
SMH 500.00
2
4
6
8
10
12
14
16
18
Min

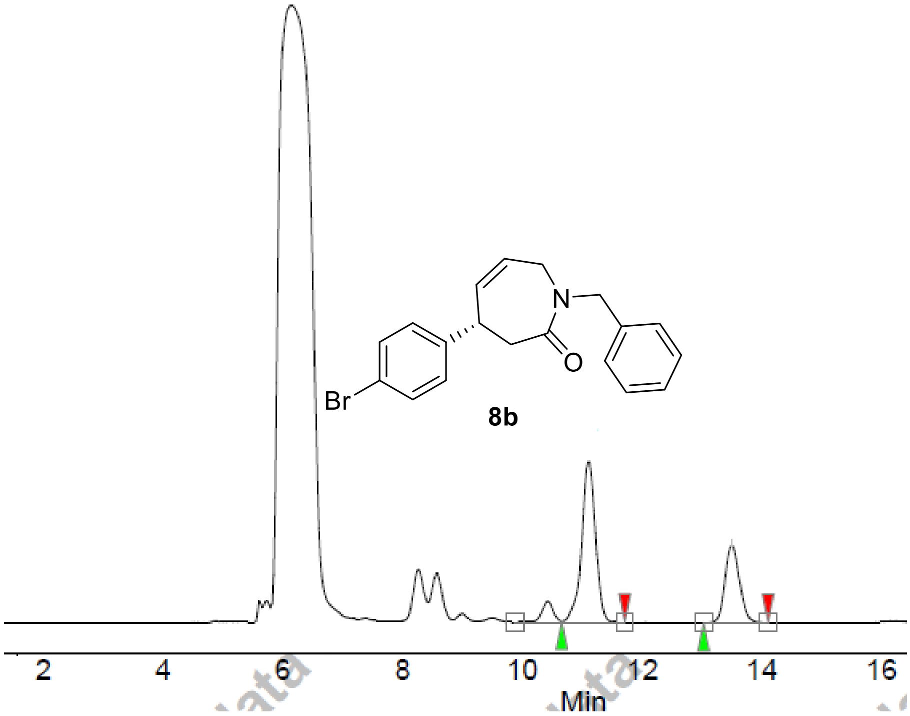

Br
N
O
8b
2
4
6
8
10
12
14
16
Min

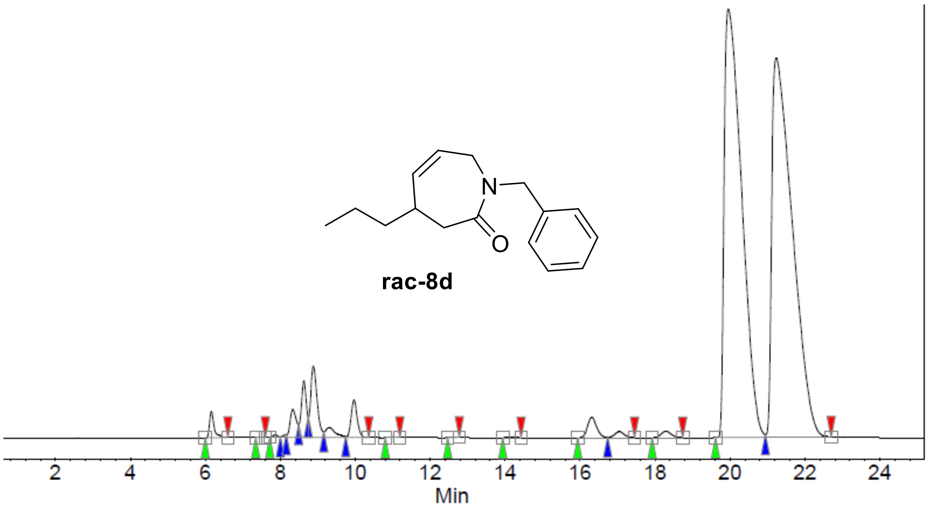

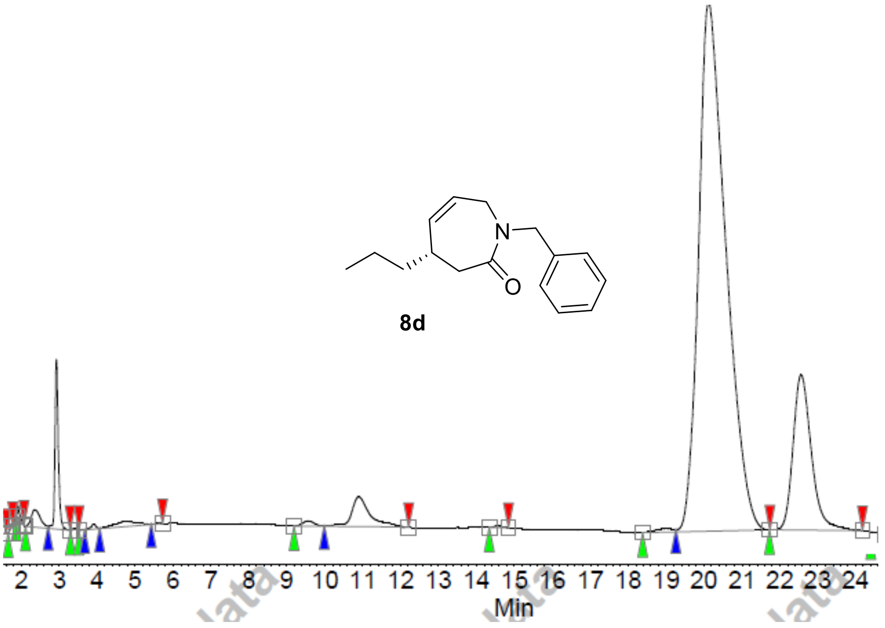

8. References

1 Gao, M.; Wang, D.-X.; Zheng, Q.-Y.; Wang, M.-W.; An Unusual β-Vinyl Effect Leading to High Efficiency and Enantioselectivity of the Amidase, Nitrile Biotransformations for the Preparation of Enantiopure 3-Arylpent-4-enoic Acids and Amides and Their Applications in Synthesis. *J. Org. Chem*. **2006,** *71*, 9532-9535.

2 (a) Brodzka, A.; Borys, F.; Koszelewski, D.; Ostaszewski, R.; Studies on the Synthesis of Endocyclic Enol Lactones via a RCM of Selected Vinyl Esters. J. Org. Chem. 2018, 83, 8655-8661; (b) Koszelewski, D.; Brodzka, A.; Żądło, A.; Paprocki, D.; Trzepizur, D.; Zysk, M.; Ostaszewski, R.; Dynamic Kinetic Resolution of 3-Aryl-4-pentenoic Acids. *ACS Catal.* **2016**, *6*, 3287-3292.

3 Brodzka, A.; Koszelewski, D.; Ostaszewski, R.; Dual Activity of Grubbs-Type Catalyst in the Transvinylation of Carboxylic Acids and Ring-Closing Metathesis Reactions. J. Org. Chem. 2020, 85, 15305-15313.

4 Dong, J; Liu, Y.; Cui, Y.; Chiral porous organic frameworks for asymmetric heterogeneous catalysis and gas chromatographic separation; *Chem. Commun.* **2014**, *50*, 14949-14952.

5 Basu, S.; Gupta, V.; Nickel, J.; Schneider, C. Organocatalytic Enantioselective Vinylogous Michael Reaction of Vinylketene Silyl-*N,O*-Acetals; *Org. Lett.* **2014**, *16,* 274.
